# Controlling Chemoselectivity of Catalytic Hydroboration with Light

**DOI:** 10.1002/anie.202114482

**Published:** 2022-01-11

**Authors:** Enrico Bergamaschi, Danijela Lunic, Liam A. McLean, Melissa Hohenadel, Yi‐Kai Chen, Christopher J. Teskey

**Affiliations:** ^1^ Institute of Organic Chemistry RWTH Aachen University Landoltweg 1 52074 Aachen Germany

**Keywords:** Chemoselectivity, Cobalt, Hydroboration, Photochemistry, Reduction

## Abstract

The ability to selectively react one functional group in the presence of another underpins efficient reaction sequences. Despite many designer catalytic systems being reported for hydroboration reactions, which allow introduction of a functional handle for cross‐coupling or act as mild method for reducing polar functionality, these platforms rarely deal with more complex systems where multiple potentially reactive sites exist. Here we demonstrate, for the first time, the ability to use light to distinguish between ketones and carboxylic acids in more complex molecules. By taking advantage of different activation modes, a single catalytic system can be used for hydroboration, with the chemoselectivity dictated only by the presence or absence of visible light.

## Graphical abstract

Light‐controllable, chemoselective hydroboration of ketoacids is reported using a well‐defined cobalt hydride catalyst. Excellent selectivities can be achieved, with acids reacting in the dark and ketones in the light. This approach can be further extended to other functional groups.

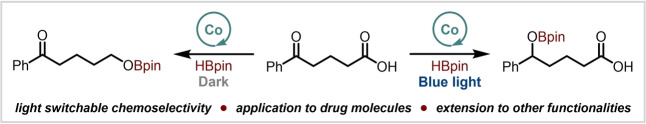

The development of new chemoselective transformations continues to attract significant attention.^[1]^ Strategies that allow selective reaction of a traditionally less reactive functional group in presence of a more reactive functional group avoid often tenuous protecting group manipulations, leading to more efficient synthetic routes.^[2]^ In the arena of catalysis, increased interest has arisen in chemodivergent methods which allow easy generation of new structurally diverse chemicals from common intermediates.^[3]^ These methods typically rely on varying a parameter such as catalyst,^[4]^ ligand^[5]^ or reagent.^[6]^

As a non‐invasive stimulus which offers excellent spatial‐temporal control, light would be particularly attractive to control selectivity.^[7]^ However, despite the rapidly growing interest in photochemical methods, light is still most often used to promote reactions that either take place very slowly, or cannot occur at all in the dark.^[8]^ Previous examples of modifying the selectivity of metal catalysed processes are limited in number but have taken a variety of different approaches.^[9]^ For instance, light responsive motifs can be incorporated into the ligand structure^[10]^ or direct excitation can lead to a change in redox properties^[11]^ amongst other strategies.^[12]^

Our group has recently exploited light induced modification of the coordination sphere of a cobalt hydride catalyst (Scheme 1a) to control ambidoselectivity in the hydroboration of α,β‐unsaturated ketones (Scheme 1b),^[13]^ and this idea has begun to attract more attention for opening up new mechanistic platforms.^[14]^ Given the different reactivity that is observed in dark vs. light, we wondered if it may be possible to develop this into a new concept whereby different functional groups could be targeted depending only on the presence or absence of light.^[15]^

**Scheme 1:**
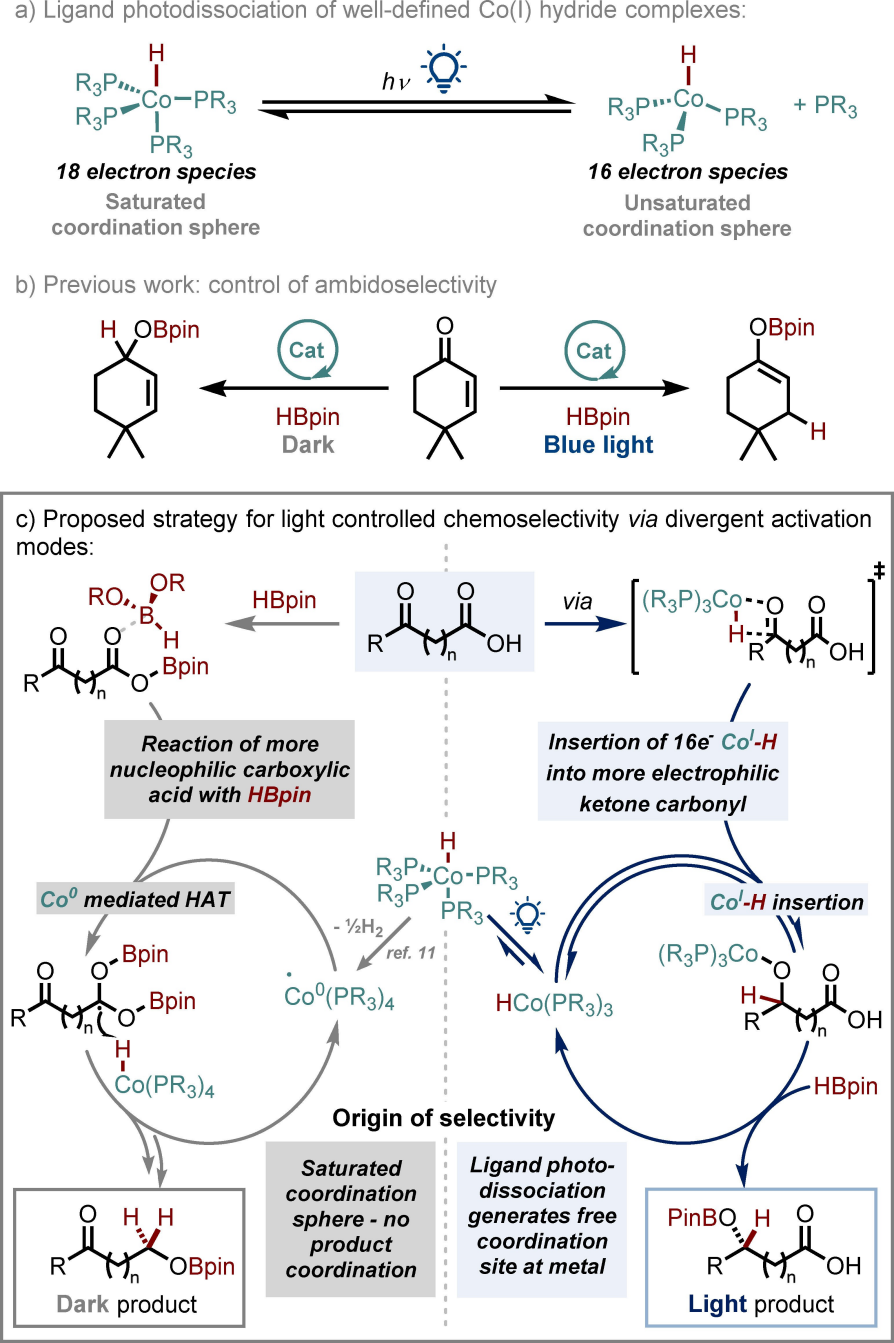
Planned divergence of activation mode to allow light‐controlled chemoselective hydroboration.

Our plan began by considering hydroboration as a model reaction after we noted that, despite the many reports in recent years, almost none of these publications investigate more complex molecules which contain multiple functionalities,^[16]^ instead focussing on other aspects such as catalyst development,^[17]^ regio‐^[18]^ or enantioselectivity.^[19]^ Using cobalt hydride catalysts of the type shown in Scheme 1a, we hypothesized that in absence of light irradiation, the saturated cobalt centre would not be able to coordinate the substrate directly but, as we previously reported,^[13]^ would be reduced to Co^0^ under the proposed conditions, and thus act as a catalyst for intramolecular hydrogen atom transfer (Scheme 1c, left). As such, the initial step would be coordination to the Lewis acidic borane by the more electron‐rich carboxylic acid carbonyl, activating it to hydroboration in preference to the ketone (Scheme 1c, left)^[20]^ and enabling a mild catalytic method for carboxylic acid reduction.^[21]^

In contrast, photodissociation under light irradiation would allow reversible coordination of the substrate to the 16‐electron cobalt(I) hydride species which would undergo a selective coordination/insertion sequence into the more electrophilic ketone carbonyl (Scheme 1c, right).[^22^, ^23^] Such photo‐divergence would offer a completely new strategy to alter the chemoselectivity of a reaction. On‐demand selectivity would be available by simply switching the light on or off with no other change in conditions required. To this end, we began our investigations with 4‐benzoylbutyric acid, **1 a**, containing both a ketone and a carboxylic acid, and pinacolborane—a widely available hydroborating agent that has been reported to reduce a range of carbonyl functional groups,^[24]^ even without catalysts.^[25]^ However, when we carried out the reaction of **1 a** with HBpin and no catalyst, we obtained a moderate yield of a mixture of three products with very poor selectivity (Scheme 2a, entries 1 and 2). In complete contrast, after some optimization, we were delighted to observe that when using 5 mol% of CoH[PPh(OEt)_2_]_4_ as a catalyst for this same transformation, we obtained excellent selectivity either for the carboxylic acid hydroboration product, **2 a** without light irradiation (Scheme 2a, entry 3) or for the ketone hydroboration product, **3 a**, under blue light irradiation (Scheme 2a, entry 4).^[26]^ To the best of our knowledge, this demonstrates the first example of being able to control chemoselectivity of a transformation with light whilst also highlighting the ability of the catalyst to tune the properties of the uncatalyzed, unselective reaction. Other state of the art methods for carbonyl hydroboration^[27]^ or carboxylic acid reduction^[28]^ were also unable to match the selectivity of either of our conditions (Scheme 2a, entries 5 and 6).

**Scheme 2:**
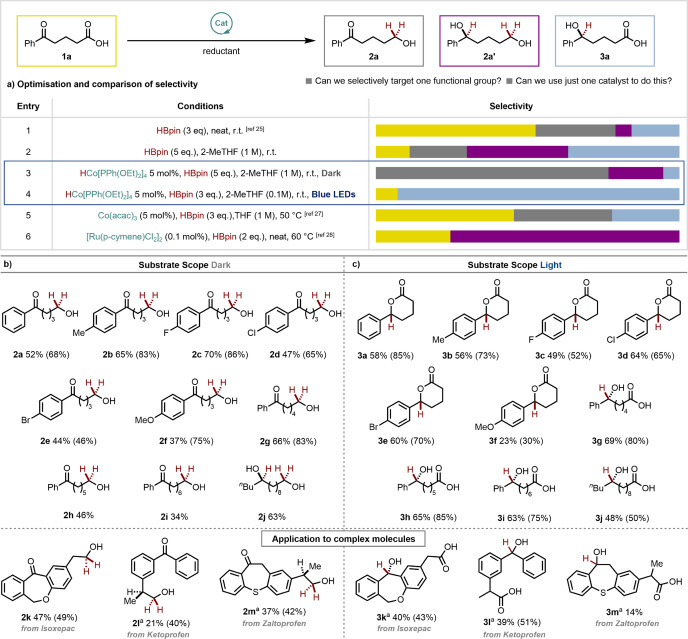
Comparison of conditions for the selective reduction of **1 a** and substrate scope of photocontrolled hydroboration. [a] 10 mol% loading of [Co]. Reactions were carried out at 0.1 mmol scale and isolated yields are reported (with yield determined by ^1^H NMR with an internal standard given in parentheses).

Based on these initial reaction conditions, we began to explore further ketoacid starting materials, with at least two potential reactive functional groups, under our optimized conditions both without and with light irradiation (Scheme 2b and c, respectively). By assessing the reactivity of substrates **1 a**–**1 e**, we were able to show that both electron‐donating and electron‐withdrawing substituents were tolerated under the reaction conditions, including useful halogen functional handles. Good selectivity was maintained for all substrates and, for the reactions under light irradiation, TFA was added to the crude reaction mixture to cyclize the products to the lactones (**3 a**–**3 f**). Notably, the conditions under light irradiation gave lower conversion for the *para*‐methoxy substituted example **1 f**, likely due to decreased electrophilicity of the ketone.

Next, we increased the chain length between the two functionalities with substrates **1 g**, **1 h** and **1 i**. Little effect was observed on the selectivity of either process, suggesting minimal intramolecular functional group assistance is at play. However, the yield of the acid reduction in the dark decreased with increasing chain length, possibly as a result of decreased solubility. On the other hand, substrates with a 1,3‐carbonyl relation were not effective in the reactions, which we attribute to possible irreversible coordination to the cobalt centre. 3‐Benzoylpropionic acid was also tested under both sets of reaction conditions but ketone reduction was the major product in both cases.^[29]^ Interestingly, when switching to a dialkyl ketone, the light reactivity profile was maintained to give product **3 j** but the reaction in the dark yielded the product from reduction of both carbonyl groups (**2 j**).

We next sought to evaluate the reactivity of diarylketone containing ketoacids. These motifs appear in a number of medicinally relevant molecules, providing a further opportunity to showcase both the mild nature of both sets of conditions, and also how our unique “on demand” selectivity could be used to diversify complex molecules. We were delighted to see that Isoxepac, Ketoprofen and Zaltoprofen all underwent photocontrolled, chemoselective hydroboration, with 10 mol% of cobalt required to obtain significant conversion in most cases. Although the yields are moderate, the selectivity is excellent with recovered starting material making up the majority of the remaining mass balance. Products **2 k** and **2 l** demonstrate that increased steric bulk at the α‐position of the carboxylic acid is tolerated and only Zaltoprofen in the light gives a low yield of **1 m** where we speculate that the sulfur might stabilise the intermediate after Co−H insertion, preventing catalyst turnover.

Finally, we were curious to explore if other functionalities could also be selectively targeted using our new approach to light controlled chemoselectivity. During the course of our previous investigations, we had observed that similar conditions, using CoH[PPh(OEt)_2_]_4_ as a catalyst with pinacolborane under blue‐light irradiation, allowed anti‐Markovnikov hydroboration of olefins.^[15a]^ Therefore, we decided to investigate the reactivity of 4‐pentenoic acid and 10‐undecenoic acid under the same conditions as optimized for the ketoacids. We were delighted to observe that the carboxylic acid moieties underwent selective hydroboration in the dark conditions, with the alkene remaining largely untouched, to yield products **2 n** and **2 o** (Scheme 3, top left). In contrast, only the alkene functionality reacts under light irradiation to give a mixture of reduced and hydroborated products **3 n**, **3 n′**, **3 o** and **3 o′** (Scheme 3, top right). Notably, when 4‐pentenoic acid is treated with pinacolborane (6 equiv) without solvent or catalyst, the sole product observed is from hydroboration of both the alkene and carboxylic acid functional groups.^[30]^

**Scheme 3:**
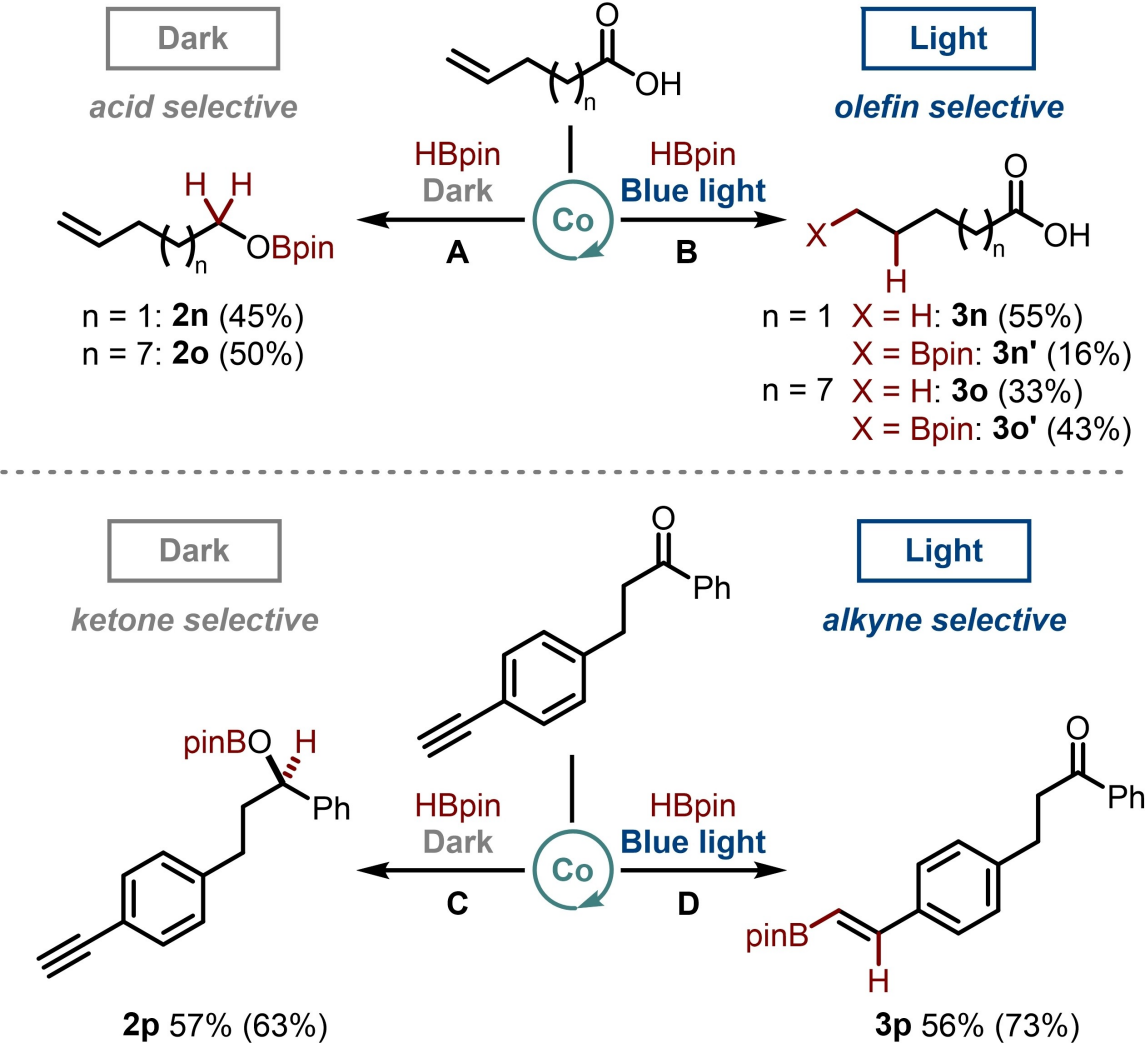
Extension to other functionalities. **A**: [Co] 5 mol%, HBpin 5.0 equiv, 2‐MeTHF (1.0 M); **B**: [Co] 5 mol%, HBpin 3.0 equiv, 2‐MeTHF (0.1 M); **C**: [Co] 5 mol%, HBpin 5.0 equiv, EtOAc (1.0 M); **D**: [Co] 1 mol%, HBpin 1.1 equiv, EtOAc (1.0 M). [Co]=CoH[PPh(OEt)_2_]_4_.

Next, we turned to the substrate that contained both an alkyne and a ketone, **1 p**, cognizant that ketones were considerably more slowly reduced than acids under dark conditions. However, we postulated that this should still be faster than alkyne reduction due to the initial coordination to the Lewis acidic boron centre. In contrast, we supposed that alkyne hydroboration may be faster under the light conditions where coordination occurs first to the unsaturated Co^I^ hydride species. This was indeed the case with **2 p** being obtained in the dark as a result of ketone hydroboration, and **3 p** as the product under light irradiation (Scheme 3, bottom). Thus, we deduce a general trend for the more polar functional group, which coordinates initially to the borane, to be selectively hydroborated in the dark.

In conclusion, we have shown a unique example of controlling chemoselectivity of a catalytic process with visible light. Taking an unselective hydroboration process, we have shown that the outcome can be controlled by a light‐responsive cobalt hydride catalyst which enables “on‐demand” chemoselective reduction of ketoacid structures, including drug molecules. Extension to unsaturated carboxylic acids, as well as alkyne‐containing substrates, was also demonstrated. More investigations to probe the scope of this concept as well as detailed mechanistic studies are currently underway in our laboratory.

## Conflict of interest

The authors declare no conflict of interests.

## Supporting information

As a service to our authors and readers, this journal provides supporting information supplied by the authors. Such materials are peer reviewed and may be re‐organized for online delivery, but are not copy‐edited or typeset. Technical support issues arising from supporting information (other than missing files) should be addressed to the authors.

Supporting Information

## Data Availability

The data that support the findings of this study are available in the Supporting Information of this article.
