# Supplementary material for: Controlling Chemoselectivity of Catalytic Hydroboration with Light
Source: Angew Chem Int Ed Engl. 2022 Jan 11;61(8):e202114482. doi: 10.1002/anie.202114482 (PMC9305532; doi:10.1002/anie.202114482)
Supplement: Supplementary file 1 — Supporting Information [file ANIE-61-0-s001.pdf]

Angewandte Chemie
Eine Zeitschrift der Gesellschaft Deutscher Chemiker

Supporting Information

Controlling Chemoselectivity of Catalytic Hydroboration with Light

*E. Bergamaschi, D. Lunic, L. A. McLean, M. Hohenadel, Y.-K. Chen, C. J. Teskey**

Table of Contents

Table of Contents 1
1. Materials and Methods 4
2. Synthesis and Characterization Data of $CoH[PPh(OEt)_2]_4$ 6
3. Selected optimization reactions 7
4. Preparation of Starting Materials 9
4.1 General procedure for the synthesis of 1,5 ketoacids: Friedel-Crafts acylation (A) 9
5-Oxo-5-(*p*-tolyl)pentanoic acid (1b) 9
5-(4-Fluorophenyl)-5-oxopentanoic acid (1c) 9
5-(4-Methoxyphenyl)-5-oxopentanoic acid (1f) 10
4.2 General procedure for the synthesis of ketoacids (B) 10
1-Phenylcyclohept-1-ene (S1) 11
(*E*)-1-Phenylcyclooct-1-ene (S2) 11
7-Oxo-7-phenylheptanoic acid (1h) 11
8-Oxo-8-phenyloctanoic acid (1i) 11
10-Oxotetradecanoic acid (1j) 12
N-methoxy-*N*-methylundec-10-enamide (1j-I) 12
1-Phenyl-3-(4-(ethynyl)phenyl)propan-1-one (1p) 14
3-(4-bromophenyl)-1-phenylpropan-1-one (1p-II) 15
5. General procedures and characterization data of reduction products 16
5.1 General procedure 1 16
5.2 General procedure 2 16
5-Hydroxy-1-phenylpentan-1-one (2a) 16
5-Hydroxy-1-(*p*-tolyl)pentan-1-one (2b) 17
1-(4-Fluorophenyl)-5-hydroxypentan-1-one (2c) 17
1-(4-Chlorophenyl)-5-hydroxypentan-1-one (2d) 17
1-(4-Bromophenyl)-5-hydroxypentan-1-one (2e) 18
5-Hydroxy-1-(4-methoxyphenyl)-1-pentanone (2f) 18
6-Hydroxy-1-phenylhexan-1-one (2g) 18
7-Hydroxy-1-phenylheptan-1-one (2h) 18
8-Hydroxy-1-phenyloctan-1-one (2i) 19
1,10-Tetradecanediol (2j) 19
2-(2-Hydroxyethyl)dibenzo[b,e]oxepin-11(6*H*)-one (2k) 19
3-(1-Hydroxypropan-2-yl)phenyl)(phenyl)methanone (2l) 19
2-(1-Hydroxypropan-2-yl)dibenzo[b,f]thiepin-10(11*H*)-one (2m) 20
n-Pent-4-enyl alcohol (2n) 20
10-Undecen-1-ol (2o) 20
3-(4-Ethynylphenyl)-1-phenylpropan-1-ol (2p) 20
6-Phenyltetrahydro-2*H*-pyran-2-one (3a) 21

6-(*p*-tolyl)tetrahydro-2*H*-pyran-2-one (3b) 21
6-(4-Fluorophenyl)tetrahydro-2*H*-pyran-2-one (3c) 21
6-(4-Chlorophenyl)tetrahydro-2*H*-pyran-2-one (3d) 22
6-(4-Bromophenyl)tetrahydro-2*H*-pyran-2-one (3e) 22
6-(4-Methoxyphenyl)tetrahydro-2*H*-pyran-2-one (3f) 22
6-Hydroxy-6-phenylhexanoic acid (3g) 22
7-Hydroxy-7-phenylheptanoic acid (3h) 23
8-Hydroxy-8-phenyloctanoic acid (3i) 23
10-Hydroxytetradecanoic acid (3j) 23
2-(11-Hydroxy-6,11-dihydrodibenzo[b,e]oxepin-2-yl)acetic acid (3k) 23
2-(3-(Hydroxy(phenyl)methyl)phenyl)propanoic acid (3l) 24
2-(10-Hydroxy-10,11-dihydrodibenzo[b,f]thiepin-2-yl)propanoic acid (3m) 24
Valeric acid (3n) and 5-(4,4,5,5-tetramethyl-1,3,2-dioxaborolan-2-yl)pentanoic acid (3n’) 24
Undecanoic acid (3o) and 5-(4,4,5,5-tetramethyl-1,3,2-dioxaborolan-2-yl)undecanoic acid (3o’) 25
(*E*)-1-Phenyl-3-(4-(2-(4,4,5,5-tetramethyl-1,3,2-dioxaborolan-2-yl)vinyl)phenyl)propan-1-one (3p) 25
6. Additional Substrates 26
6.1 5-Hexen-2-one 26
6.2 3-Benzoylpropionic acid 26
7. Comparison of selectivity 26
7.1 4-Pentenoic acid 26
8. Mechanistic Studies 27
8.1 NMR experiments 27
8.1.1 ^{1}H NMR monitoring in dark conditions 27
8.1.2 NMR monitoring in light conditions 28
8.2 Deuterium incorporation experiments 30
8.3 Experiments with TEMPO 30
8.4 The role of HBpin 31
8.5 Alternative proposed mechanism 32
9. Copies of NMR spectra 33
N-Methoxy-*N*-methylundec-10-enamide (1j-I) 33
10-Oxotetradecanoic acid (1j) 34
3-(4-Bromophenyl)-1-phenylpropan-1-one (1p-II) 35
1-Phenyl-3-(4-(ethynyl)phenyl)propan-1-one (1p) 36
5-Hydroxy-1-phenylpentan-1-one (2a) 37
5-Hydroxy-1-(p-tolyl)pentan-1-one (2b) 38
1-(4-Fluorophenyl)-5-hydroxypentan-1-one (2c) 39
1-(4-Chlorophenyl)-5-hydroxypentan-1-one (2d) 41
1-(4-Bromophenyl)-5-hydroxypentan-1-one (2e) 42
5-Hydroxy-1-(4-methoxyphenyl)-1-pentanone (2f) 43
6-Hydroxy-1-phenylhexan-1-one (2g) 44
7-Hydroxy-1-phenylheptan-1-one (2h) 45

8-Hydroxy-1-phenyloctan-1-one (2i) 46
10-Hydroxytetradecanoic acid (2j) 47
2-(2-Hydroxyethyl)dibenzo[b,e]oxepin-11(6*H*)-one (2k) 48
(3-(1-Hydroxypropan-2-yl)phenyl)(phenyl)methanone (2l) 49
2-(1-Hydroxypropan-2-yl)dibenzo[b,f]thiepin-10(11*H*)-one (2m) 50
n-Pent-4-enyl alcohol (2n) 51
10-Undecen-1-ol (2o) 52
3-(4-Ethynylphenyl)-1-phenylpropan-1-ol (2p) 53
6-Phenyltetrahydro-2*H*-pyran-2-one (3a) 54
6-(*p*-Tolyl)tetrahydro-2*H*-pyran-2-one (3b) 55
6-(4-Fluorophenyl)tetrahydro-2*H*-pyran-2-one (3c) 56
6-(4-Chlorophenyl)tetrahydro-2*H*-pyran-2-one (3d) 57
6-(4-Bromophenyl)tetrahydro-2*H*-pyran-2-one (3e) 58
6-(4-Methoxyphenyl)tetrahydro-2*H*-pyran-2-one (3f) 59
6-Hydroxy-6-phenylhexanoic acid (3g) 60
7-Hydroxy-7-phenylheptanoic acid (3h) 61
8-Hydroxy-8-phenyloctanoic acid (3i) 62
10-Hydroxytetradecanoic acid (3j) 63
2-(11-Hydroxy-6,11-dihydrodibenzo[b,e]oxepin-2-yl)acetic acid (3k) 64
2-(3-(Hydroxy(phenyl)methyl)phenyl)propanoic acid (3l) 65
2-(10-Hydroxy-10,11-dihydrodibenzo[b,f]thiepin-2-yl)propanoic acid (3m) 66
Valeric acid (3n) and 5-(4,4,5,5-tetramethyl-1,3,2-dioxaborolan-2-yl)pentanoic acid 67
(3n’) 67
Undecanoic acid (3o) and 5-(4,4,5,5-tetramethyl-1,3,2-dioxaborolan-2-yl)undecanoic acid 68
(3o’) 68
(*E*)-1-Phenyl-3-(4-(2-(4,4,5,5-tetramethyl-1,3,2-dioxaborolan-2-yl)vinyl)phenyl)propan-1-one (3p) 69
10. References 70

1. Materials and Methods

Unless otherwise stated, all reactions were performed utilizing standard Schlenk techniques. All reagents and starting materials were purchased at reagent grade and used as received. Anhydrous solvents were dried using an Innovative Technology PS-MD-5 solvent purification system. Thin layer chromatography (TLC) was performed on Merck Kieselgel 60 F254 aluminum plates with unmodified silica and visualized either under UV light or stained with potassium permanganate, vanillin, or cerium ammonium molybdate (Hanessian's stain). Column chromatography was performed with Merck silica gel 60 (35 – 70 mesh).

All ^{1}H, ^{13}C NMR spectra were recorded at ambient temperature on either Varian VNMRS 600, Varian V-NMRS 400, Bruker AV-600, Bruker AV-400 or Varian Mercury 300 spectrometers. Chemical shifts (δ/ppm) were referenced to the residual solvent peak in ^{1}H (7.26 ppm for $CDCl_3$) and ^{13}C spectra (77.16 ppm for $CDCl_3$). Coupling constants (J) are given in Hz. Signals are described as br = broad, s = singlet, d = doublet, dd = doublet of doublets, t = triplet, q = quartet, p = quintet, h = sextet and m = multiplet.

High-resolution mass spectrometry (HRMS) was performed using a Thermo Scientific LTQ Orbitrap XL spectrometer. Infrared (IR) spectra were recorded on a Perkin Elmer Spektrum 100 FT-IR spectrometer Spectrum 100 spectrometer with an UATR Diamond/KRS-5 crystal with attenuated total reflectance (ATR) and signals reported as wavenumbers in reciprocal centimeters. The ozonolysis reactions were carried out using the Fisher 502 Ozone Generator.

Some ^{1}H NMR spectra have an impurity at 4.25-4.0 ppm. This signal arises from the oxidised form of the ligand: $POPh(OEt)_2$. In some cases, when the amount was not negligible, it is stated how much it contributes to the isolated mass, and in all the cases the amount of the ligand is taken into account before calculating the isolated yields. In a lot of cases, it can be removed by preparative TLC, however due to the small scale of the reactions, it is hard to detect with UV light and so this second purification can lead to lower isolated yields.

All the photochemical reactions were carried out using a blue LED stripe (24 V, 19.2 W, λ_{max} = 467 nm) purchased from ledxon® GmbH. Reaction vials were placed approximately 2 cm from the lightsource, using a cork ring wrapped in aluminum foil as a spacer to ensure that all the vial are at the same distance from the light source (*vide infra*).

Figure S1. Experimental setup for the photochemical hydroboration of ketoacids

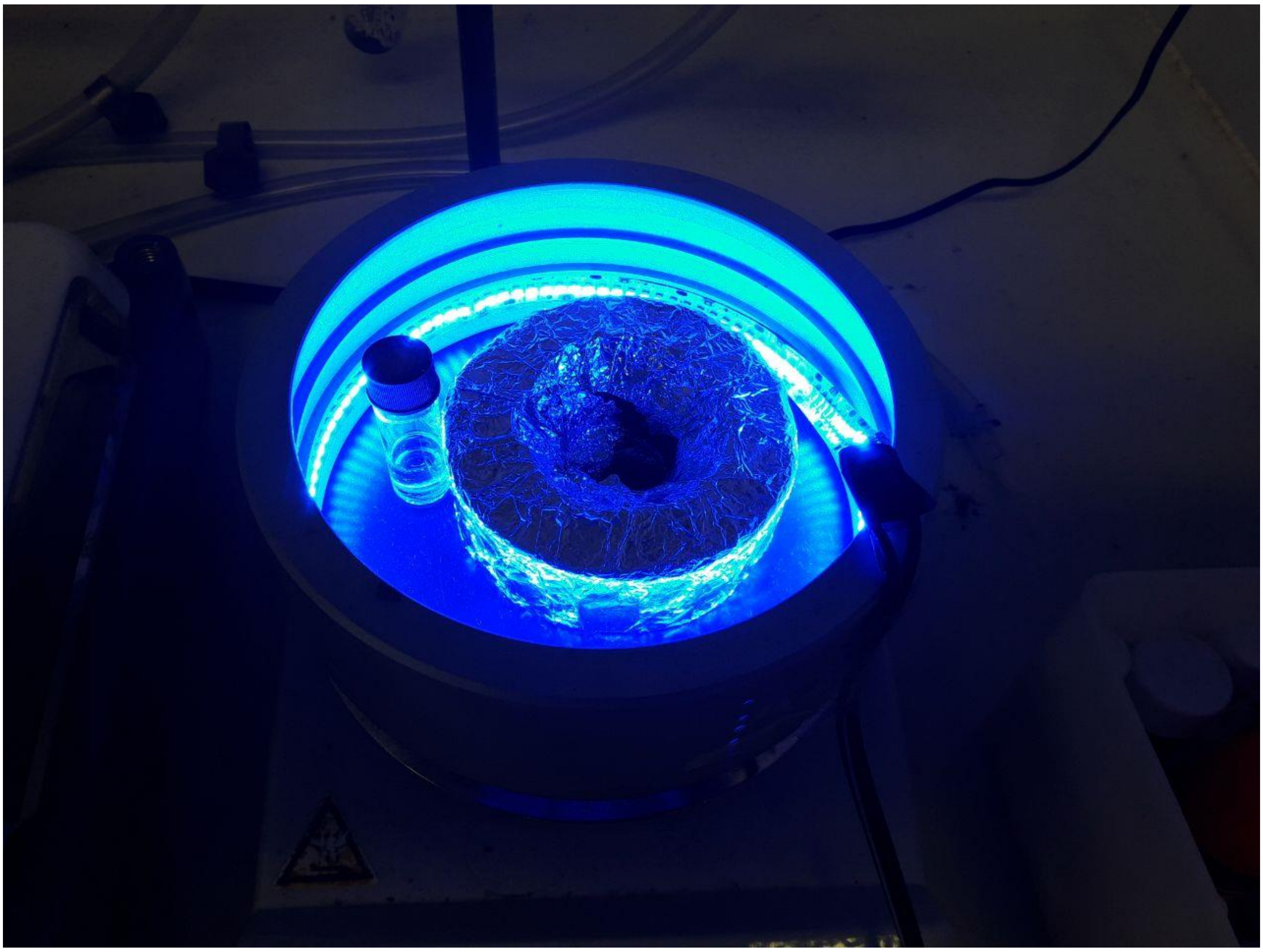

2. Synthesis and Characterization Data of CoH[PPh(OEt)$_2$]$_4$

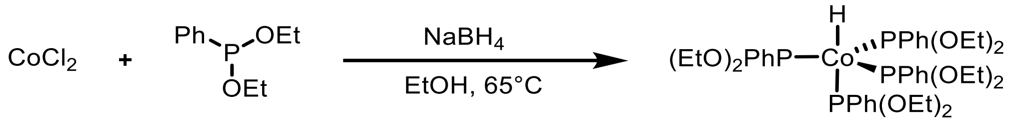

Procedure

Anhydrous $CoCl_2$ (650 mg, 5 mmol, 1 eq) was dissolved in dry ethanol (30 ml) inside an oven dried Schlenk flask under an argon atmosphere, forming a blue solution. This solution was then heated in an oil bath at 65°C. PPh(OEt)$_2$ (4.9 ml, 25 mmol, 5 eq) was added and the solution turned green. Then $NaBH_4$ (430 mg, 11 mmol, 2.2 eq) was slowly added dropwise as suspension in dry ethanol (20 ml), during the addition the solution becomes bright yellow and H_2 is produced. Once the gas evolution has stopped, the solution was filtered under argon into a clean Schlenk flask and around half of the solvent was removed under vacuum. The remaining solution was cooled to 0°C with an ice bath to induce precipitation of the product. After 20 minutes the precipitate (2.25 g, 2.65 mmol, 53%), a bright yellow solid, was filtered, dried under high vacuum, and weighed.

^{1}H NMR (400 MHz, CD_2Cl_2) δ 7.44 (s, 8H), 7.17 (s, 12H), 3.62 (s, 8H), 3.32 (s, 8H), 1.04 (s, 24H), -14.71 (q, J = 22.7 Hz, 1H). **^{13}C NMR** (101 MHz, CD_2Cl_2) δ 131.2 – 130.2 (m), 127.8, 127.1, 60.0, 16.5. **^{31}P NMR** (162 MHz, CD_2Cl_2) δ = 174.32. **IR (neat):** ν = 3056, 2971, 2927, 2887, 1480, 1436, 1384, 1099, 1024, 913, 726, 691 cm^{-1}. **HRMS (ESI):** m/z $[M+Na]^+$ calcd. for $[C_{40}H_{61}O_8P_4CoNa]^+$: 875.2547, found: 875.2544.

Figure S2. UV-Vis absorption spectra of Cobalt catalyst CoH[PPh(OEt)$_2$]$_4$

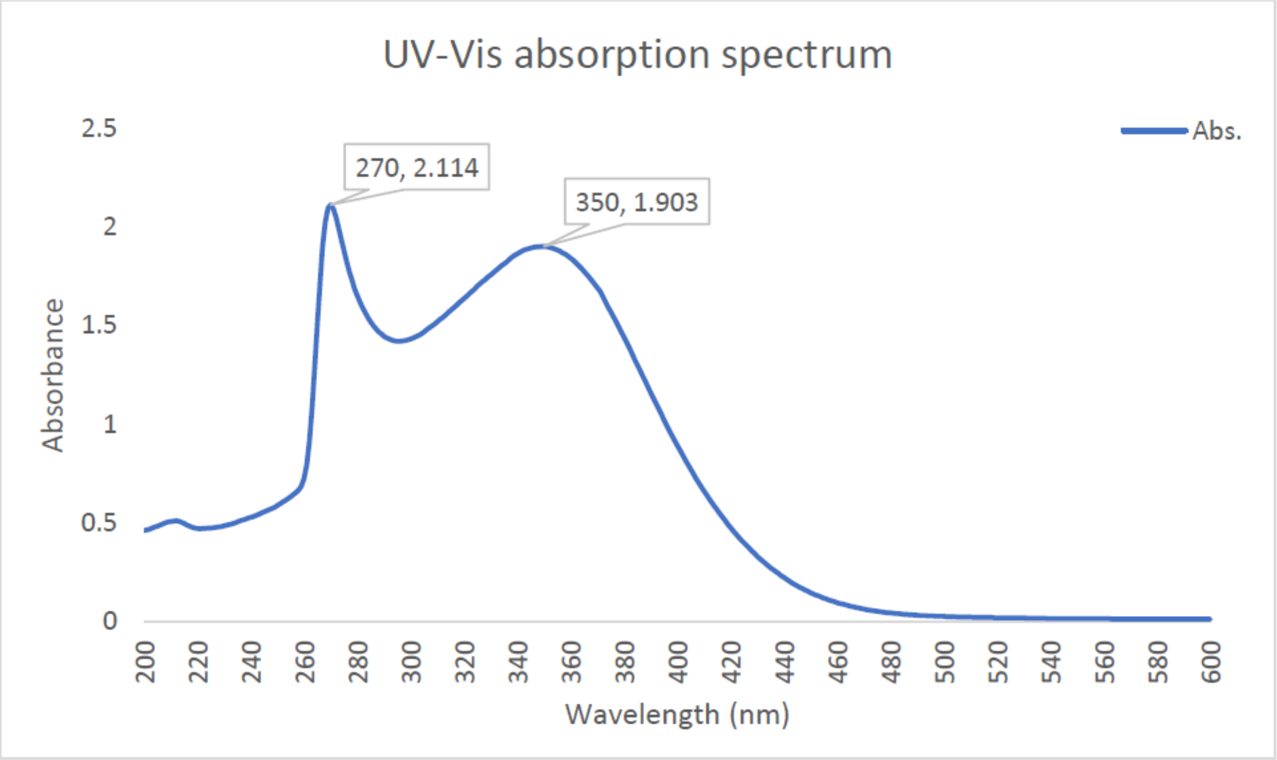

3. Selected optimization reactions

All optimization reactions were carried out on 0.1 mmol scale and analyzed by ^1H NMR spectroscopy with $CHBr_3$ as internal standard.

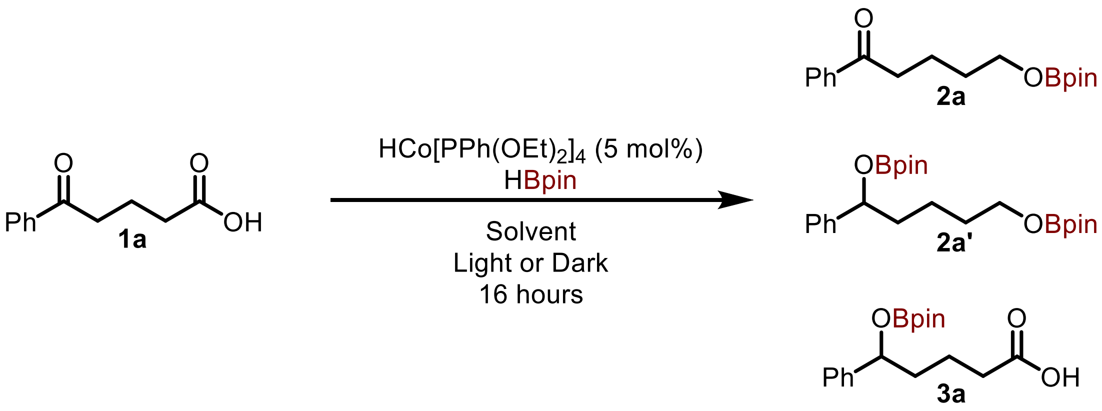

Solvent Screening

Entry	Light/Dark	Solvent (conc.)	1a recovered (%)	Yield of 2a (%)	Yield of 2a' (%)	Yield of 3a (%)
**1**	Dark	EtOAc (1.0 M)	5	52	22	7
**2**	Dark	C_6H_6 (1.0 M)	10	25	14	13
**3**	Dark	2-MeTHF (1.0 M)	-	68	17	5
**4**	Blue LEDs	EtOAc (0.1 M)	10	-	-	74
**5**	Blue LEDs	C_6H_6 (0.1 M)	10	-	-	70
**6**	Blue LEDs	2-MeTHF (0.1 M)	6	-	-	85

Entry	Light/Dark	HBpin (eq)	Solvent (conc.)	1a recovered (%)	Yield of 2a (%)	Yield of 2a' (%)	Yield of 3a (%)
**1**	Dark	2.0	2-MeTHF (1.0 M)	14	43	-	-
**2**	Dark	5.0	2-MeTHF (1.0 M)	-	68	17	5
**3**	Dark	5.0	2-MeTHF (0.1 M)	69	-	-	-
**4**	Blue LEDs	1.5	2-MeTHF (0.1 M)	40	-	-	55
**5**	Blue LEDs	3.0	2-MeTHF (0.1 M)	6	-	-	85
**6**	Blue LEDs	3.0	2-MeTHF (1.0 M)	31	-	-	50

Control reactions without cobalt catalyst

Entry	Light/Dark	Solvent (conc.)	1a recovered (%)	Yield of 2a (%)	Yield of 2a' (%)	Yield of 3a (%)
**1**	Dark	EtOAc (1.0 M)	24	16	25	20
**2**	Dark	C_6H_6 (1.0 M)	31	41	10	15
**3**	Dark	2-Me THF (1.0 M)	10	17	30	33

Raw data for the table in Scheme 2a

Entry	1a recovered (%)	Yield of 2a (%)	Yield of 2a' (%)	Yield of 3a (%)
**1**	50	25	5	15
**2**	10	17	30	33
**3**	0	72	17	5
**4**	6	0	0	78
**5**	45	32	0	22
**6**	13	0	40	0

4. Preparation of Starting Materials

4.1 General procedure for the synthesis of 1,5 ketoacids: Friedel-Crafts acylation (A)

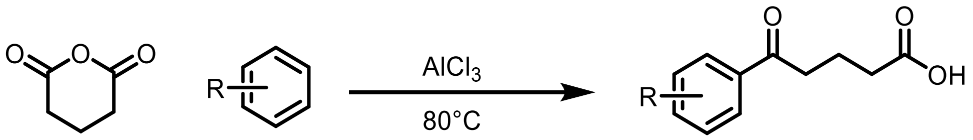

Powdered, anhydrous $AlCl_3$ (3.0 equiv, 15.0 mmol) was suspended in the appropriate substituted arene (5.0 ml) and stirred for 30 minutes. After this time glutaric anhydride (1.0 equiv, 5.0 mmol) was added and the resulting mixture was stirred overnight at 80°C. The reaction was then cooled to 0°C and quenched by slow addition of 1 M $HCl_{(aq)}$ (caution: exothermic reaction with production of fumes). The crude was diluted with EtOAc and the two layers were separated. The organic layer was basified (pH ~ 14) by addition of 10% w/w $KOH_{(aq)}$, the resulting aqueous layer was separated and acidified (pH ~ 2) with 12 M $HCl_{(aq)}$. At this point, the solution turned into a suspension, and it was extracted three times with EtOAc. The combined organic layers were washed with brine, dried ($MgSO_4$), filtered and concentrated under vacuum. The crude was purified through column chromatography (pentane:EtOAc 70:30 + 1% of AcOH).

5-Oxo-5-(*p*-tolyl)pentanoic acid (1b)

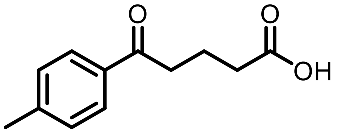

Prepared following the general procedure A starting from toluene, **1b** was obtained after purification by column chromatography and recrystallized from EtOAc as a white solid (456 mg, 2.21 mmol, 44%). **^{1}H NMR** (600 MHz, Chloroform-*d*) δ 7.86 (d, *J* = 7.9 Hz, 2H), 7.26 (d, *J* = 7.9 Hz, 2H), 3.05 (t, *J* = 7.1 Hz, 2H), 2.50 (t, *J* = 7.2 Hz, 2H), 2.41 (s, 3H), 2.12 – 2.06 (m, 2H). **^{13}C NMR** (151 MHz, Chloroform-*d*) δ 198.0, 179.0, 144.1, 134.5, 129.5, 128.3, 37.3, 32.9, 21.8, 19.3.

These data are in agreement with those reported previously in the literature.[1]

5-(4-Fluorophenyl)-5-oxopentanoic acid (1c)

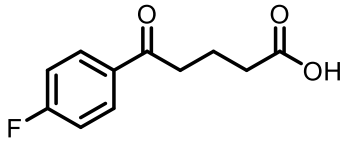

Prepared following the general procedure A starting from fluorobenzene, **1c** was obtained after purification by column chromatography and recrystallized from EtOAc as a pale-yellow solid (256 mg, 1.22 mmol, 24%). **^{1}H NMR** (600 MHz, Chloroform-*d*) δ 8.09 – 7.87 (m, 2H), 7.18 – 6.98 (m, 2H), 3.05 (t, *J* = 7.1 Hz, 2H), 2.51 (t, *J* = 7.1 Hz, 2H), 2.08 (p, *J* = 7.1 Hz, 2H). **^{13}C NMR** (151 MHz, Chloroform-*d*) δ 197.7, 178.3, 165.8 (d, *J* = 254.9 Hz), 133.2 (d, *J* = 2.9 Hz), 130.7 (d, *J* = 9.1 Hz), 115.7 (d, *J* = 21.8 Hz), 37.2, 32.8, 19.0. **^{19}F NMR** (376 MHz, Chloroform-*d*) δ -105.2.

These data are in agreement with those reported previously in the literature.[1]

5-(4-Methoxyphenyl)-5-oxopentanoic acid (1f)

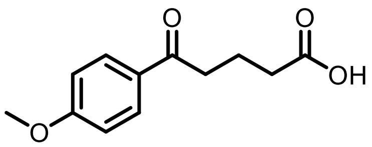

Prepared following the general procedure A starting from anisole, **1f** was obtained after purification by column chromatography as a pale brown solid (455 mg, 2.05 mmol, 41%). **^{1}H NMR** (600 MHz, Chloroform-*d*) δ 7.96 – 7.93 (d, *J* = 8.7 Hz, 2H), 6.94 (d, *J* = 8.7 Hz, 2H), 3.87 (s, 3H), 3.03 (t, *J* = 7.1 Hz, 2H), 2.50 (t, *J* = 7.1 Hz, 2H), 2.13 – 2.04 (m, 2H). **^{13}C NMR** (151 MHz, Chloroform-*d*) δ 198.1, 178.0 163.7, 130.5, 130.0, 113.9, 55.6, 37.1, 33.0, 19.4.

These data are in agreement with those reported previously in the literature.[2]

4.2 General procedure for the synthesis of ketoacids (B)

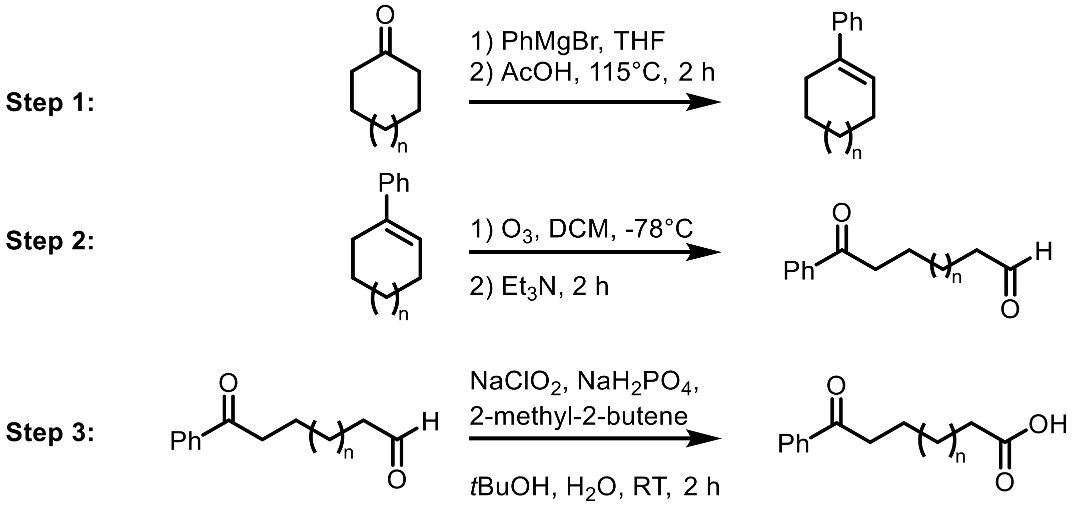

Step 1: The appropriate cyclic ketone (1.0 equiv) was dissolved in anhydrous THF (1.0 M) under an argon atmosphere and the resulting solution was cooled to -78 °C (dry ice/acetone). PhMgBr (1.2 equiv, 1 M in THF) was then added. The mixture was allowed to reach room temperature and left stirring overnight. The reaction was quenched by addition of saturated $NH_4Cl_{(aq)}$ and diluted with Et_2O. The layers were separated, and the aqueous layer was washed one more time with Et_2O. The combined organic layers were washed with brine, dried ($MgSO_4$), filtered and concentrated under vacuum. The resulting crude was directly dissolved in AcOH (25 ml) and refluxed (115°C) in an oil bath for 2 hours. The reaction was quenched by slow addition of saturated $NaHCO_{3(aq)}$. Once the CO_2 evolution has stopped, Et_2O was added, and the layers were separated. The organic layer was subsequently washed with water and brine, dried ($MgSO_4$), filtered and concentrated under vacuum.

Step 2: The alkene prepared in the previous step was dissolved in DCM in a Schlenk tube capped with a gas bubbler. The solution was flushed with argon for 5 minutes and then cooled to -78°C. Ozone was then bubbled in the solution until a pale blue color appeared. The ozone stream was then interrupted, and the solution was flushed with argon for 30 minutes and allowed to reach room temperature. Et_3N (2.0 equiv) was added, and the solution turned yellow. After two hours of stirring the crude was washed twice with 1 M $HCl_{(aq)}$

Step 3: The obtained crude aldehyde was dissolved in water (20 equiv) and *tert*-butyl alcohol (52 equiv). To this was added 2-methyl-2-butene (10 equiv), $NaClO_2$ (3.0 equiv) and NaH_2PO_4 (5.0 equiv). The mixture was left to stir during 2h at room temperature. Then, EtOAc was added to the mixture, and extraction was done

3 times. The combined organic layers were washed with brine, dried ($MgSO_4$), filtered and concentrated under vacuum.

1-Phenylcyclohept-1-ene (S1)

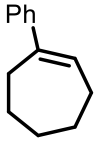

Prepared following the general procedure B (step 1) starting from cycloheptanone (5.5 mmol), **S1** was obtained after purification by column chromatography (pentane) as a colorless liquid (430 mg, 2.5 mmol, 46%). **^{1}H NMR** (600 MHz, Chloroform-*d*) δ 7.34 – 7.27 (m, 4H), 7.20 (td, *J* = 6.9, 1.6 Hz, 1H), 6.10 (td, *J* = 6.8, 2.2 Hz, 1H), 2.80 – 2.45 (m, 2H), 2.33 – 2.26 (m, 2H), 1.88 – 1.81 (m, 2H), 1.70 – 1.62 (m, 2H), 1.60 – 1.54 (m, 2H). **^{13}C NMR** (151 MHz, Chloroform-*d*) δ 145.1, 145.0, 130.4, 128.1, 126.2, 125.7, 32.8, 32.8, 28.9, 26.9, 26.8.

These data are in agreement with those reported previously in the literature.[3]

(*E*)-1-Phenylcyclooct-1-ene (S2)

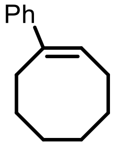

Prepared following the general procedure B (step 1) starting from cyclooctanone (5.5 mmol), **S2** was obtained after purification by column chromatography (pentane) as a colorless liquid (652 mg, 3.5 mmol, 64%). **^{1}H NMR** (600 MHz, Chloroform-*d*) δ 7.41 (m, 2H), 7.30 (m, 2H), 7.23 – 7.19 (m, 1H), 6.01 (t, *J* = 8.3 Hz, 1H), 2.66 – 2.61 (m, 2H), 2.33 – 2.25 (m, 2H), 1.68 – 1.57 (m, 4H), 1.54 (dd, *J* = 6.0, 2.9 Hz, 4H). **^{13}C NMR** (151 MHz, Chloroform-*d*) δ 143.2, 140.2, 128.2, 128.0, 126.4, 125.8, 30.0, 29.5, 28.5, 27.4, 26.9, 26.2.

These data are in agreement with those reported previously in the literature.[4]

7-Oxo-7-phenylheptanoic acid (1h)

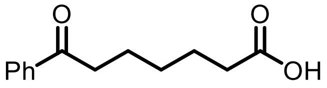

Prepared following the general procedure B (step 3) starting from **S1**, **1h** was obtained after purification by column chromatography (EtOAc: pentane 10:90 +1% AcOH) as a white solid (372 mg, 1.69 mmol, 68%). **^{1}H NMR** (600 MHz, Chloroform-*d*) *δ* 7.95 (d, *J* = 7.4 Hz, 2H), 7.56 (t, *J* = 7.3 Hz, 1H), 7.46 (t, *J* = 7.7 Hz, 2H), 2.99 (t, *J* = 7.3 Hz, 2H), 2.39 (t, *J* = 7.5 Hz, 2H), 1.78 (p, *J* = 7.5 Hz, 2H), 1.71 (p, *J* = 7.5, 2H), 1.49 – 1.43 (m, 2H). **^{13}C NMR** (151 MHz, Chloroform-*d*) *δ* 200.3, 179.4, 137.1, 133.1, 128.7, 128.2, 38.4, 33.9, 28.9, 24.7, 24.0.

These data are in agreement with those reported previously in the literature.[5]

8-Oxo-8-phenyloctanoic acid (1i)

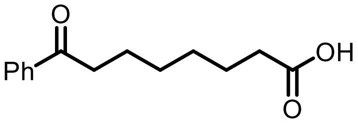

Prepared following the general procedure B (step 3) starting from **S2**, **1i** was obtained after purification by column chromatography (EtOAc: pentane 10:90 +1% AcOH) as a white solid (538 mg, 2.3 mmol, 66%). **^{1}H NMR** (600 MHz, Chloroform-*d*) *δ* 7.98 – 7.92 (m, 2H), 7.58 – 7.53 (m, 1H), 7.46 (t, *J* = 7.8 Hz, 2H), 2.97 (t, *J* = 7.3 Hz, 2H), 2.36 (t, *J* = 7.5 Hz, 2H), 1.79 – 1.61 (m, 4H), 1.41 (p, *J* = 3.6 Hz, 4H). **^{13}C NMR** (151 MHz, Chloroform-*d*) *δ* 200.4, 178.9, 137.1, 132.9, 128.6, 128.0, 38.4, 33.8, 28.9, 28.9, 24.5, 24.1.

These data are in agreement with those reported previously in the literature.[6]

10-Oxotetradecanoic acid (1j)

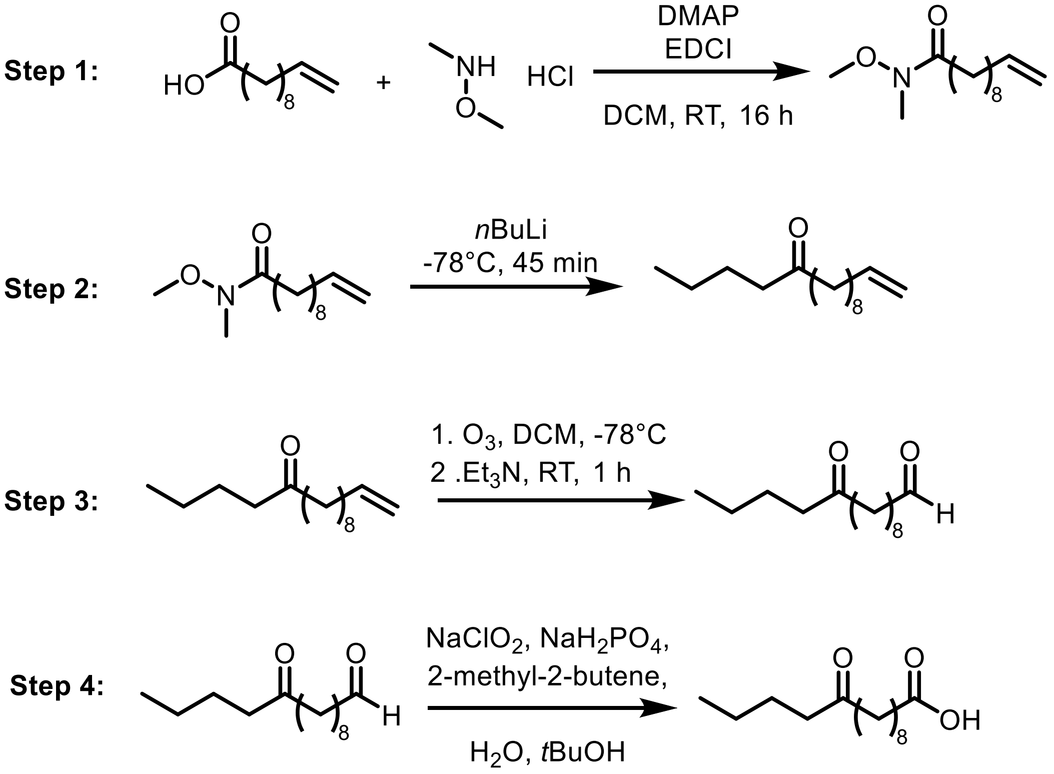

***N*-methoxy-*N*-methylundec-10-enamide (1j-I)**

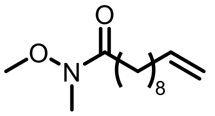

Step 1: To a solution of 10-undecenoic acid (1.0 equiv, 10 mmol, 1.84 g) in DCM (40 mL) was added EDCI (1.5 equiv, 15 mmol, 2.87 g), DMAP (1.5 equiv, 15 mmol, 1.83 g) and *N*,*O*-dimethylhydroxyamine hydrochloride (1.5 eq, 15 mmol, 1.46 g). The resulting mixture was stirred at room temperature for 16 h before being quenched with brine. The phases were separated, and the aqueous phase was extracted with DCM. The combined organic phases were washed with 2M $HCl_{(aq)}$ and brine before being dried ($MgSO_4$) and concentrated under vacuum giving a pale-yellow oil. The desired amide was obtained clean. The isolated yield is 73% (1.63 g, 7.3 mmol).**^{1}H NMR** (600 MHz, Chloroform-*d*) δ 5.85 – 5.75 (m, 1H), 4.98 (dd, *J* = 17.2, 1.8 Hz, 1H), 4.92 (dt, *J* = 10.2, 2.4, 1.3 Hz, 1H), 3.67 (s, 3H), 3.17 (s, 3H), 2.40 (d, *J* = 7.7 Hz, 2H), 2.05 – 2.00 (m, 2H), 1.65 – 1.59 (m, 2H), 1.38 – 1.26 (m, 10H).

These data are in agreement with those previously reported in the literature.[7]

Step 2: The Weinreb amide (1.0 equiv, 1.63 g, 7.3 mmol) was dissolved in THF (25 mL) under argon atmosphere. *n*BuLi (1.3 equiv, 9.5 mmol) was added to this solution at -78°C (acetone/dry ice). The mixture was left to stir at the same temperature for 45 minutes. The reaction mixture was warmed to room temperature and was then quenched with saturated $NH_4Cl_{(aq)}$. The crude was extracted with Et_2O, the organic layers were dried ($MgSO_4$), filtered and the solvent was removed under vacuum. The desired product was used without further purification.

Step 3: The obtained product was dissolved in DCM and treated with ozone at -78°C (acetone/dry ice) until the appearance of a pale blue color. When the reaction was finished, the mixture was flushed with argon for 30 minutes, and then Et_3N (2 equiv, 14.6 mmol) was added to the mixture at RT. The solution became yellow. It was left to stir for 1 hour. The mixture was then diluted with DCM and washed twice with water, the organic layer was dried ($MgSO_4$), filtered and the solvent was removed under vacuum. The obtained aldehyde was used in the next step without further purification.

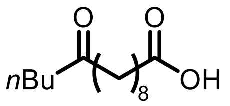

Step 4: The aldehyde was dissolved in water (20 equiv) and *tert*-butyl alcohol (52 equiv). To this was added 2-methyl-2-butene (10 equiv), $NaClO_2$ (3.0 equiv) and NaH_2PO_4 (5.0 equiv). The mixture was left to stir for 2 hours at room temperature. The mixture was then diluted with EtOAc, and it was washed three times with water. The combined organic layers were washed with brine, dried ($MgSO_4$), filtered and concentrated under vacuum. The desired product was purified by column chromatography (DCM: AcOH= 99:1) and obtained as a white solid in 22% yield (390.9 mg, 1.61 mmol)

^{1}H NMR (600 MHz, Chloroform-*d*) δ 2.38 (td, *J* = 7.5, 2.6 Hz, 4H), 2.34 (t, *J* = 7.5 Hz, 2H), 1.63 (m, 2H), 1.58 – 1.51 (m, 4H), 1.36 – 1.24 (m, 10H), 0.90 (t, *J* = 7.3 Hz, 3H). **^{13}C NMR** (151 MHz, Chloroform-*d*) δ 211.9, 42.9, 42.7, 33.8, 29.3, 29.2, 29.1, 26.1, 24.8, 24.0, 22.5, 14.0. **IR (neat):** ν 3056, 2925, 2853, 1694, 1418, 1291, 1225, 876, 680 cm^{-1}. **HRMS (ESI):** m/z $[M+Na]^+$ calculated for $[C_{14}H_{26}O_3Na]^+$: 265.1774, found: 265.1771.

These data are in agreement with those previously reported in the literature.[8]

1-Phenyl-3-(4-(ethynyl)phenyl)propan-1-one (1p)

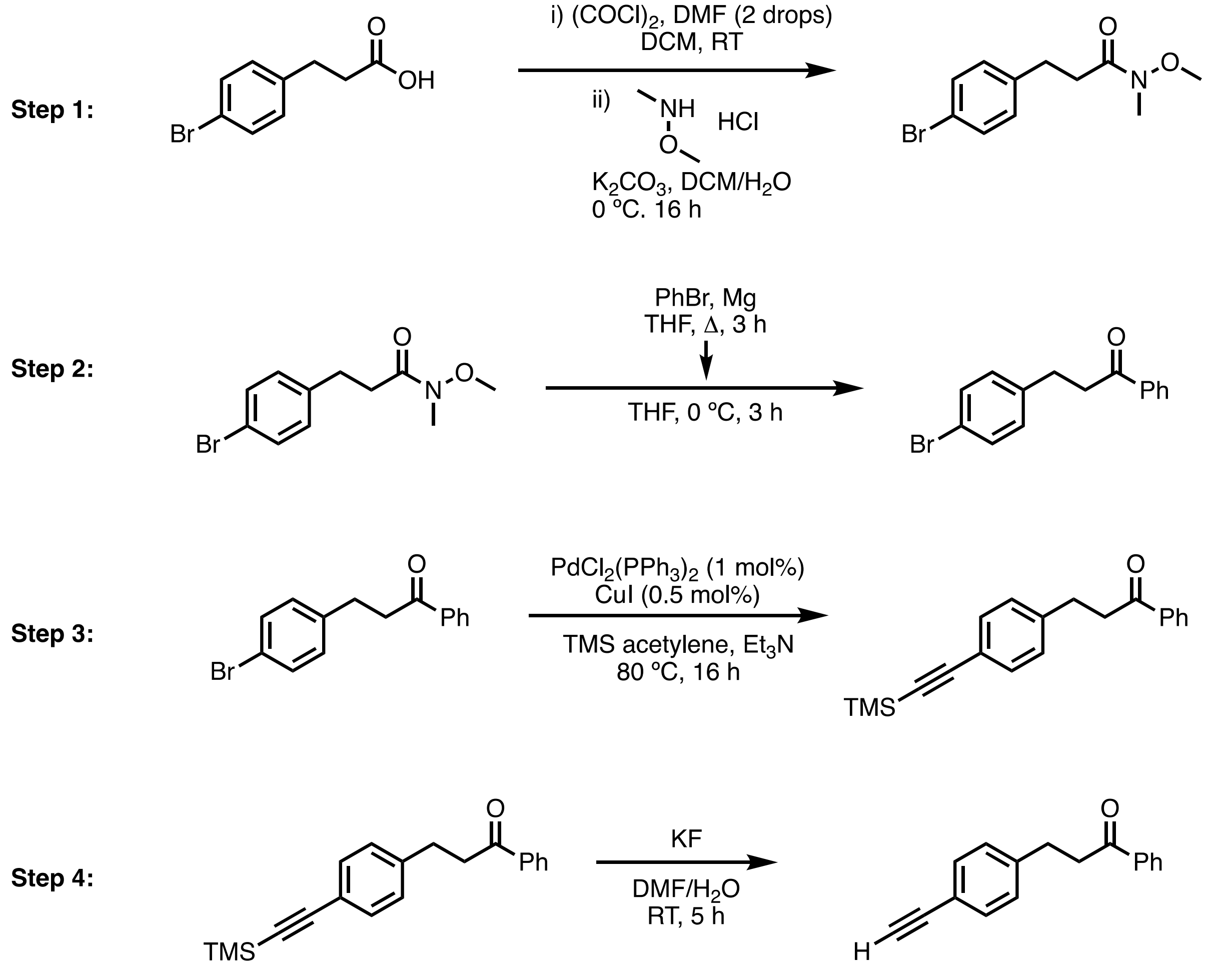

Step 1: To a 50 mL round-bottom flask, equipped with a stir-bar, was added 3-(4-bromophenyl)propanoic acid (1.00 equiv, 8.00 mmol, 1.83 g), DCM (10 mL) and DMF (2 drops). The mixture was then allowed to stir at RT and oxalyl chloride (1.10 equiv, 8.80 mmol, 0.76 mL) was added dropwise and stirring was continued until effervescence had ceased and acid chloride formation was assumed. To another 50 mL round-bottom flask, equipped with a stir-bar, was added *N*,*O*-dimethylhydroxylamine hydrochloride (1.10 equiv, 8.80 mmol, 1.12 g) and H_2O (2 mL). The mixture was cooled to 0 ºC and stirred vigorously and then the acid chloride solution was added followed by portionwise addition of K_2CO_3 (1.15 equiv, 9.20 mmol, 1.27 g). The reaction mixture was then warmed to RT and stirred for 16 h. The reaction mixture was then diluted with DCM (20 mL) and H_2O (20 mL) and the phases were separated. The aqueous phase was further extracted with DCM (2 x 20 mL). The organic phases were combined, washed with sat. $NaHCO_{3(aq)}$ (30 mL), 2 M $HCl_{(aq)}$ (30 mL), brine (30 mL). The organic phase was dried ($MgSO_4$), filtered and concentrated under vacuum. The desired product was taken on without further purification as a white solid in 76% yield (1.65 g, 6.10 mmol).

Step 2: To an oven-dried Schlenk-flask, equipped with a stir-bar, was added Mg turnings (1.00 equiv, 5.51 mmol, 134 mg) and THF (10 mL). The Schlenk tube was then purged with argon for 20 mins and stirred at RT. Bromobenezene (1.10 equiv, 6.1 mmol, 0.63 mL) was added dropwise. Upon complete addition of bromobenzene the flask was then heated to reflux for 3 h. The reaction mixture was then cooled to RT and Grignard reagent formation was assumed. To another oven-dried Schlenk-flask, equipped with a stir-bar, was

added 3-(4-bromophenyl)-*N*-methoxy-*N*-methylpropanamide (1.00, 5.51 mmol, 1.50 g) and THF (10 mL). The resulting mixture was stirred and cooled to 0 ºC and then, the Grignard reagent solution was added dropwise to give a yellow mixture, which was allowed to stir for 3 h. The reaction mixture was quenched by the addition of sat.$NH_4Cl_{(aq)}$ (20 mL) and extracted with Et_2O (3 x 20 mL). The organic phase was dried ($MgSO_4$), filtered and concentrated under vacuum. The desired product (**1p-II**) was purified with column chromatography (Pentane: EtOAc= 90:10) and obtained as a white solid in 34% yield (542 mg, 1.87 mmol).

3-(4-bromophenyl)-1-phenylpropan-1-one (1p-II)

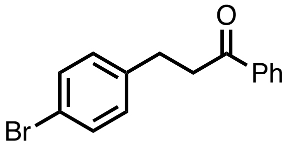

^{1}H NMR (600 MHz, Chloroform-*d*) δ 7.99 – 7.90 (m, 2H), 7.56 (tt, *J* = 7.3, 1.2 Hz, 1H), 7.48 – 7.44 (m, 2H), 7.41 (dt, *J* = 8.4, 1.9 Hz, 2H), 7.13 (dt, *J* = 8.4, 4.9 Hz, 2H), 3.28 (t, *J* = 7.3 Hz, 2H), 3.03 (t, *J* = 7.5 Hz, 2H). **^{13}C NMR** (151 MHz, Chloroform-*d*) δ 198.8, 140.3, 136.7, 133.2, 131.6, 130.2, 128.6, 128.0, 119.9, 40.1, 29.4. **IR (neat):** ν 3033, 2925, 2879, 2325, 1903, 1665, 1589, 821, 770, 684 cm^{-1}. **HRMS (ESI):** m/z $[M+H]^+$ calculated for $[C_{15}H_{14}OBr]^+$: 289.0223, found: 289.0220, 291.0200.

Step 3: To an oven-dried Schlenk-flask, equipped with a stir-bar, was added $PdCl_2(PPh_3)_2$ (0.01 equiv, 13.5 mmol, 9.50 mg), CuI (0.005 equiv, 6.8 mmol, 1.30 mg) and 3-(4-bromophenyl)-1-phenylpropan-1-one (1.00 equiv, 1.35 mmol, 390 mg) and the flask was evacuated and backfilled with argon (3x). Et_3N (4.05 mL) and TMS acetylene (1.20 equiv, 1.62 mmol, 0.23 mL) was added and the reaction mixture was heated to 80 ºC for 16 h. The reaction mixture was then cooled to RT and quenched by the addition of 1 M $HCl_{(aq)}$ (60 mL) and extracted with Et_2O (3 x 30 mL). The organic phase was dried ($MgSO_4$), filtered and concentrated under vacuum. The crude product was obtained with column chromatography (Pentane: Et_2O= 95:5) and was taken on without further purification.

Step 4: To a 10 mL round-bottom flask, equipped with a stir-bar, was added the crude 1-phenyl-3-(4-((trimethylsilyl)ethynyl)phenyl)propan-1-one (1.00 equiv, 0.60 mmol, 185 mg), KF (5.00 equiv, 3.00 mmol, 174 mg), DMF (3 mL) and H_2O (0.3 mL). The reaction was then stirred at RT for 5 h. The reaction mixture was then diluted with H_2O (3 mL) and Et_2O (10 mL) and the phases separated. The organic phase was washed with H_2O (3 x 10 mL). The organic phase was dried ($MgSO_4$), filtered and concentrated under vacuum. The desired product was purified with column chromatography (Pentane: EtOAc= 90:10) and obtained as a white solid in 22% yield over two steps (71.1 mg, 0.30 mmol).

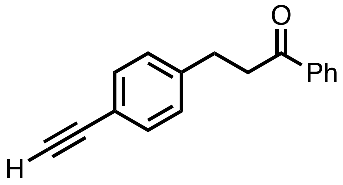

^{1}H NMR (600 MHz, Chloroform-*d*) δ 7.95 (d, *J* = 7.6 Hz, 2H), 7.56 (t, *J* = 7.5 Hz, 1H), 7.49 – 7.37 (m, 4H), 7.21 (d, *J* = 7.7 Hz, 2H), 3.29 (t, *J* = 7.8 Hz, 2H), 3.08 (t, *J* = 7.6 Hz, 2H), 3.04 (d, *J* = 1.8 Hz, 1H). **^{13}C NMR** (151 MHz, Chloroform-*d*) δ 198.8, 142.3, 136.7, 133.1, 132.3, 128.6, 128.5, 128.0, 119.9, 83.6, 40.0, 29.9. **IR (neat):** ν 3287, 3059, 2928, 2325, 2106, 1682, 1597 cm^{-1}. **HRMS (ESI):** m/z $[M+Na]^+$ calculated for $[C_{17}H_{14}ONa]^+$: 257.0937, found: 257.0935.

5. General procedures and characterization data of reduction products

5.1 General procedure 1

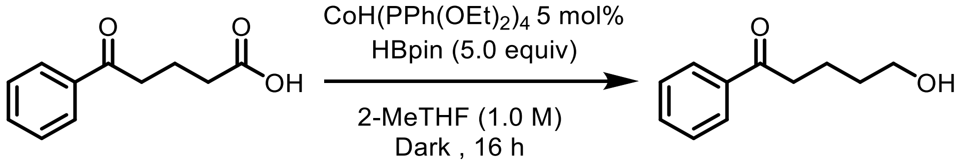

In an oven-dried 4 ml vial were introduced the cobalt catalyst (5 mol%, 10 µmol, 8.6 mg), the starting material (1.0 equiv, 0.2 mmol) and a stirring bar. A cap with rubber septum was used to close the vial and the system was then purged with argon. 2-MeTHF (C = 1.0 M, 0.2 mL), HBpin (5.0 equiv, 1.0 mmol, 144 µL) were then added successively and the vial was placed in the dark overnight. After this time, the solution was diluted with Et_2O and washed two times with distilled water, the combined aqueous layers were extracted two times with Et_2O and the combined organic layers were washed with brine, dried ($MgSO_4$), filtered and concentrated under vacuum. The resulting crude was then purified by column chromatography as detailed for the single compounds.

5.2 General procedure 2

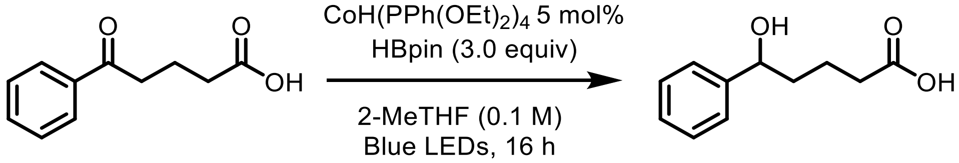

In an oven-dried 4 ml vial were introduced the cobalt catalyst (5 mol%, 10 µmol, 8.6 mg), the starting material (1.0 equiv, 0.2 mmol) when solid and a stirring bar. A cap with rubber septum was used to close the vial and the system was then purged with argon. 2-MeTHF (C = 0.1 M, 2.0 mL), HBpin (3.0 equiv, 0.6 mmol, 86 µL) were then added successively and the vial was exposed to blue LEDs overnight. After this time, the solution was diluted with Et_2O and washed two times with distilled water, the combined aqueous layers were extracted two times with Et_2O and the combined organic layers were washed with brine, dried ($MgSO_4$), filtered and concentrated under vacuum.

Variation for 1,5 ketoacids **1a-1f**:

After stirring overnight under irradiation, TFA (5.0 equiv, 1.0 mmol, 77 µL) was added and the mixture was stirred for two hours to induce the lactonization of the products. The solvent was then removed under reduced pressure. The resulting crude was then purified by column chromatography as detailed for the single compounds.

5-Hydroxy-1-phenylpentan-1-one (2a)

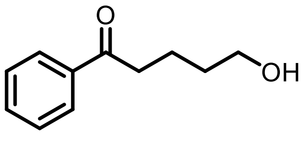

Prepared following general procedure 1, **2a** was obtained after purification by column chromatography (DCM: acetone 95:5) as a colorless oil (18.6 mg, 104 µmol, 52%). The isolated sample contains 14% of oxidized ligand, the

corresponding mass has been subtracted before calculating the yield. R_f = 0.42 (DCM: acetone 90:10). **^{1}H NMR** (600 MHz, Chloroform-*d*) *δ* 8.00 – 7.93 (m, 2H), 7.58 – 7.54 (m, 1H), 7.49 – 7.45 (m, 2H), 3.68 (t, *J* = 6.4 Hz, 2H), 3.03 (t, *J* = 7.1 Hz, 2H), 1.89 – 1.81 (m, 2H), 1.70 – 1.65 (m, 2H). **^{13}C NMR** (151 MHz, Chloroform-*d*) *δ* 200.5, 137.1, 133.2, 128.7, 128.2, 62.6, 38.3, 32.4, 20.3. **IR (neat):** ν 3412, 2937, 2872, 1681, 1596, 1447, 1231, 1022, 698, 753, 693 cm^{-1}. **HRMS (ESI):** m/z [M+Na]$^+$ calcd. for $[C_{11}H_{14}O_2Na]^+$: 201.0886, found: 201.0882.

These data are in agreement with those reported previously in the literature.[9]

5-Hydroxy-1-(*p*-tolyl)pentan-1-one (2b)

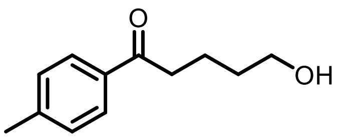

Prepared following general procedure 1, **2b** was obtained after purification by column chromatography (DCM: acetone 95:5), followed by preparative TLC (DCM: acetone 90:10) as a colorless oil (24.9 mg, 130 µmol, 65%). R_f = 0.31 (DCM: acetone 90:10). **^{1}H NMR** (600 MHz, Chloroform-*d*) δ 7.86 (d, *J* = 8.0 Hz, 2H), 7.25 (d, *J* = 8.4 Hz, 2H), 3.67 (t, *J* = 6.4 Hz, 2H), 3.00 (t, *J* = 7.1 Hz, 2H), 2.41 (s, 3H), 1.84 (p, *J* = 7.2 Hz, 2H), 1.72 – 1.59 (m, 2H). ^{13}C NMR (151 MHz, Chloroform-*d*) δ 200.2, 143.9, 134.6, 129.4, 128.3, 62.5, 38.1, 32.4, 21.8, 20.4. **IR (neat):** ν 3413, 2935, 2871, 1676, 1606, 1232, 1021, 969, 806 cm^{-1}. **HRMS (ESI)**: m/z [M+Na]$^+$ calcd. for $[C_{12}H_{16}O_2Na]^+$: 215.1048, found: 215.1053

These data are in agreement with those reported previously in the literature.[9]

1-(4-Fluorophenyl)-5-hydroxypentan-1-one (2c)

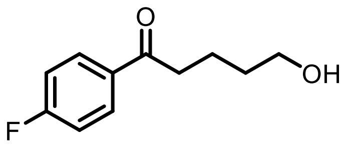

Prepared following general procedure 1, **2c** was obtained as a 1:1 mixture with pinacol after purification by column chromatography (EtOAc: pentane 30:70) as a colorless oil (27.3 mg, 140 µmol, 70%). R_f = 0.25 (EtOAc: pentane 30:70).

^{1}H NMR (600 MHz, Chloroform-*d*) δ 8.02 – 7.96 (m, 2H), 7.13 (t, *J* = 8.6 Hz, 2H), 3.68 (t, *J* = 6.4 Hz, 2H), 3.00 (t, *J* = 7.1 Hz, 2H), 1.84 (p, *J* = 7.2 Hz, 2H), 1.69 – 1.63 (m, 2H). **^{13}C NMR** (151 MHz, Chloroform-*d*) δ 198.7, 165.7 (d, $^{1}J_{CF}$ = 254.4 Hz), 133.4 (d, $^{4}J_{CF}$ = 3.1 Hz), 130.7 (d, $^{3}J_{CF}$ = 9.2 Hz), 115.7 (d, $^{2}J_{CF}$ = 21.8 Hz), 62.4, 38.0, 32.2, 20.2. **^{19}F NMR** (565 MHz, Chloroform-*d*) δ –105.4. **IR (neat):** ν 3271, 2938, 2876, 1733, 1675, 1596, 1506, 1409, 1373, 1231, 1155, 1048, 822, 735 cm^{-1}. **HRMS (ESI)**: m/z [M+Na]$^+$ calcd. for $[C_{11}H_{13}O_2FNa]^+$: 219.07918, found: 219.07903.

1-(4-Chlorophenyl)-5-hydroxypentan-1-one (2d)

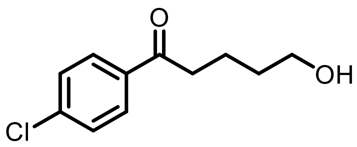

Prepared following general procedure 1, **2d** was obtained after purification by column chromatography (DCM: acetone 95:5), followed by preparative TLC (DCM: acetone 90:10) as a colorless oil (20.0 mg, 94 µmol, 47%). R_f =0.40 (DCM: acetone 90:10). **^{1}H NMR** (600 MHz, Chloroform-*d*) δ 7.89 (d, *J* = 8.6 Hz, 2H), 7.42 (d, *J* = 8.5 Hz, 2H), 3.67 (t, *J* = 6.3 Hz, 2H), 2.99 (t, *J* = 7.1 Hz, 2H), 1.83 (p, *J* = 7.2 Hz, 2H), 1.68 – 1.60 (m, 2H). **^{13}C NMR** (151 MHz, Chloroform-*d*) δ 199.1, 139.5, 135.2, 129.5, 128.9, 62.4, 38.1, 32.2, 20.2. **IR (neat):** ν 2961, 1685, 1258, 1014, 793 cm^{-1}. **HRMS (ESI)**: m/z [M+Na]$^+$ calcd. for $[C_{11}H_{13}O_2ClNa]^+$: 235.0502, found: 235.0508.

These data are in agreement with those reported previously in the literature.[9]

1-(4-Bromophenyl)-5-hydroxypentan-1-one (2e)

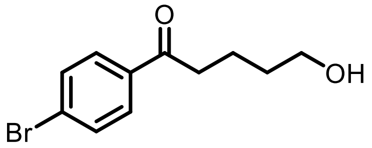

Prepared following general procedure 1, **2e** was obtained after purification by column chromatography (DCM: acetone = 98:2) as pale-yellow solid (22.4 mg, 87 µmol, 44%). The isolated sample contains 15% of oxidized ligand, the corresponding mass has been subtracted before calculating the yield. R_f = 0.33 (DCM: acetone = 95:5). **^{1}H NMR** (600 MHz, Chloroform-*d*) δ 7.75 (d, *J* = 8.5 Hz, 2H), 7.53 (d, *J* = 8.5 Hz, 2H), 3.60 (t, *J* = 6.4 Hz, 2H), 2.92 (t, *J* = 7.1 Hz, 2H), 1.79 – 1.73 (m, 2H), 1.64 – 1.55 (m, 2H)., **^{13}C NMR** (151 MHz, Chloroform-*d*) δ 198.2, 134.6, 130.9, 128.6, 127.2, 61.3, 37.1, 31.1, 19.2. **IR (neat):** ν 3291, 2932, 2867, 1676, 1583, 1396, 1256, 1061, 1013, 797, 743. cm^{-1}. **HRMS (APCI):** m/z $[M+H]^+$ calculated for $[C_{11}H_{14}O_2Br]^+$: 257.0165, found: 257.0172.

5-Hydroxy-1-(4-methoxyphenyl)-1-pentanone (2f)

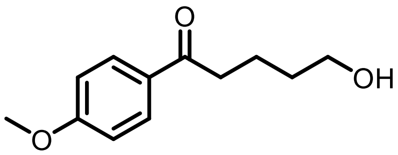

Prepared following general procedure 1, **2f** was obtained after purification by column chromatography (DCM: acetone 90:10), followed by preparative TLC (DCM: acetone 70:30) as a colorless oil (15.4 mg, 74 µmol, 37%). R_f =0.30 (DCM: acetone 90:10). **^{1}H NMR** (600 MHz, Chloroform-*d*) δ 7.98 – 7.92 (m, 2H), 6.95 – 6.92 (m, 2H), 3.87 (s, 3H), 3.67 (t, *J* = 6.4 Hz, 2H), 2.97 (t, *J* = 7.1 Hz, 2H), 1.83 (dd, *J* = 8.6, 6.4 Hz, 2H), 1.65 (dd, *J* = 8.6, 6.4 Hz, 2H). **^{13}C NMR** (151 MHz, Chloroform-*d*) δ 199.1, 163.6, 130.5, 113.9, 62.5, 55.6, 37.9, 32.4, 20.5. **IR (neat):** ν 3418, 2934, 2870, 1725, 1669, 1598, 1253, 1170, 1027, 976, 818 cm^{-1}. **HRMS (ESI):** m/z $[M+Na]^+$ calculated for $[C_{12}H_{16}O_3Na]^+$: 231.0992, found: 231.0987.

These data are in agreement with those reported previously in the literature.[9]

6-Hydroxy-1-phenylhexan-1-one (2g)

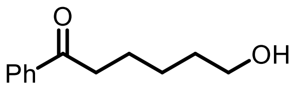

Prepared following general procedure 1, **2g** was obtained after purification by column chromatography (DCM: acetone 98:2) as a colorless oil (25.3 mg, 132 µmol, 66%). R_f = 0.40 (DCM: acetone 98:2). **^{1}H NMR** (600 MHz, Chloroform-*d*) *δ* 7.99 – 7.93 (m, 2H), 7.57 – 7.54 (m, 1H), 7.48 – 7.44 (m, 2H), 3.68 (t, *J* = 6.5 Hz, 2H), 2.99 (t, *J* = 7.3 Hz, 2H), 1.82 – 1.75 (m, 2H), 1.66 – 1.61 (m, 2H), 1.50 – 1.44 (m, 2H). **^{13}C NMR** (151 MHz, Chloroform-*d*) δ 200.4, 137.0, 132.9, 128.6, 128.0, 62.7, 38.4, 32.5, 25.4, 23.9. **IR (neat):** ν 3364, 2934, 2864, 2325, 2111, 1918, 1679, 1596, 1448, 1221, 1050, 750, 691. **HRMS (ESI):** m/z $[M+Na]^+$ calcd. for $[C_{12}H_{16}O_2Na]^+$: 215.1043, found: 215.1042.

These data are in agreement with those reported previously in the literature.[9]

7-Hydroxy-1-phenylheptan-1-one (2h)

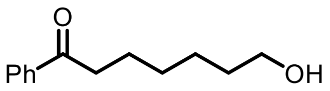

Prepared following general procedure 1, **2h** was obtained after purification by column chromatography (DCM: acetone 95:5) as a colorless oil (18.9 mg, 92 µmol, 46%). R_f = 0.19 (DCM: acetone 95:5) **^{1}H NMR** (600 MHz, Chloroform-*d*) δ 7.99 – 7.94 (m, 2H), 7.57 – 7.54 (m, 1H), 7.46 (t, *J* = 7.7 Hz, 2H), 3.65 (t, *J* = 6.6 Hz, 2H), 2.98 (t, *J* = 7.3 Hz, 2H), 1.81 – 1.23 (m, 8H). **^{13}C NMR** (151 MHz, Chloroform-*d*) δ 200.6, 137.2, 133.1, 128.7, 128.2, 63.1, 38.6, 32.8, 29.3, 25.8, 24.4. **IR (neat):** ν 3330, 2928, 2855, 1680, 1595, 1407, 1372, 1218, 1010, 967, 725, 684 cm^{-1}. **HRMS (ESI):** m/z $[M+Na]^+$ calculated for $[C_{13}H_{18}O_2Na]^+$: 229.1199, found: 229.1193.

These data are in agreement with those reported previously in the literature.[5]

8-Hydroxy-1-phenyloctan-1-one (2i)

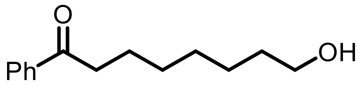

Prepared following general procedure 1, **2i** was obtained after purification by column chromatography (DCM: acetone 95:5) as a colorless oil (15.0 mg, 68 µmol, 34%). R_f = 0.33 (DCM: acetone 95:5). **^{1}H NMR** (600 MHz, Chloroform-*d*) δ 7.98 – 7.94 (m, 2H), 7.55 (t, *J* = 7.4 Hz, 1H), 7.46 (t, *J* = 7.7 Hz, 2H), 3.64 (t, *J* = 6.6 Hz, 2H), 2.97 (t, *J* = 7.4 Hz, 2H), 1.75 (p, *J* = 7.4 Hz, 2H), 1.61 – 1.54 (m, 2H), 1.43 – 1.36 (m, 6H). **^{13}C NMR** (151 MHz, Chloroform-*d*) δ 200.7, 137.2, 133.0, 128.7, 128.2, 63.2, 38.6, 32.9, 29.5, 29.4, 25.7, 24.4. **IR (neat):** ν 3333, 2927, 2855, 1681, 1448, 1371, 1218, 1061, 728, 687 cm^{-1} **HRMS (ESI):** m/z $[M+Na]^+$ calculated for $[C_{14}H_{20}O_2Na]^+$: 243.1356, found: 243.1358.

These data are in agreement with those reported previously in the literature.[10]

1,10-Tetradecanediol (2j)

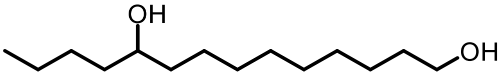

Prepared following general procedure 1, **2j** was obtained after purification by column chromatography (DCM: acetone =98:2) as a white solid (28.9 mg, 126 µmol, 63%). R_f = 0.46 (DCM: acetone =95:5) .**^{1}H NMR** (600 MHz, Chloroform-*d*) δ 3.62 (t, *J* = 6.6 Hz, 2H), 3.59 – 3.54 (m, 1H), 1.55 (p, *J* = 6.7 Hz, 2H), 1.50 – 1.36 (m, 7H), 1.35 – 1.25 (m, 15H), 0.90 (t, *J* = 6.9 Hz, 3H).**^{13}C NMR** (151 MHz, Chloroform-*d*) δ 71.0, 62.0, 36.5, 36.2, 31.8, 28.7, 28.5, 28.4, 26.8, 24.7, 24.6, 21.8, 13.1. **IR (neat):** ν 3352, 2921, 2852, 1464, 1352, 1132, 1060, 976 cm^{-1}. **HRMS (ESI):** m/z $[M+Na]^+$ calcd. for $[C_{14}H_{30}O_2Na]^+$: 253.2138, found: 253.2138.

These data are in agreement with those reported previously in the literature.[11]

2-(2-Hydroxyethyl)dibenzo[b,e]oxepin-11(6*H*)-one (2k)

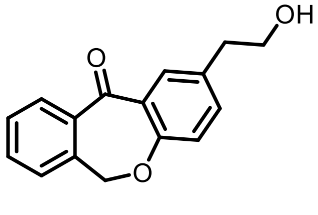

Prepared following general procedure 1, **2k** was obtained after purification by column chromatography (DCM: acetone 98:2), followed by preparative TLC (DCM: acetone 95:5) as a white solid (23.8 mg, 94 µmol, 47%). R_f = 0.65 (DCM: acetone 95:5.). **^{1}H NMR** (600 MHz, Chloroform-*d*) δ 8.08 (d, *J* = 2.4 Hz, 1H), 7.89 (dd, *J* = 7.7, 1.4 Hz, 1H), 7.55 (td, *J* = 7.5, 1.4 Hz, 1H), 7.46 (td, *J* = 7.6, 1.3 Hz, 1H), 7.38 – 7.33 (m, 2H), 7.00 (d, *J* = 8.3 Hz, 1H), 5.17 (s, 2H), 3.88 (t, *J* = 6.6 Hz, 2H), 2.88 (t, *J* = 6.6 Hz, 2H). **^{13}C NMR** (151 MHz, Chloroform-*d*) δ 191.1, 160.1, 140.5, 136.3, 135.7, 132.8, 132.4, 131.8, 129.5, 129.2, 127.8, 125.2, 120.9, 73.6, 63.5, 38.1. **IR (neat):** ν 3412, 2927, 2871, 1642, 1604, 1486, 1409, 1298, 1016, 759, 730 cm^{-1}. **HRMS (ESI):** m/z $[M+Na]^+$ calculated for $[C_{16}H_{14}O_3Na]^+$: 277.0835, found: 277.0835.

3-(1-Hydroxypropan-2-yl)phenyl)(phenyl)methanone (2l)

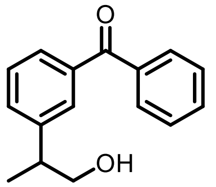

Prepared following general procedure 1 (with 10 mol% of the catalyst), **2l** was obtained after purification by column chromatography (DCM: acetone 97:3) as white solid (10.1 mg, 42 µmol, 21%). R_f = 0.66 (DCM: acetone 97:3). **^{1}H NMR** (600 MHz, Chloroform-*d*) δ 7.82 – 7.78 (m, 2H), 7.71 (t, *J* = 1.8 Hz, 1H), 7.63 (dt, *J* = 7.5, 1.5 Hz, 1H), 7.58 (d, *J* = 7.5 Hz, 1H), 7.48 (t, *J* = 7.6 Hz, 3H), 7.43 (d, *J* = 7.6 Hz, 1H), 3.74 (dd, *J* = 6.7, 2.6 Hz, 2H), 3.03 (q, *J* = 6.9 Hz, 1H), 1.31 (d, *J* = 7.0 Hz, 3H). **^{13}C NMR** (151 MHz, Chloroform-*d*) δ 197.0, 144.4, 138.0, 137.7, 132.6, 131.8, 130.2, 129.1, 128.7, 128.5, 128.4, 68.6, 42.5, 17.7. **IR (neat):** ν 3457, 2942, 1775, 1658, 1451, 1259, 1080, 1015, 792 cm^{-1}. **HRMS (ESI):** m/z [M+Na]$^+$ calculated for $[C_{16}H_{16}O_2Na]^+$: 263.1042, found: 263.1044.

These data are in agreement with those reported previously in the literature.[12]

2-(1-Hydroxypropan-2-yl)dibenzo[b,f]thiepin-10(11*H*)-one (2m)

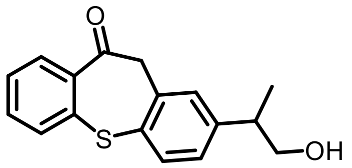

Prepared following general procedure 1 (with 10 mol% of the catalyst), **2m** was obtained after purification by column chromatography (DCM: acetone = 99:1) as a white solid (20.8 mg, 73 µmol, 37%). R_f = 0.5 (DCM: acetone = 99:1). **^{1}H NMR** (600 MHz, Chloroform-*d*) δ 8.19 (dd, *J* = 8.0, 1.6 Hz, 1H), 7.61 – 7.57 (m, 2H), 7.42 (td, *J* = 7.8, 1.6 Hz, 1H), 7.33 – 7.29 (m, 2H), 7.08 (dd, *J* = 7.9, 1.9 Hz, 1H), 4.39 – 4.29 (m, 2H), 3.73 – 3.65 (m, 2H), 2.95 (q, *J* = 6.9 Hz, 1H), 1.25 (d, *J* = 7.0 Hz, 3H). **^{13}C NMR** (151 MHz, Chloroform-*d*) δ 191.7, 146.5, 140.5, 138.0, 136.3, 132.6, 131.6, 131.5, 131.0, 128.6, 126.9, 126.6, 68.5, 51.2, 42.3, 17.7. **IR (neat):** ν 3420, 1667, 1587, 1424, 1428, 1036, 755, 730 cm^{-1}. **HRMS (ESI):** m/z [M+Na]$^+$ calculated for $[C_{17}H_{16}O_2SNa]^+$: 307.0763, found: 307.0756.

n-Pent-4-enyl alcohol (2n)

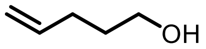

Prepared following general procedure 1, **2n** was obtained crude (^{1}H NMR yield 45% , with respect to $CHBr_3$ as the internal standard).**^{1}H NMR** (600 MHz, Chloroform-*d*) δ 5.86 – 5.72 (m, 1H), 5.00 (d, *J* = 16.6 Hz, 1H), 4.93 (d, *J* = 10.2 Hz, 1H), 3.90 – 3.75 (m, 2H), 2.11 – 2.07 (m, 2H), 1.64 (t, *J* = 7.3, 7.3 Hz, 2H).

These data are in agreement with those reported previously in the literature.[8]

10-Undecen-1-ol (2o)

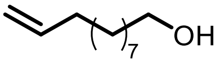

Prepared following general procedure 1, **2o** was obtained crude (^{1}H NMR yield 50%, with respect to $CHBr_3$ as the internal standard). **^{1}H NMR** (600 MHz, Chloroform-*d*) δ 5.82 – 5.73 (m, 1H), 5.00 – 4.94 (m, 1H), 4.91 (dd, *J* = 10.2, 1.9 Hz, 1H), 3.81 (d, *J* = 6.8 Hz, 2H).

These data are in agreement with those reported previously in the literature.[13]

3-(4-Ethynylphenyl)-1-phenylpropan-1-ol (2p)

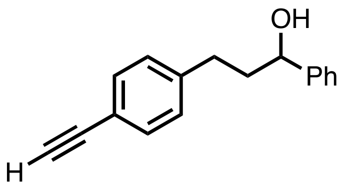

Prepared following general procedure 1 with EtOAc as solvent. **2p** was obtained after purification by column chromatography (pentane: Et_2O:Et_3N 20:79:1) as a colorless oil (26.9 mg, 114 µmol, 57%).**^{1}H NMR** (600 MHz, Chloroform-*d*) δ 7.41 (d, *J* = 7.9 Hz, 2H), 7.38 – 7.32 (m, 4H), 7.32 – 7.27 (m, 1H), 7.15 (d, *J* = 7.8 Hz, 2H), 4.67 (dd, *J* = 8.0, 5.3 Hz, 1H), 3.04 (s, 1H), 2.80 – 2.63 (m, 2H), 2.18 – 1.97 (m, 2H).**^{13}C NMR** ^{13}C NMR (151 MHz, Chloroform-*d*) δ 144.4, 142.8, 132.2, 128.5, 128.4, 127.7, 125.9, 119.5, 83.7, 76.6, 73.7, 40.1, 31.9. **IR (neat):** ν 3555, 3287, 3030, 2927, 2332, 2106, 1678, 1605 cm^{-1}. **HRMS (ESI):** m/z [M+H]$^+$ calculated for $[C_{17}H_{17}O]^+$: 237.1274, found: 237.1281.

6-Phenyltetrahydro-2*H*-pyran-2-one (3a)

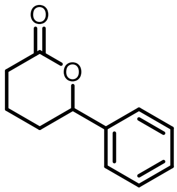

Prepared following general procedure 2, **3a** was obtained after purification by column chromatography (DCM: Et_3N 99:1) as a white solid (20.6 mg, 117 µmol, 58%). R_f = 0.41 (DCM: Et_3N 99:1).**^{1}H NMR** (600 MHz, Chloroform-*d*) δ 7.41 – 7.30 (m, 5H), 5.36 (dd, *J* = 10.6, 3.4 Hz, 1H), 2.71 (dt, *J* = 17.8, 6.3 Hz, 1H), 2.58 (dt, *J* = 17.8, 7.9 Hz, 1H), 2.23 – 2.15 (m, 1H), 2.06 – 1.95 (m, 2H), 1.93 – 1.79 (m, 1H). **^{13}C NMR** (151 MHz, Chloroform-*d*) δ 171.3, 139.8, 128.6, 128.3, 125.7, 81.6, 30.5, 29.5, 18.6. **IR (neat):** ν 2949, 1723, 1457, 1343, 1281, 1241, 1159, 1029, 965, 932, 760, 701 cm^{-1}. **HRMS (ESI)**: m/z [M+Na]$^+$ calcd. for $[C_{11}H_{12}O_2Na]^+$: 199.0729, found: 199.0728.

These data are in agreement with those reported previously in the literature.[14]

6-(*p*-tolyl)tetrahydro-2*H*-pyran-2-one (3b)

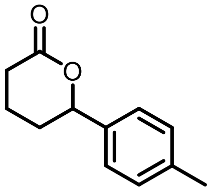

Prepared following general procedure 2, **3b** was obtained after purification by column chromatography (DCM: Et_3N 99:1) as a white solid (21.3 mg, 112 µmol, 56%). R_f = 0.45 (DCM: Et_3N 99:1). **^{1}H NMR** (600 MHz, Chloroform-*d*) δ 7.23 (d, *J* = 7.9 Hz, 2H), 7.18 (d, *J* = 8.0 Hz, 2H), 5.32 (dd, *J* = 10.6, 3.3 Hz, 1H), 2.69 (dt, *J* = 17.7, 6.5 Hz, 1H), 2.56 (dt, *J* = 17.6, 7.9 Hz, 1H), 2.35 (s, 3H), 2.18 – 2.08 (m, 1H), 2.01 – 1.94 (m, 2H), 1.91 – 1.80 (m, 1H). **^{13}C NMR** (151 MHz, Chloroform-*d*) δ 171.5, 138.1, 136.8, 129.3, 125.7, 81.6, 30.5, 29.5, 21.1, 18.6. **IR (neat):** ν 2956, 1720, 1457, 1350, 1240, 1165, 1038, 962, 930, 822, 763 cm^{-1}. **HRMS (ESI)**: m/z [M+Na]$^+$ calcd. for $[C_{12}H_{14}O_2Na]^+$: 213.0886, found: 213.0884.

These data are in agreement with those reported previously in the literature.[15]

6-(4-Fluorophenyl)tetrahydro-2*H*-pyran-2-one (3c)

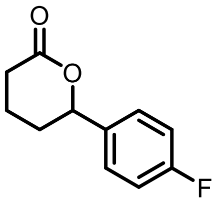

Prepared following general procedure 2, **3c** was obtained after purification by column chromatography (DCM: Et_3N 99:1) as a white solid (19.2 mg, 99 µmol, 49%). R_f =0.48 (DCM: Et_3N 99:1). **^{1}H NMR** (600 MHz, Chloroform-*d*) δ 7.36 – 7.28 (m, 2H), 7.06 (t, *J* = 8.6 Hz, 2H), 5.32 (dd, *J* = 10.8, 3.3 Hz, 1H), 2.71 (dt, *J* = 17.8, 6.2 Hz, 1H), 2.57 (dt, *J* = 17.8, 8.0 Hz, 1H), 2.15 (dq, *J* = 13.6, 4.4 Hz, 1H), 2.07 – 1.94 (m, 2H), 1.84 (ddt, *J* = 14.1, 10.8, 8.0 Hz, 1H).**^{13}C NMR** (151 MHz, Chloroform-*d*) δ 171.4, 162.6 (d, $^1J_{CF}$= 246.8 Hz), 135.5 (d, $^4J_{CF}$ = 3.1 Hz), 127.6 (d, $^3J_{CF}$ = 8.4 Hz), 115.5 (d, $^2J_{CF}$ = 21.4 Hz), 81.1, 30.5, 29.4, 18.6. **IR (neat):** ν 2962, 1721, 1604, 1511, 1367, 1237, 1160, 1043, 963, 932, 837, 775 cm^{-1}. **HRMS (ESI)**: m/z [M+Na]$^+$ calcd. for $[C_{11}H_{11}O_2FNa]^+$: 217.0635, found: 217.0636.

These data are in agreement with those reported previously in the literature.[15]

6-(4-Chlorophenyl)tetrahydro-2*H*-pyran-2-one (3d)

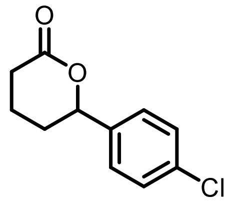

Prepared following general procedure 2, **3d** was obtained after purification by column chromatography (DCM: Et_3N 99:1) as a white solid (27.1 mg, 129 µmol, 64%). R_f = 0.58 (DCM: Et_3N 99:1). **^{1}H NMR** (600 MHz, Chloroform-*d*) δ 7.36 – 7.32 (m, 2H), 7.28 (m, 2H), 5.32 (dd, *J* = 10.8, 3.4 Hz, 1H), 2.70 (dt, *J* = 17.9, 6.3 Hz, 1H), 2.57 (dt, *J* = 17.8, 8.0 Hz, 1H), 2.18 – 2.10 (m, 1H), 2.02 – 1.95 (m, 2H), 1.86 – 1.75 (m, 1H). **^{13}C NMR** (151 MHz, Chloroform-*d*) δ 171.2, 138.2, 134.1, 128.8, 127.1, 80.9, 30.5, 29.4, 18.6. **IR (neat):** ν 2956, 1719, 1490, 1348, 1238, 1163, 1085, 1042, 962, 931, 840, 816 cm^{-1} **HRMS (ESI)**: m/z [M+Na]$^+$ calcd. for $[C_{11}H_{11}O_2ClNa]^+$: 233.0340, found: 233.0340.

These data are in agreement with those reported previously in the literature.[15]

6-(4-Bromophenyl)tetrahydro-2*H*-pyran-2-one (3e)

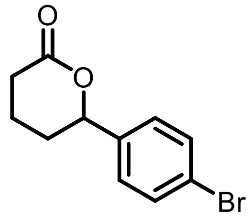

Prepared following general procedure 2, **3e** was obtained after purification by column chromatography (DCM: Et_3N 99:1) as a colorless oil (30.4 mg, 119 µmol, 60%). R_f = 0.58 (DCM: Et_3N 99:1). **^{1}H NMR** (600 MHz, Chloroform-*d*) δ 7.53 – 7.47 (m, 2H), 7.24 – 7.19 (m, 2H), 5.30 (dd, *J* = 10.7, 3.3 Hz, 1H), 2.73 – 2.66 (m, 1H), 2.56 (dt, *J* = 17.8, 8.0 Hz, 1H), 2.18 – 2.11 (m, 1H), 2.01 – 1.95 (m, 2H), 1.86 – 1.76 (m, 1H). **^{13}C NMR** (151 MHz, Chloroform-*d*) δ 171.1, 138.9, 131.9, 127.5, 122.3, 81.0, 30.6, 29.5, 18.7. **IR (neat):** ν 1721, 1487, 1344, 1237, 1041, 1010, 814 cm^{-1}. **HRMS (ESI):** m/z [M+Na]$^+$ calculated for $[C_{11}H_{11}O_2BrNa]^+$: 276.9835, found: 276.9834.

6-(4-Methoxyphenyl)tetrahydro-2*H*-pyran-2-one (3f)

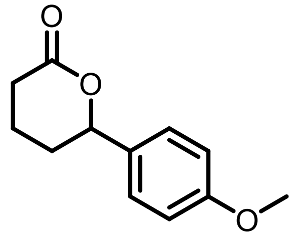

Prepared following general procedure 2, **3f** was obtained after purification by column chromatography (DCM: Et_3N 99:1) as a brown solid (9.5 mg, 46 µmol, 23%). R_f =0.21 (DCM: Et_3N 99:1). **^{1}H NMR** (600 MHz, Chloroform-*d*) δ 7.29 – 7.26 (m, 2H), 6.93 – 6.89 (m, 2H), 5.30 (dd, *J* = 10.7, 3.3 Hz, 1H), 3.81 (s, 3H), 2.73 – 2.66 (m, 1H), 2.60 – 2.53 (m, 1H), 2.17 – 2.10 (m, 1H), 2.04 – 1.95 (m, 2H), 1.91 – 1.84 (m, 1H). **^{13}C NMR** (151 MHz, Chloroform-*d*) δ 171.6, 159.7, 132.0, 127.3, 114.1, 81.6, 55.5, 30.5, 29.6, 18.8. **IR (neat):** ν 2925, 1714, 1611, 1513, 1240, 1176, 1032, 822 cm^{-1} **HRMS (ESI)**: m/z [M+Na]$^+$ calcd. for $[C_{12}H_{14}O_3Na]^+$: 229.0835, found: 229.0832.

These data are in agreement with those reported previously in the literature.[16]

6-Hydroxy-6-phenylhexanoic acid (3g)

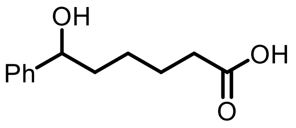

Prepared following general procedure 2, **3g** was obtained after purification by column chromatography (EtOAc: pentane 30:70+1%AcOH) as a colorless oil (28.9 mg, 139 µmol, 69%). R_f = 0.44 (EtOAc: pentane 40:60+1% AcOH). **^{1}H NMR** (600 MHz, Chloroform-*d*) δ 7.42 – 7.15 (m, 5H), 4.66 (dd, *J* = 7.6, 5.7 Hz, 1H), 2.33 (t, *J* = 7.4 Hz, 2H), 1.86 – 1.74 (m, 1H), 1.75 – 1.59 (m, 3H), 1.51 – 1.25 (m, 2H). **^{13}C NMR** (151 MHz, Chloroform-*d*) δ 179.6, 144.5, 128.5, 127.6, 125.9, 74.4, 38.5, 25.3, 24.6. **IR (neat):** ν = 3400, 2980, 2938, 1710, 1451, 1374, 1143, 980, 848, 699, 672 cm^{-1}. **HRMS (ESI):** m/z [M+Na]$^+$ calcd. for $[C_{12}H_{16}O_3Na]^+$: 231.0988, found: 231.0992.

7-Hydroxy-7-phenylheptanoic acid (3h)

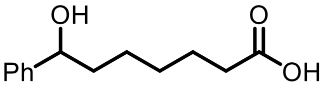

Prepared following general procedure 2 (on a 0.1 mmol scale), **3h** was obtained after purification by column chromatography (EtOAc: pentane 40:60+1% AcOH) as a colorless oil (14.4 mg, 65 µmol, 65%). R_f = 0.39 (EtOAc: pentane 40:60+1% AcOH). **^{1}H NMR** (600 MHz, Chloroform-*d*) δ 7.31 – 7.23 (m, 4H), 7.22 – 7.18 (m, 1H), 4.59 (t, *J* = 6.6 Hz, 1H), 2.25 (t, *J* = 7.4 Hz, 2H), 1.77 – 1.69 (m, 1H), 1.68 – 1.60 (m, 1H), 1.55 (p, *J* = 7.5 Hz, 2H), 1.41 – 1.17 (m, 4H). **^{13}C NMR** (151 MHz, Chloroform-*d*) δ 179.6, 144.8, 128.6, 127.7, 126.0, 74.7, 38.9, 34.0, 29.0, 25.5, 24.7. **IR (neat):** ν = 2933, 2860,1705, 1453, 1409, 1201, 1026, 760, 699 cm^{-1}. **HRMS (ESI):** m/z [M+Na]$^+$ calcd. for $[C_{13}H_{18}O_3Na]^+$: 245.1148, found: 245.1144.

8-Hydroxy-8-phenyloctanoic acid (3i)

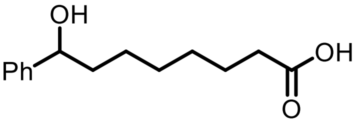

Prepared following general procedure 2 (on a 0.1 mmol scale), **3i** was obtained after purification by column chromatography (acetone: pentane 10:90 + 1% AcOH) as a white solid (15.0 mg, 63 µmol, 63%). R_f = 0.56 (acetone: pentane 30:70 + 1%AcOH). **^{1}H NMR** (600 MHz, Chloroform-*d*) δ 7.37 – 7.31 (m, 4H), 7.29 – 7.25 (m, 1H), 4.66 (dd, *J* = 7.5, 5.8 Hz, 1H), 2.32 (t, *J* = 7.5 Hz, 2H), 1.84 – 1.76 (m, 1H), 1.74 – 1.67 (m, 1H), 1.61 (p, *J* = 7.4 Hz, 2H), 1.46 – 1.38 (m, 1H), 1.35 – 1.25 (m, 5H). **^{13}C NMR** (151 MHz, Chloroform-*d*) δ 179.6, 144.9, 128.6, 127.7, 126.0, 74.8, 39.1, 34.1, 29.2, 29.1, 25.7, 24.7. **IR (neat):** ν = 3354, 3030, 2931, 2858, 1706, 1452, 1408, 1205, 1090, 1030, 915, 759, 699 cm^{-1}. **HRMS (ESI):** m/z [M+Na]$^+$ calcd. for $[C_{14}H_{20}O_3Na]^+$: 259.1305, found: 259.1302.

10-Hydroxytetradecanoic acid (3j)

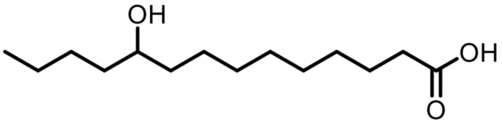

Prepared following general procedure 2, **3j** was obtained after purification by column chromatography (DCM: AcOH =99:1) as a white solid (23.1 mg, 95 µmol, 48%). R_f = 0.4 (DCM: AcOH =99:1) **^{1}H NMR** (600 MHz, Chloroform-*d*) δ 3.59 (dt, *J* = 7.5, 3.6, Hz, 1H), 2.38 (td, *J* = 7.5, 2.7 Hz, 1H), 2.33 (t, *J* = 7.5 Hz, 2H), 1.62 (q, *J* = 7.5 Hz, 2H), 1.54 (dd, *J* = 9.5, 5.5 Hz, 1H), 1.47 – 1.21 (m, 21H, overintegrates by 4 protons due to an overlap with water peak from chloroform-*d*), 0.90 (td, *J* = 7.4, 2.6 Hz, 3H). **^{13}C NMR** (151 MHz, Chloroform-*d*) δ 178.3, 71.1, 41.8, 41.5, 36.4, 36.1, 33.0, 28.6, 28.3, 28.1, 28.0, 26.8, 24.5, 23.6, 21.7, 13.1. **IR (neat):** ν 3397, 2923, 2852, 1709, 1463, 1434, 1259, 1022, 798 cm^{-1}. **HRMS (ESI):** m/z [M+Na]$^+$ calcd. for $[C_{14}H_{28}O_3Na]^+$: 267.1931, found: 267.1926.

2-(11-Hydroxy-6,11-dihydrodibenzo[b,e]oxepin-2-yl)acetic acid (3k)

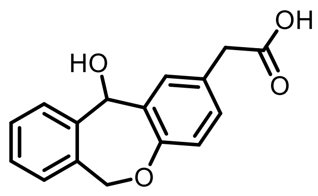

Prepared following general procedure 2 (with 10 mol% of the catalyst), **3k** was obtained after purification by column chromatography (pentane: acetone 70:30 +1% AcOH), followed by preparative TLC (pentane: acetone 60:40 +1% AcOH) as a white solid (21.7 mg, 80 µmol, 40%). R_f =0.37 (pentane: acetone 60:40 +1% AcOH). ^{1}H NMR (400 MHz, Chloroform-*d*) δ 7.45 (d, *J* = 7.1 Hz, 1H), 7.36 – 7.30 (m, 3H), 7.19 (dd, *J* = 8.4, 2.4 Hz, 1H), 6.87 (d, *J* = 8.4 Hz, 1H), 6.76 (s, 1H), 5.92 (d, *J* = 12.6 Hz, 1H), 4.93 (d, *J* = 12.6 Hz, 1H), 3.59 (s, 2H).**^{13}C NMR** (151 MHz, Chloroform-*d*) δ 177.3, 169.9, 157.0, 137.5, 135.4, 134.4, 132.0, 129.7, 129.3, 129.0, 126.1, 122.5, 120.9, 77.7, 70.6, 40.0.. **IR (neat):** ν 2924 (br), 1710, 1497, 1227, 1127, 1008, 753 cm^{-1}. **HRMS (ESI)**: m/z [M-CO$_2$]$^+$ calcd. for [$C_{15}H_{14}O_2$]$^+$: 226.0994, found:226.0962.

The isolated sample is contaminated with acetic acid (its mass has been subtracted before determining the yield) as after further concentration the product decomposed.

2-(3-(Hydroxy(phenyl)methyl)phenyl)propanoic acid (3l)

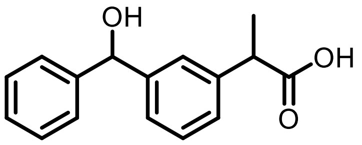

Prepared following general procedure 2 (with 10 mol% of the catalyst), **3l** was obtained after purification by column chromatography (DCM: acetone 95:5 + 1% AcOH) as a white solid (19.7 mg, 77 µmol, 39%). R_f = 0.6 (DCM: acetone 95:5+ 1% AcOH). **^{1}H NMR** (600 MHz, Chloroform-*d*) δ 7.39 – 7.22 (m, 9H), 5.83 (s, 1H), 3.73 (dd, *J* = 7.2, 2.0 Hz, 1H), 1.50 (dd, *J* = 7.2, 2.5 Hz, 3H). **^{13}C NMR** (151 MHz, Chloroform-*d*) δ 179.8, 144.3, 143.7, 140.2, 129.0, 128.7, 127.8, 126.9, 126.7, 126.1, 125.8, 76.3, 45.4, 18.3. **IR (neat):** ν 3030, 2932, 1705, 1602, 1453, 1194, 1022, 907. **HRMS (ESI):** m/z [M+Na]$^+$ calculated for [$C_{16}H_{16}O_3Na$]$^+$: 279.0992, found: 279.0988.

These data are in agreement with those reported previously in the literature.[17]

2-(10-Hydroxy-10,11-dihydrodibenzo[b,f]thiepin-2-yl)propanoic acid (3m)

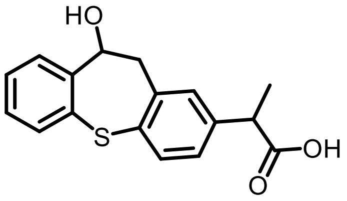

Prepared following general procedure 2 (with 10 mol% of the catalyst), **3m** was obtained after purification by column chromatography (DCM: acetone=99:1+ 1% AcOH) followed by preparative TLC (DCM: acetone 99:1+1% AcOH) as a white solid (8.4 mg, 28 µmol, 14%). R_f = 0.24 (DCM: acetone 99:1+ 1% AcOH). **^{1}H NMR** (600 MHz, Chloroform-*d*) δ 7.55 – 7.50 (m, 1H), 7.45 (ddd, *J* = 18.6, 7.9, 1.4 Hz, 2H), 7.28 – 7.24 (m, 1H), 7.22 (d, *J* = 2.0 Hz, 1H), 7.14 (td, *J* = 7.6, 1.5 Hz, 1H), 7.09 (dd, *J* = 8.0, 2.0 Hz, 1H), 5.43 – 5.32 (m, 1H), 3.79 – 3.64 (m, 2H), 3.37 (dd, *J* = 14.5, 8.7 Hz, 1H), 1.47 (d, *J* = 7.2 Hz, 3H). **^{13}C NMR** (151 MHz, Chloroform-*d*) δ 179.2, 142.6, 139.9, 139.4, 134.5, 133.0, 132.0, 131.4, 130.8, 130.7, 130.6, 69.8, 44.8, 41.3, 18.1. **IR (neat):** ν 3058, 2925, 1706, 1470, 1431, 1192, 1059, 1029, 907, 755, 730 cm^{-1}. **HRMS (ESI):** m/z [M+Na]$^+$ calculated for [$C_{17}H_{16}O_3SNa$]$^+$: 323.0707, found: 323.0712.

Valeric acid (3n) and **5-(4,4,5,5-tetramethyl-1,3,2-dioxaborolan-2-yl)pentanoic acid (3n')**

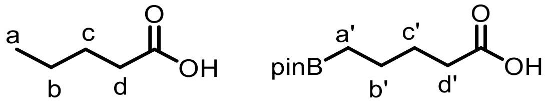

Prepared following general procedure 2, **3n** and **3n'** were obtained crude (^{1}H NMR yields with respect to $CHBr_3$ as the internal standard: 3n: 55% and 3n' 16%). **^{1}H NMR** (600 MHz, Chloroform-*d*) δ 2.30 (s, 4H, Hd + Hd'), 1.57 (d, *J* = 13.8 Hz, 4H, Hc + Hc'), 0.88 (d, *J* = 8.0 Hz, 3H, Ha), 0.75 (d, *J* = 7.8 Hz, 2H, Ha').

These data are in agreement with those reported previously in the literature.[18,19]

Undecanoic acid (3o) and **5-(4,4,5,5-tetramethyl-1,3,2-dioxaborolan-2-yl)undecanoic acid (3o')**

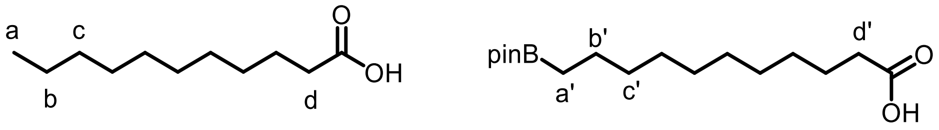

Prepared following general procedure 2, **3o** and **3o'** were obtained crude (^{1}H NMR yields with respect to $CHBr_3$ as the internal standard: 3o: 33 % and 3o' 43%).^{1}H NMR (600 MHz, Chloroform-*d*) δ 2.37 – 2.26 (m, 4H, Hd+ Hd'), 0.86 (d, *J* = 7.0 Hz, 3H, Ha), 0.75 (d, *J* = 7.9 Hz, 2H, Ha').

These data are in agreement with those reported previously in the literature.[20]

(*E*)-1-Phenyl-3-(4-(2-(4,4,5,5-tetramethyl-1,3,2-dioxaborolan-2-yl)vinyl)phenyl)propan-1-one (3p)

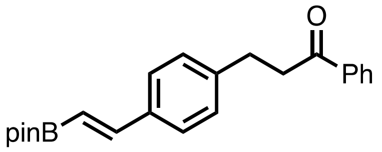

In an oven-dried 4 ml vial were introduced the cobalt catalyst (1 mol%, 1 µmol, 0.86 mg), the starting material (1.0 equiv, 0.1 mmol, 23.4 mg) when solid and a stirring bar. A cap with rubber septum was used to close the vial and the system was then purged with argon EtOAc (C = 1.0 M, 0.1 mL), HBpin (1.1 equiv, 0.11 mmol, 16 µL) were then added successively and the vial was exposed to blue LEDs overnight. After this time, the solution was diluted with Et_2O and washed two times with distilled water, the combined aqueous layers were extracted two times with Et_2O and the combined organic layers were washed with brine, dried ($MgSO_4$), filtered and concentrated under vacuum. The desired product was purified with column chromatography (Pentane: EtOAc= 90:10) and obtained as a white solid in (20.3 mg, 56 µmol, 56%).

^{1}H NMR (600 MHz, Chloroform-*d*) δ 7.95 (d, *J* = 7.4 Hz, 2H), 7.55 (t, *J* = 7.4 Hz, 1H), 7.48 – 7.40 (m, 4H), 7.37 (d, *J* = 18.4 Hz, 1H), 7.22 (d, *J* = 7.9 Hz, 2H), 6.12 (d, *J* = 18.4 Hz, 1H), 3.30 (t, *J* = 7.7 Hz, 2H), 3.07 (t, *J* = 7.7 Hz, 2H), 1.31 (s, 12H). **^{13}C NMR** (151 MHz, Chloroform-*d*) δ 199.1, 149.2, 142.2, 136.8, 135.6, 133.1, 128.7, 128.6, 128.0, 127.3, 83.3, 40.2, 29.9, 24.8. **IR (neat):** ν 2979, 2918, 1684, 1623, 1512 cm^{-1}. **HRMS (ESI):** m/z $[M+Na]^+$ calculated for $[C_{23}H_{27}O_3BNa]^+$: 385.1946, found: 385.1937.

6. Additional Substrates

6.1 5-Hexen-2-one

5-Hexen-2-one underwent the reaction under the optimised 'light conditions', which led to a mixture of hydroborated (42%) and hydrogenated (24%) products.

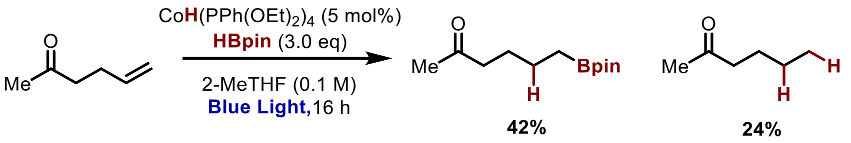

In the absence of light, the reaction has poor selectivity, resulting in ketone reduction, hydrogenation, hydroboration, and double bond isomerisation.

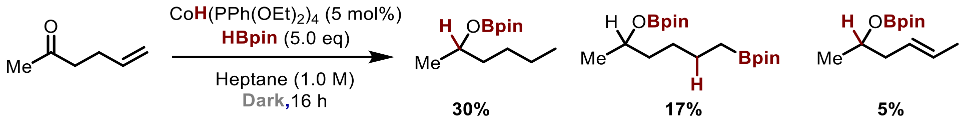

6.2 3-Benzoylpropionic acid

3-benzoylpropionic acid has been tested and in both sets of conditions ketone hydroboration is the favoured process.

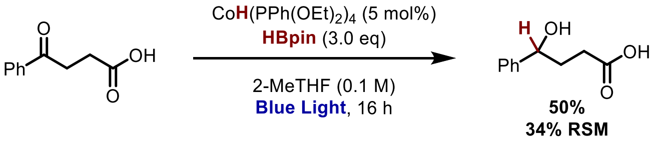

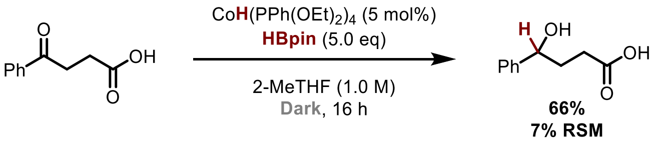

7. Comparison of selectivity

7.1 4-Pentenoic acid

Reaction of 4-pentenoic acid with pinacolborane in the absence of $CoH[PPh(OEt)_2]_4$ and without solvent led to the product arising from both acid reduction and hydroboration in 60% yield determined by comparison with an internal standard ($CHBr_3$).

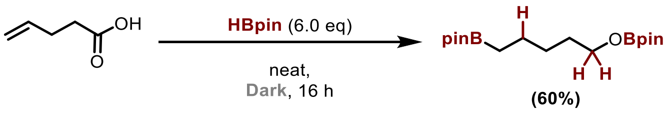

8. Mechanistic Studies

8.1 NMR experiments

8.1.1 ^{1}H NMR monitoring in dark conditions

For experiments under the dark conditions, we have carried out a preliminary analysis using ^{1}H NMR monitoring of the reaction. During the initial period of the reaction, we observe the acidic proton (H_a) disappearing and the protons at the α-position to the carboxylic acid becoming deshielded (H_b) – which we believe is consistent with what we outlined in Scheme 1c of the manuscript (reaction of the more nucleophilic carboxylic acid species with the borane). The peak corresponding to the hydride of CoH[PPh(OEt)$_2$]$_4$ (H_e) also decreases during this time. As product formation starts (H_d), there is an increase also in the quantity of H_e (at around 350-400 minutes) which matches with our suggestion that CoH[PPh(OEt)$_2$]$_4$ is generated during the catalytic cycle of product formation.

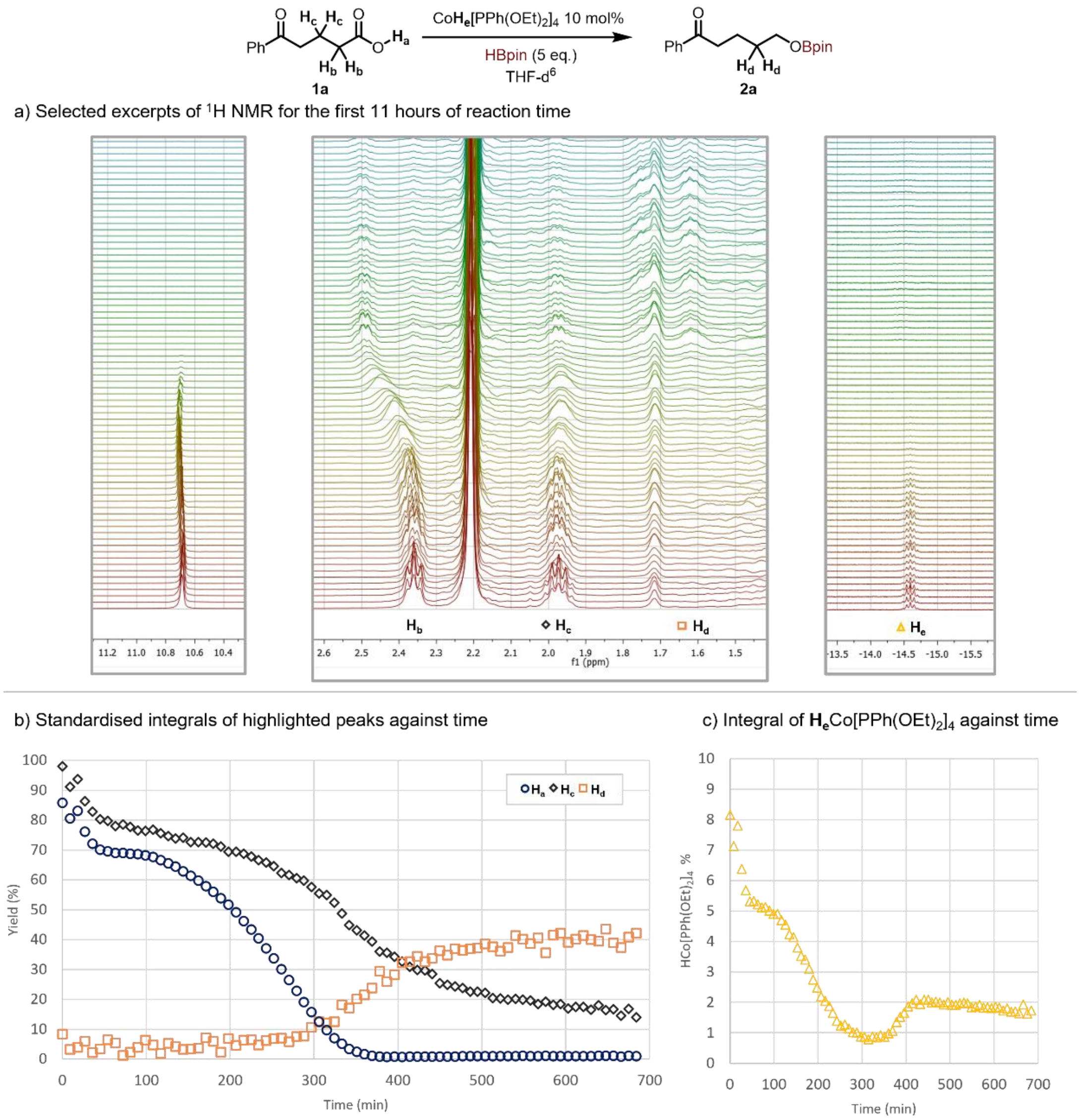

8.1.2 NMR monitoring in light conditions

When our benchmark substrate (spectrum **1**) was treated with a sub-stoichiometric amount of cobalt hydride and no HBpin, by ^{1}H NMR we can clearly observe disappearance of the peaks corresponding to the starting material and appearance of peaks that correspond to the free $PPh(OEt)_2$ ligand. The spectrum **2** represent the reaction mixture before irradiation; the spectra **3** and **4** after 30 minutes and 16 hours of irradiation, respectively. This suggests possible formation of (paramagnetic) cobalt carboxylate species which is likely an unproductive pathway that leads to catalyst decomposition. This implies that the reaction does not necessarily proceed as initially proposed in Scheme 1 of the paper with the 'free' acid.

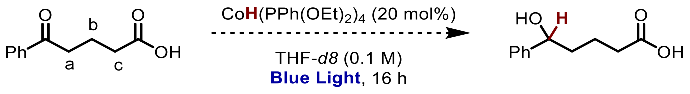

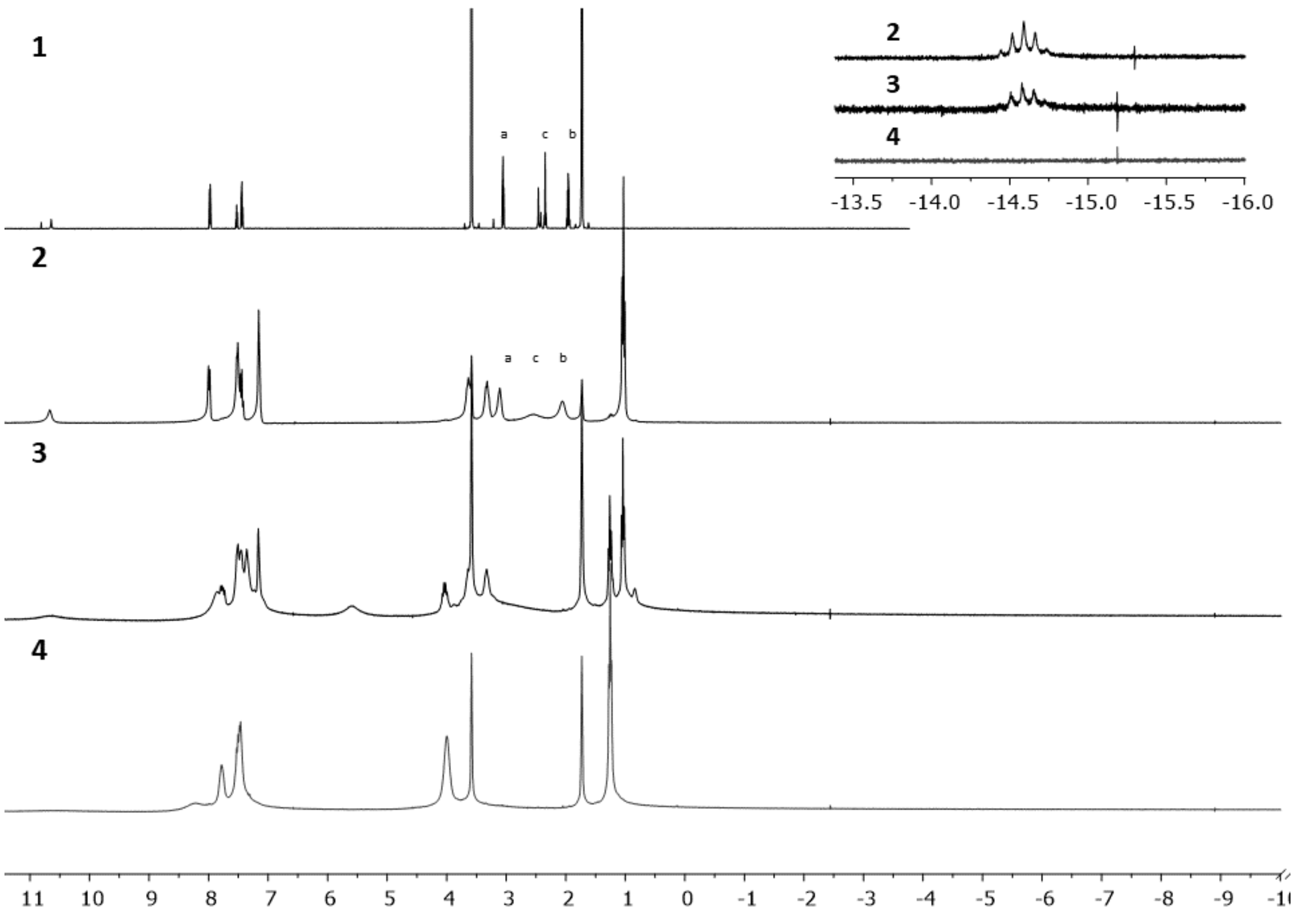

On the other hand, when we carried out the reaction under the optimised conditions, we observe a peak (-12.36 ppm) that is consistent with the reports of $\{CoH_2[PPh(OEt)_2]_4\}^+$ in the ^{1}H NMR.[21]

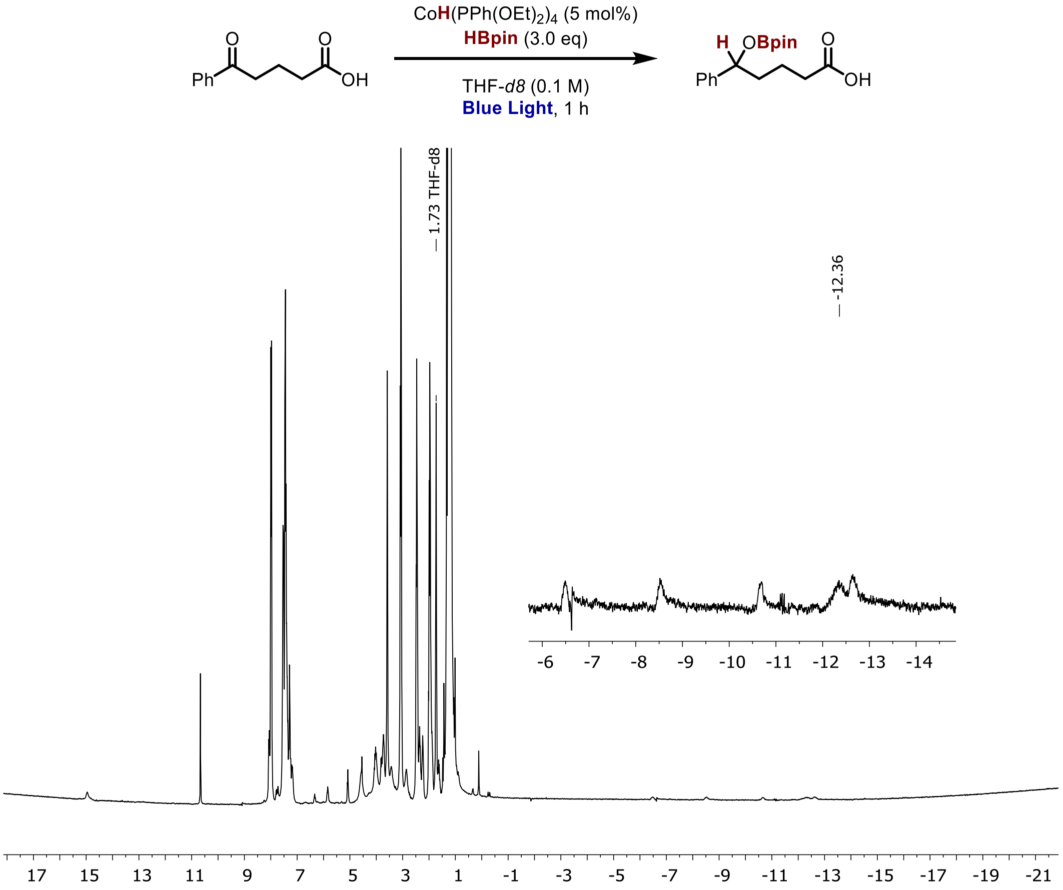

We also carried out the standard reaction under light irradiation, measuring the ^{1}H NMR at partial conversion. Here, along with formation of the product, we also observe that the peak for the protons at the α-position of the carboxylic moiety in the remaining starting material is significantly shifted (peak C, 2.3 to 2.5 ppm), as highlighted in the comparison below. This provides further strong evidence that the substrate initially reacts with one equivalent of HBpin, forming a mixed anhydride.

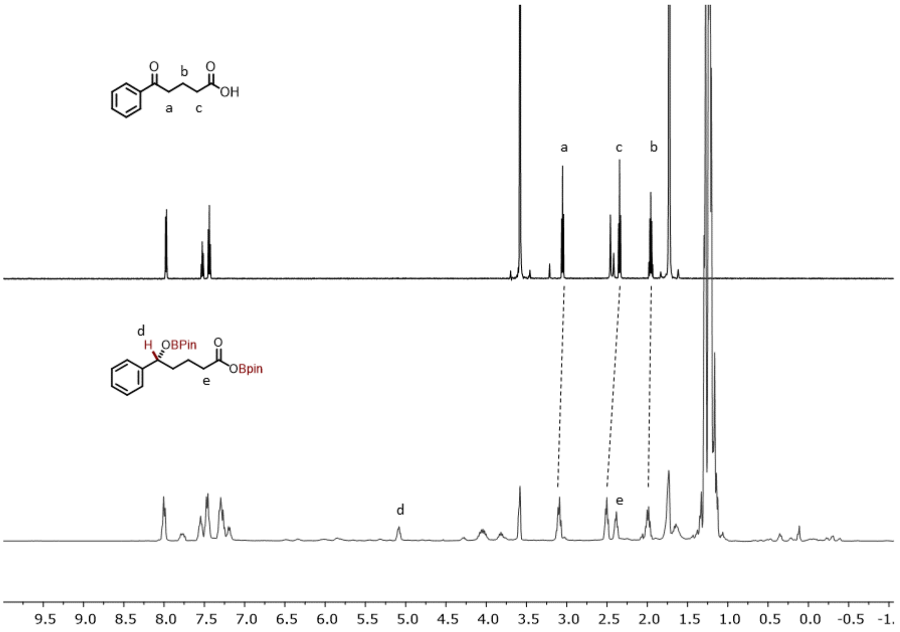

8.2 Deuterium incorporation experiments

- The cobalt deuteride CoD[PPh(OEt)$_2$]$_4$ was synthesised following the procedure described in Section 2, using EtOD and NaBD$_4$ and tested in the dark conditions. No deuterium incorporation in the product was observed.

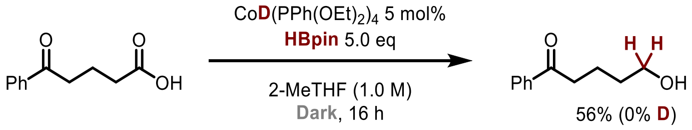

- Carrying out the benchmark reaction in the light with 20 mol% of CoD[PPh(OEt)$_2$]$_4$ led to good conversion but no detectable deuterium incorporation.

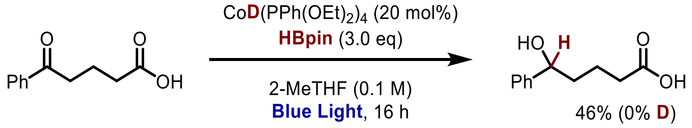

- Stochiometric and sub-stoichiometric reactions of different substrates with both CoD[PPh(OEt)$_2$]$_4$ & CoH[PPh(OEt)$_2$]$_4$ led to a rapid change of colour to green and then purple solutions.

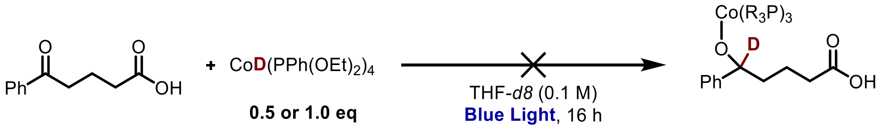

8.3 Experiments with TEMPO

The addition of TEMPO in catalytic (5 mol%) as well as stochiometric amounts (2 eq.) to the reaction mixture led to a decrease in acid reduction from 68% in optimized conditions to 25% and 7%, respectively. Unfortunately, despite several attempts, we were not able to identify the product from TEMPO trapping of the boraketyl radical.

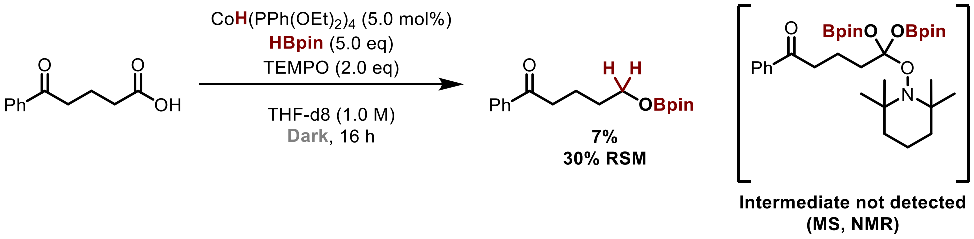

When mixing TEMPO with CoH[PPh(OEt)$_2$]$_4$, no change was observed.

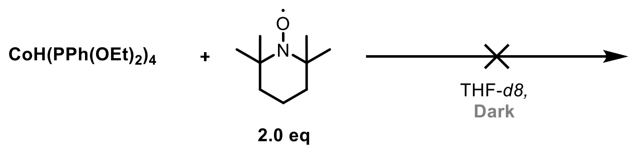

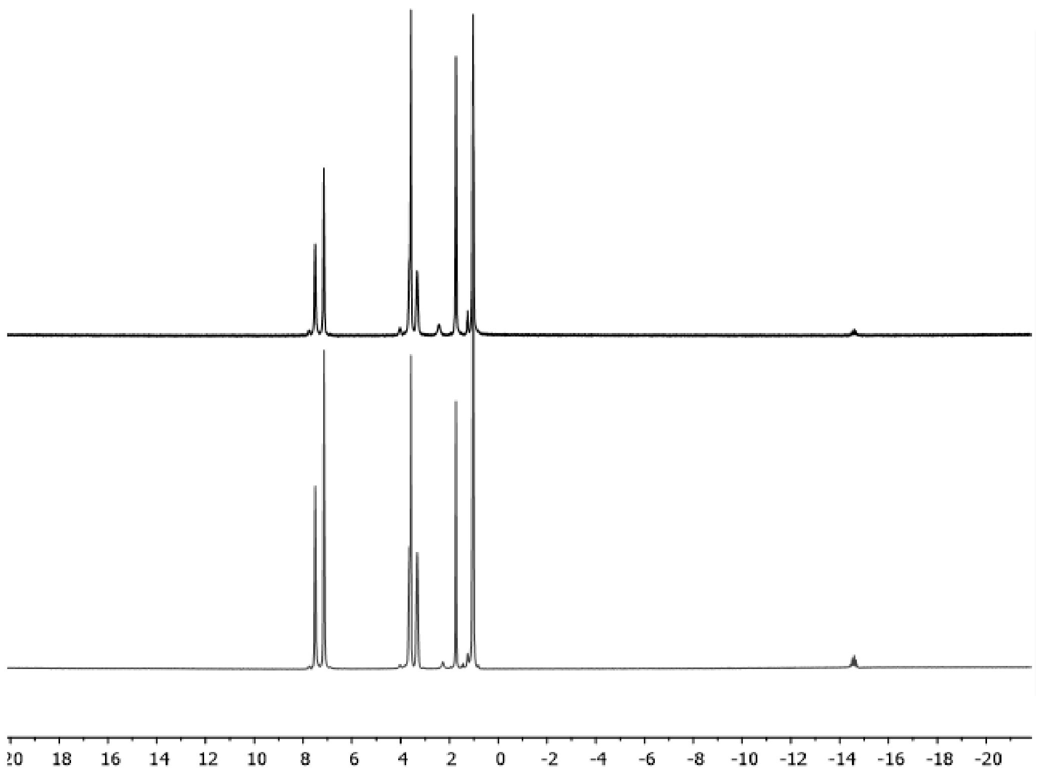

Top: CoH[PPh(OEt)2]4 in THF-d8; Bottom: CoH[PPh(OEt)2]4 +TEMPO in THF-d8 after 16 hours

8.4 The role of HBpin

To further support the dual role of HBpin acting both to protect the carboxylic acid functionality as well as the hydride source, we replaced it with isopropanol as an alternative hydride source which should not react with the carboxylic acid. In this case, no reduction occurs and only starting material is recovered which is consistent with HBpin playing a unique role for ketoacid substrates.

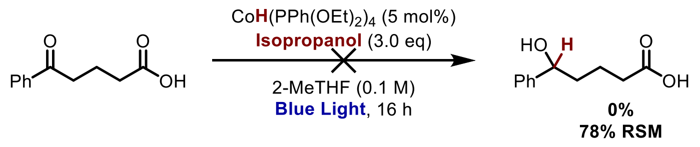

8.5 Alternative proposed mechanism

We therefore suggest that an alternative mechanism could be operative whereby the cobalt has a dual role. Here, the cobalt catalyst would both protect the acid as the anhydride *via* a mechanism that proceeds *via* $\{CoH_2[PPh(OEt)_2]_4\}^+$ as well as undergoing ligand photodissociation and subsequent insertion into the ketone.

This suggestion is based on:

1) The work of Onishi describing the reactivity of $CoH[PPh(OEt)_2]_4$ with formic acid;[21]
2) Our observation of the corresponding $\{CoH_2[PPh(OEt)_2]_4\}^+$ species (section 8.1.2);
3) The experiments which suggest the 'free' carboxylic acid functionality is not compatible with the ketone hydroboration catalytic cycle (sections 8.1.2 and 8.4);
4) NMR data consistent with formation of a mixed boron anhydride (section 8.1.2);

However, this is a preliminary suggestion and, at this stage, we cannot rule out other possibilities.

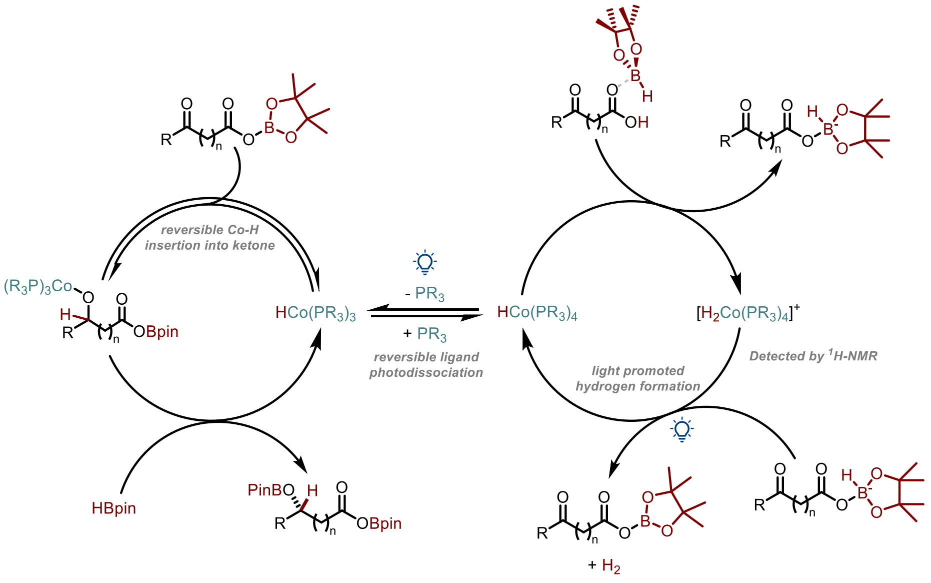

9. Copies of NMR spectra

***N*-Methoxy-*N*-methylundec-10-enamide (1j-I)**

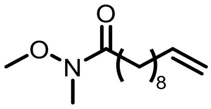

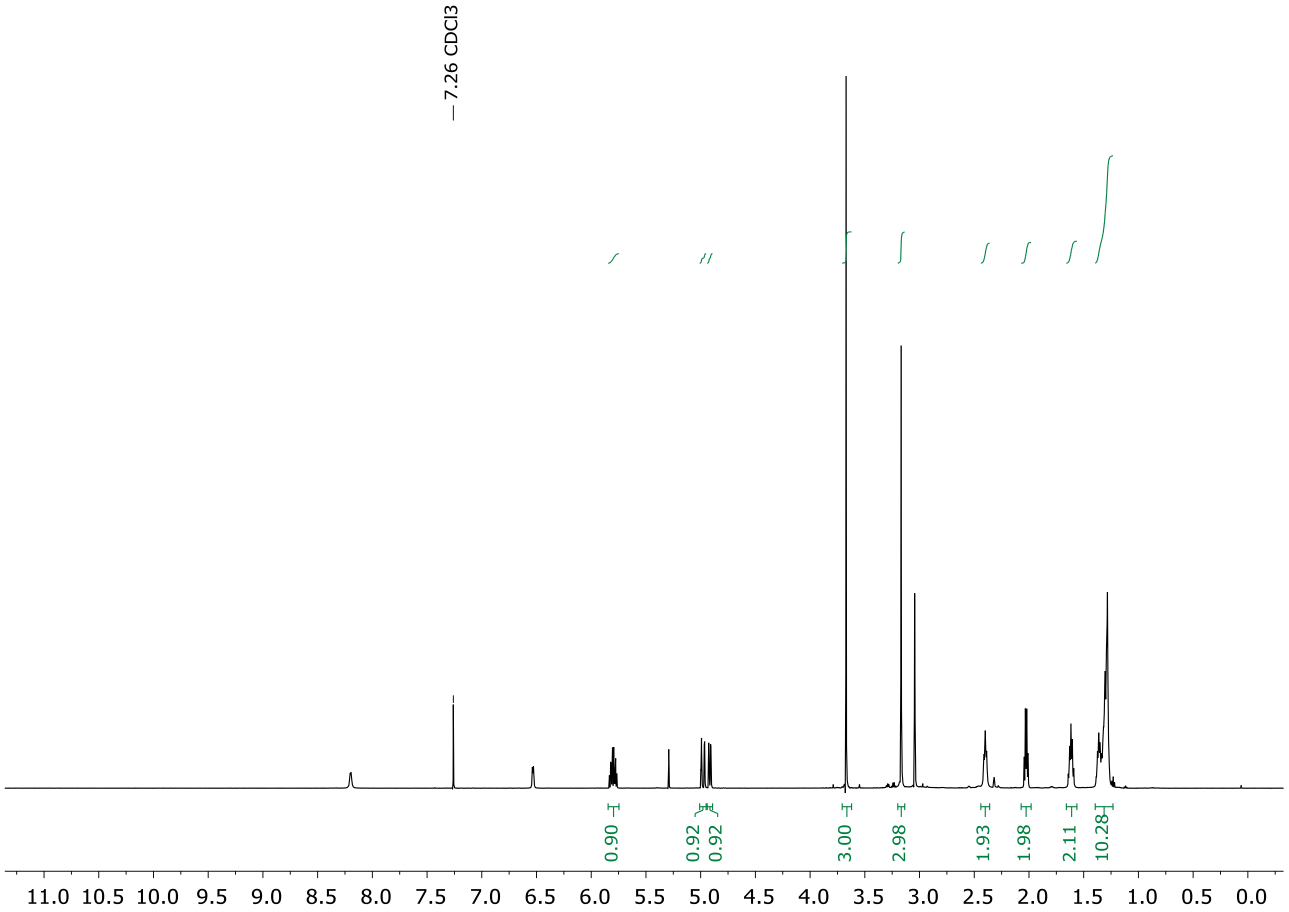

10-Oxotetradecanoic acid (1j)

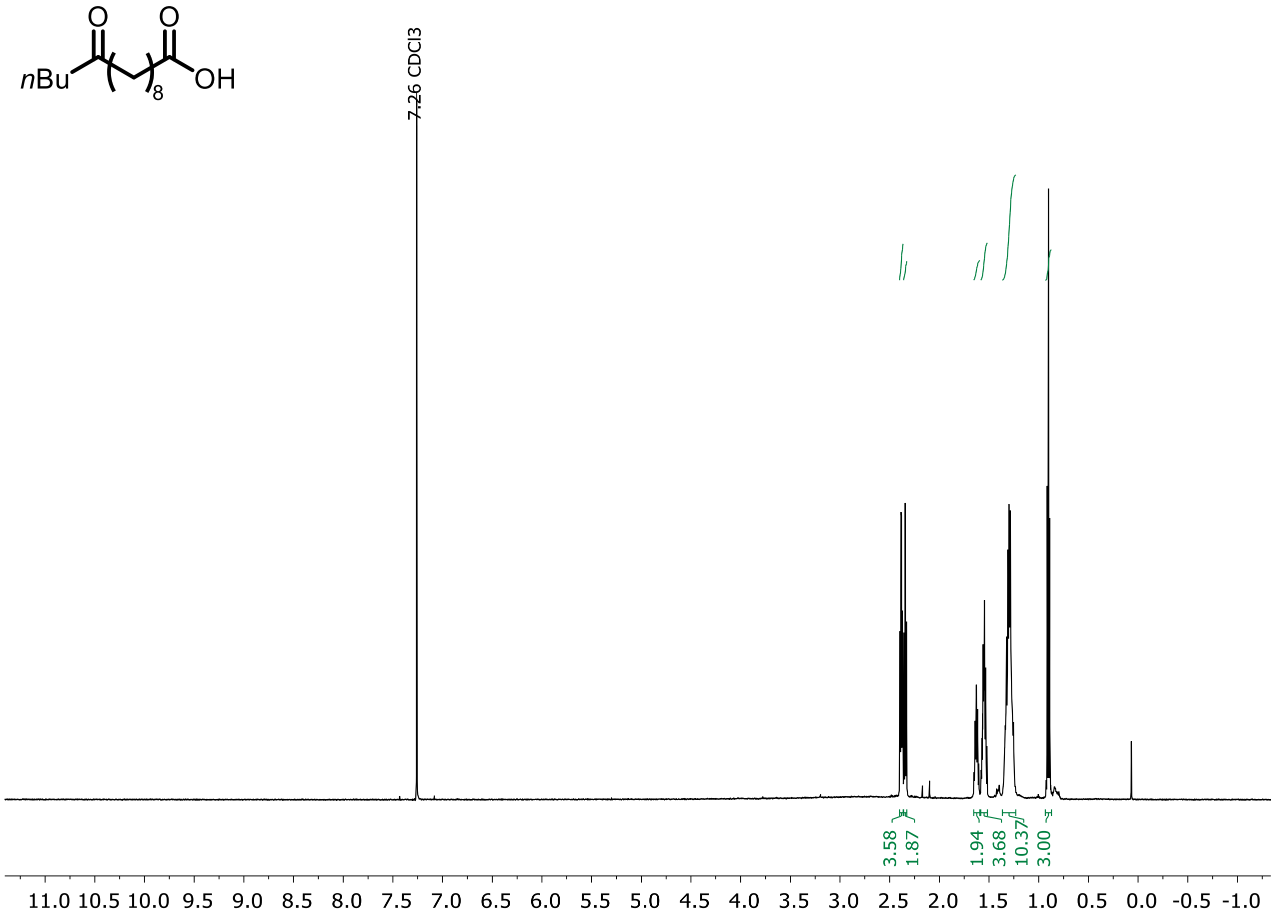

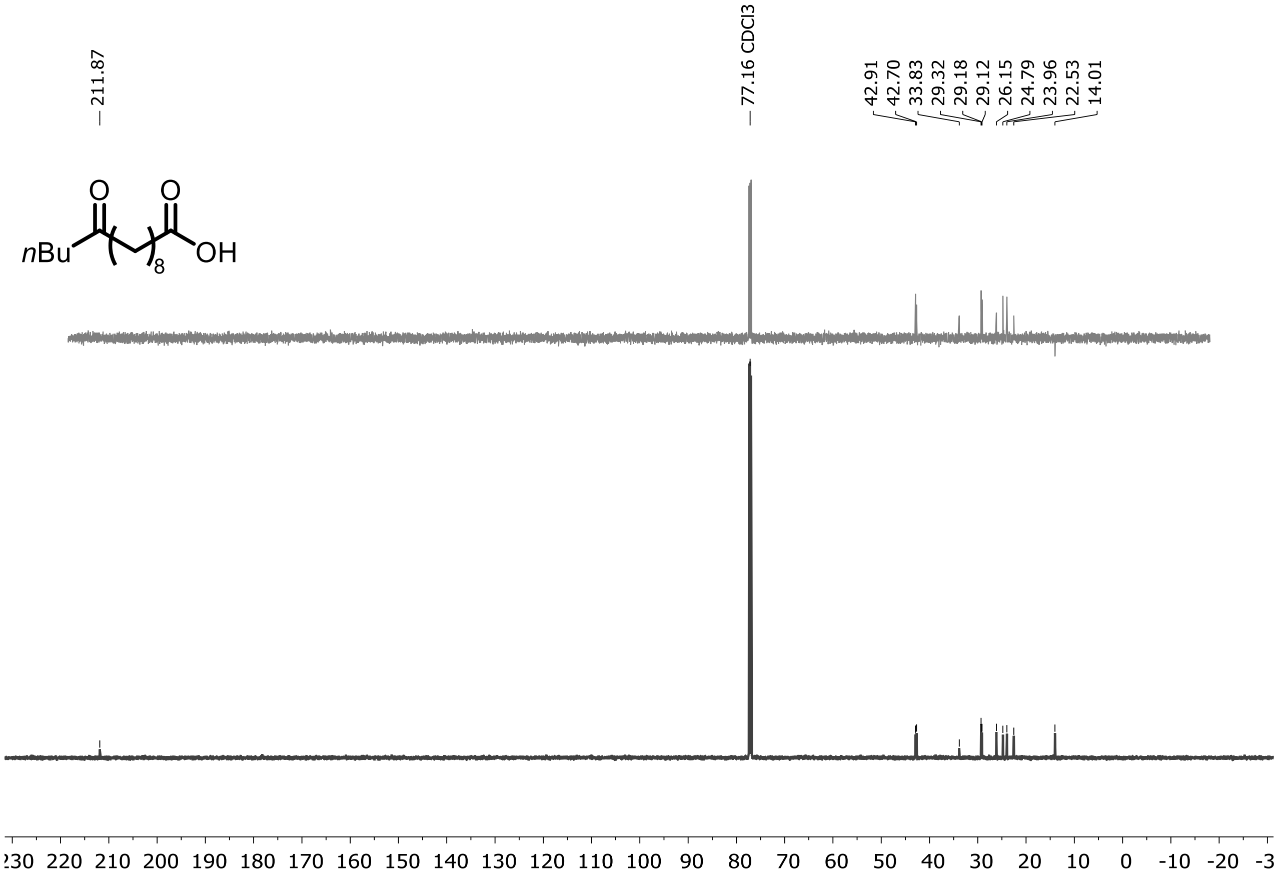

3-(4-Bromophenyl)-1-phenylpropan-1-one (1p-II)

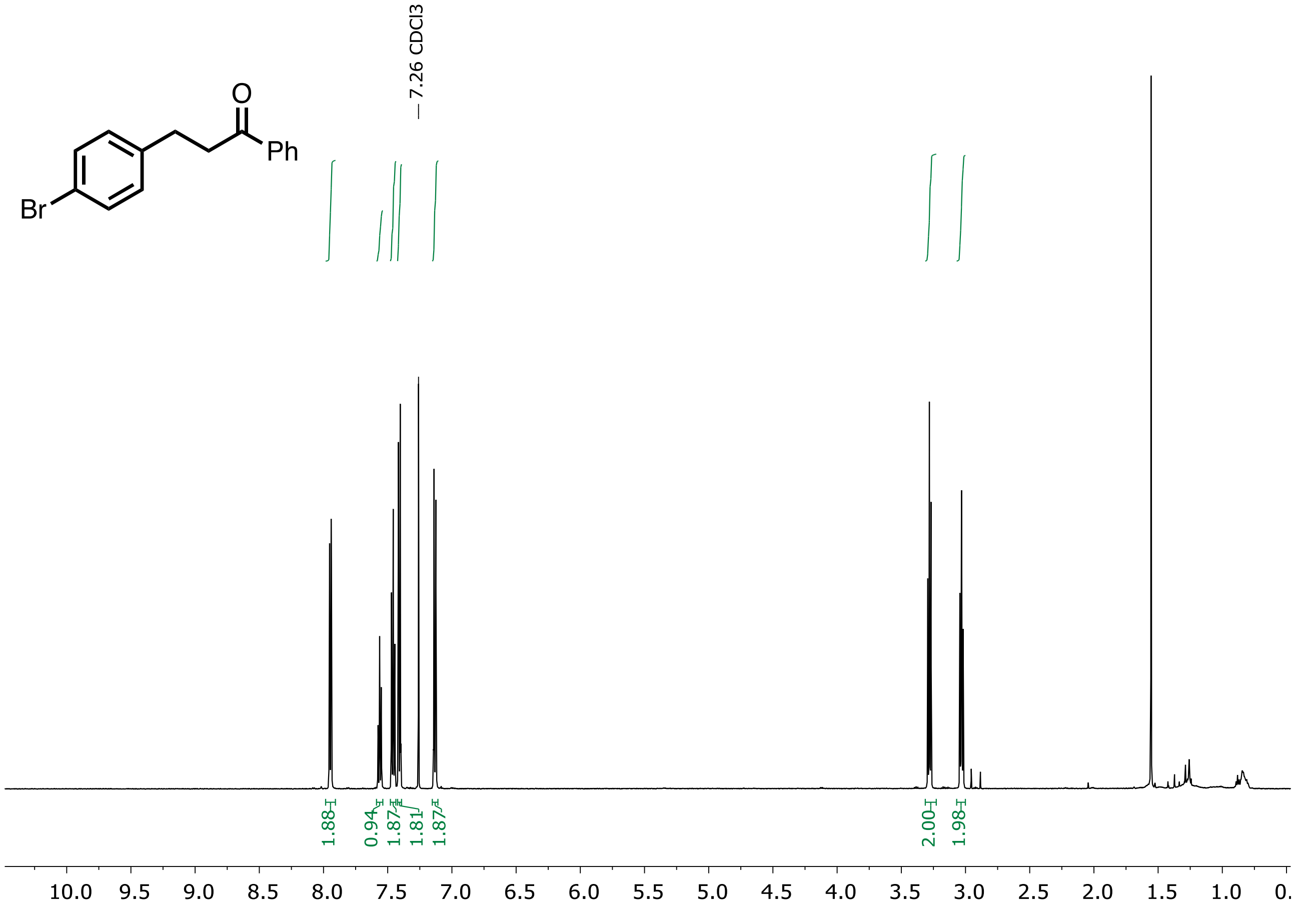

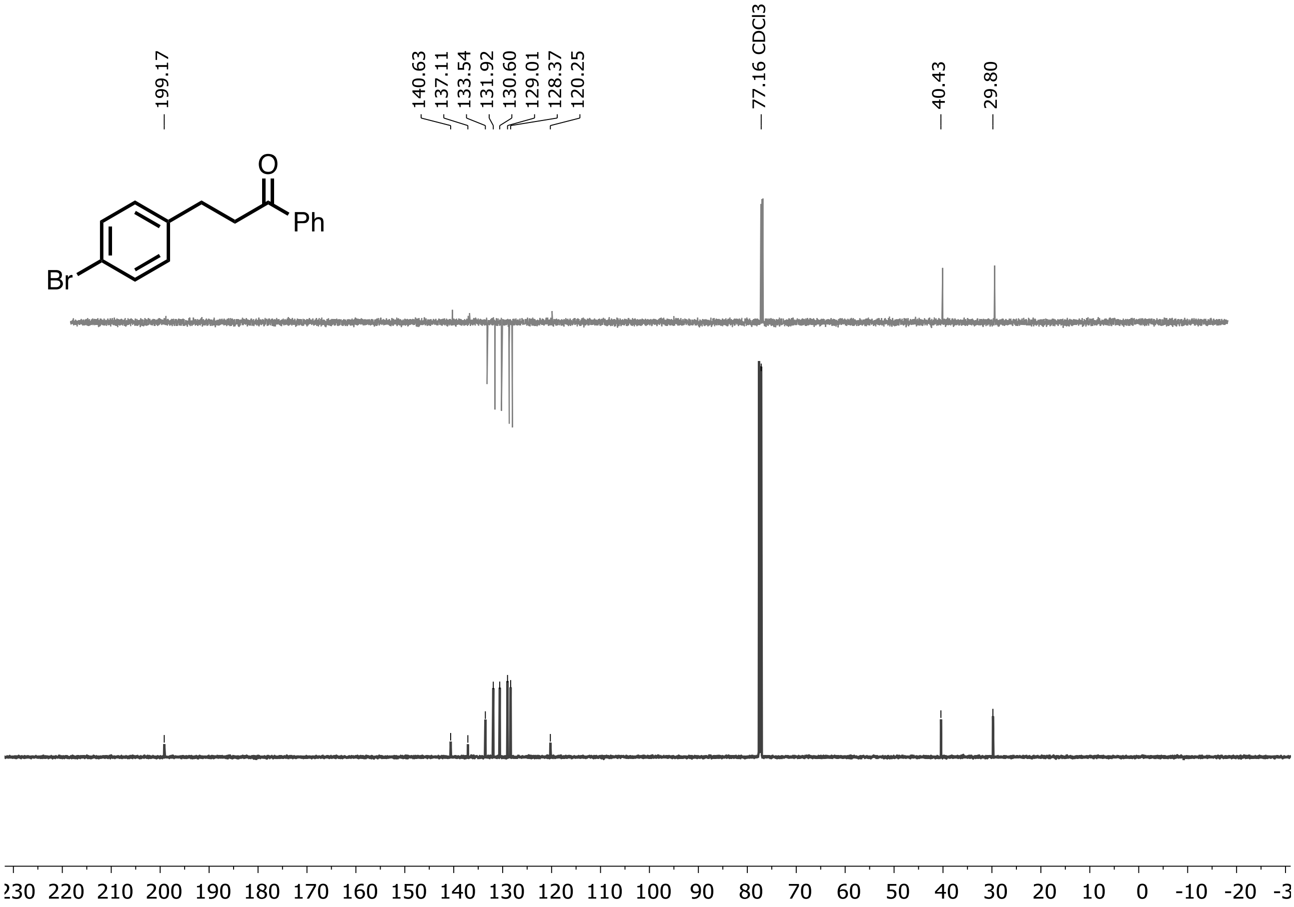

1-Phenyl-3-(4-(ethynyl)phenyl)propan-1-one (1p)

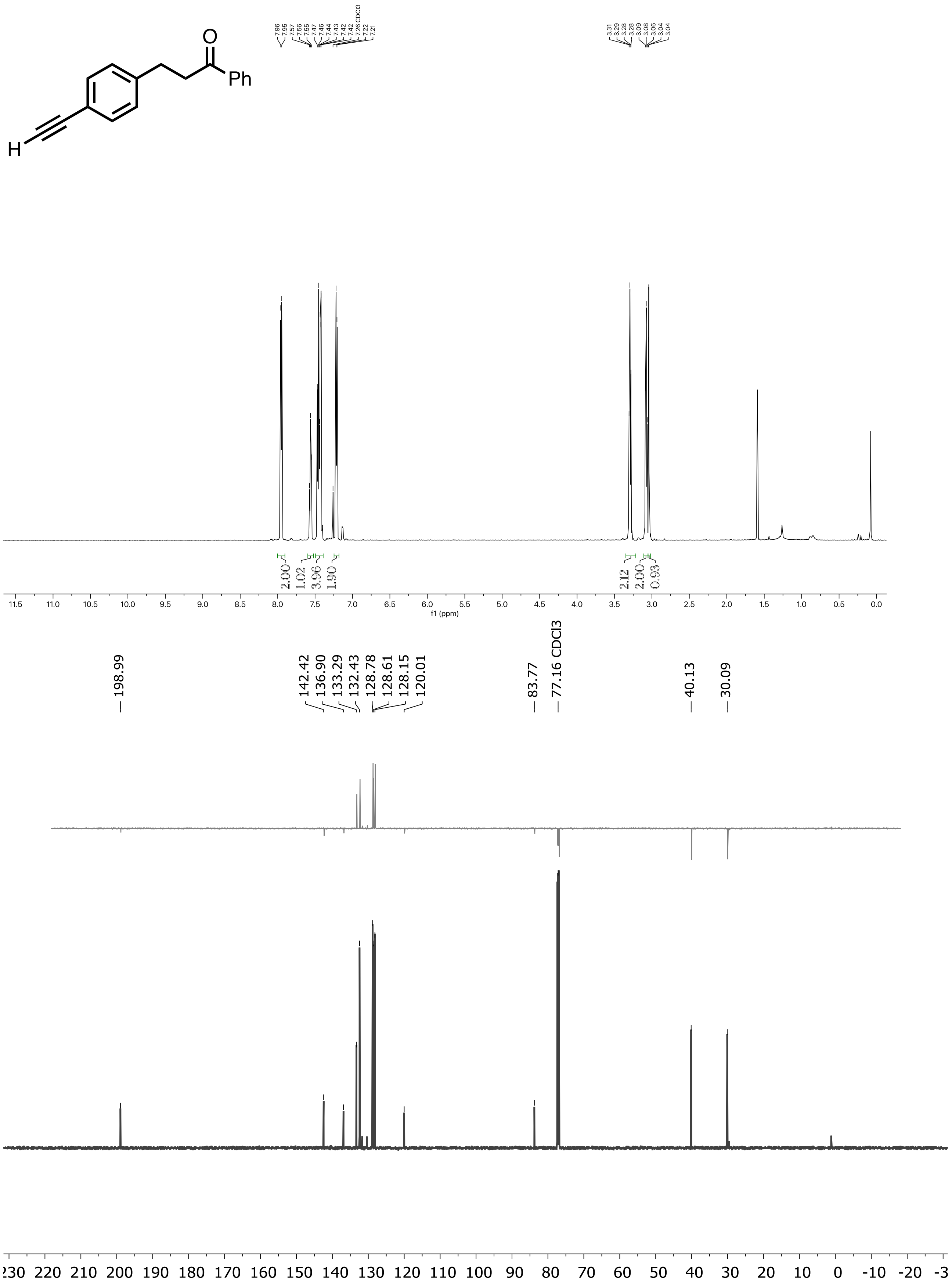

5-Hydroxy-1-phenylpentan-1-one (2a)

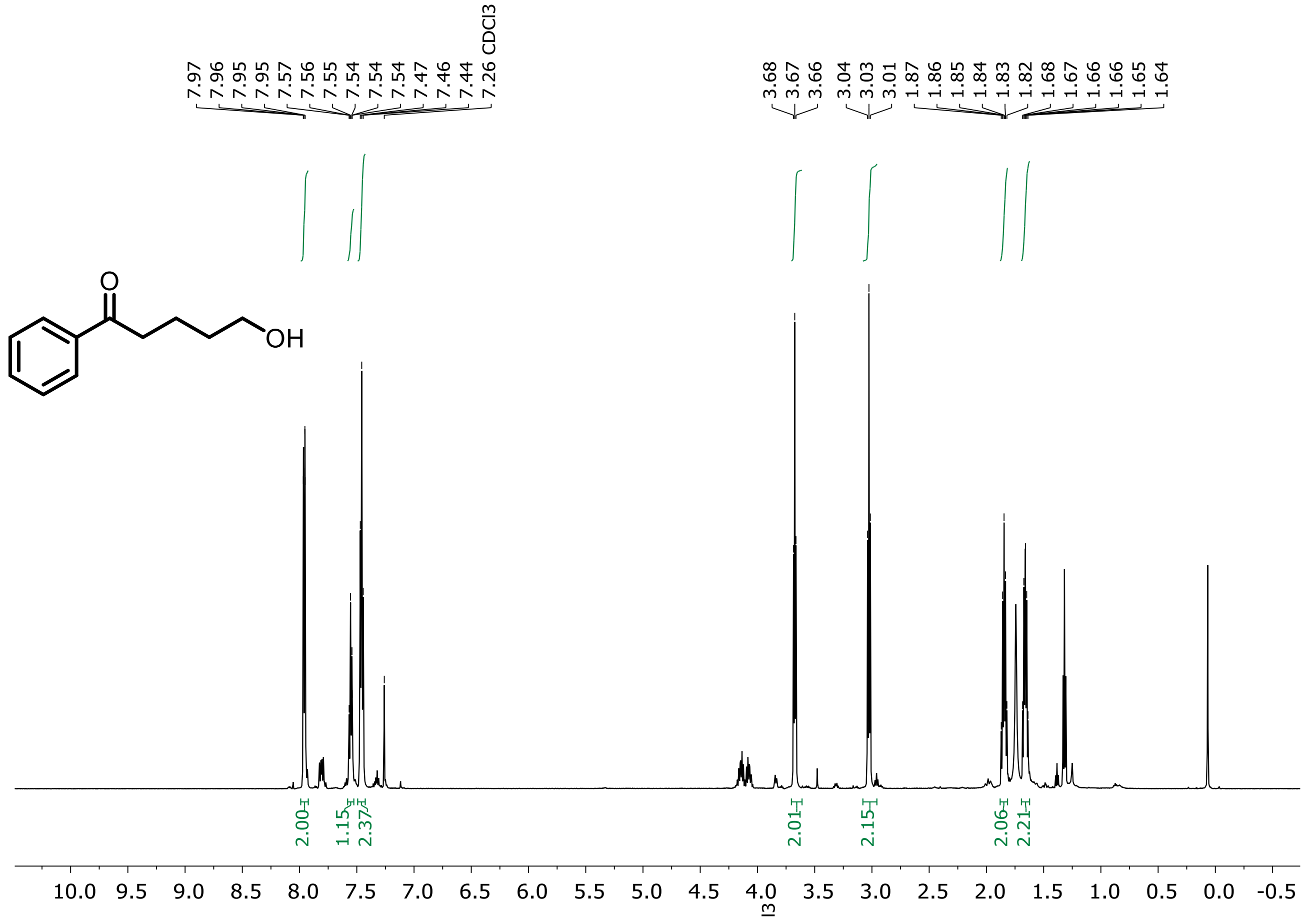

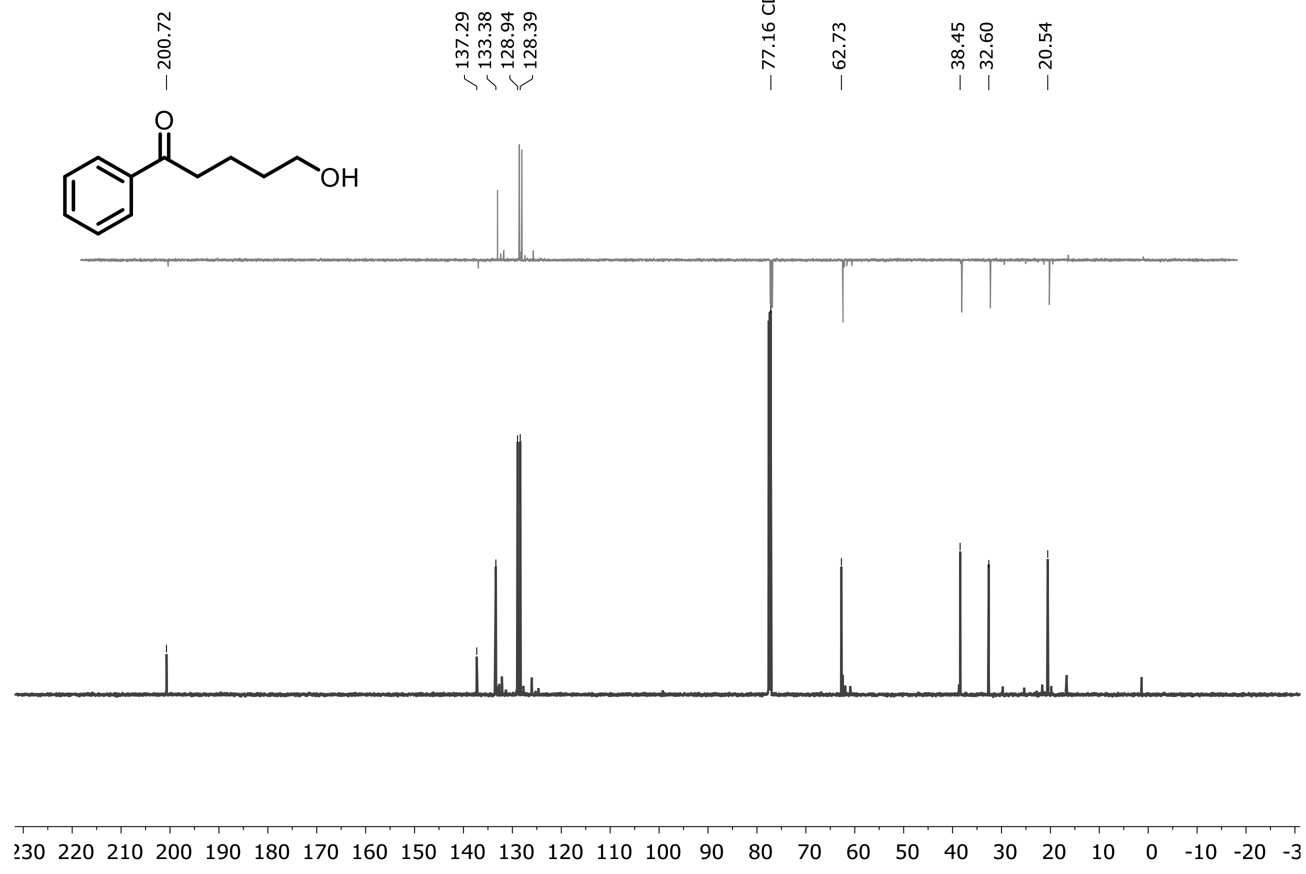

5-Hydroxy-1-(p-tolyl)pentan-1-one (2b)

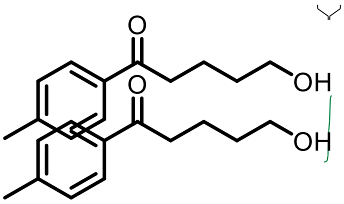

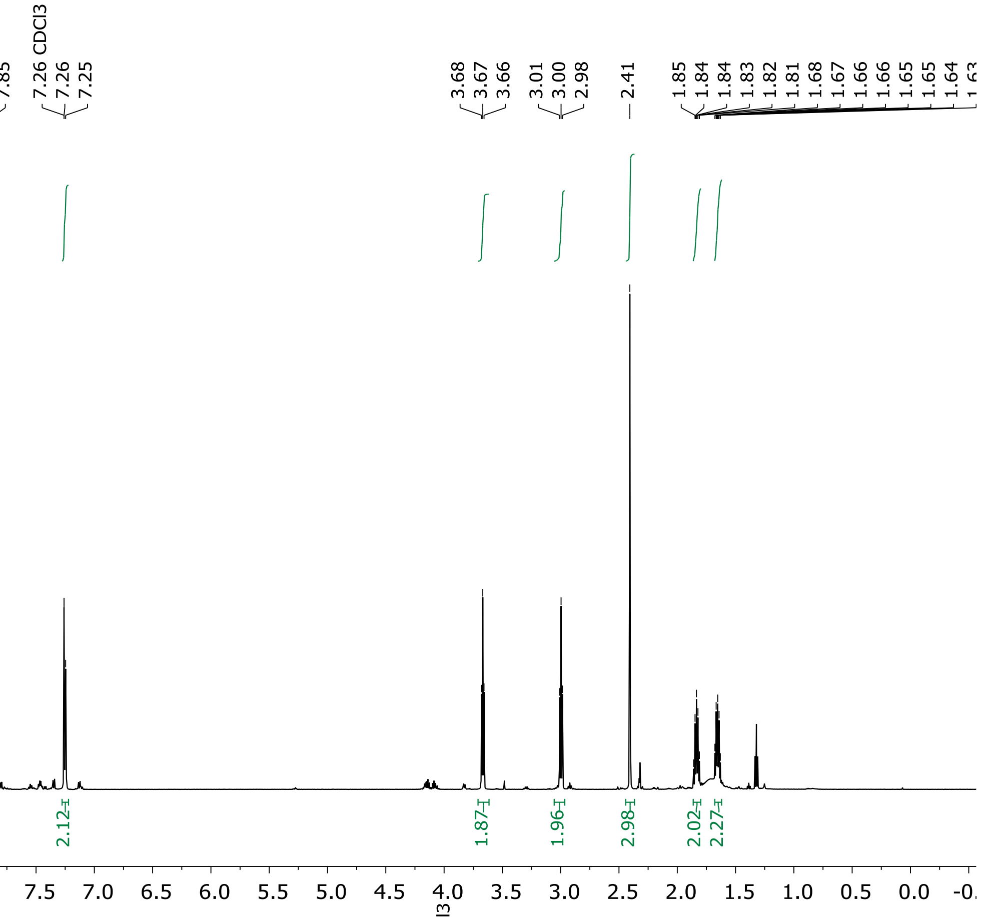

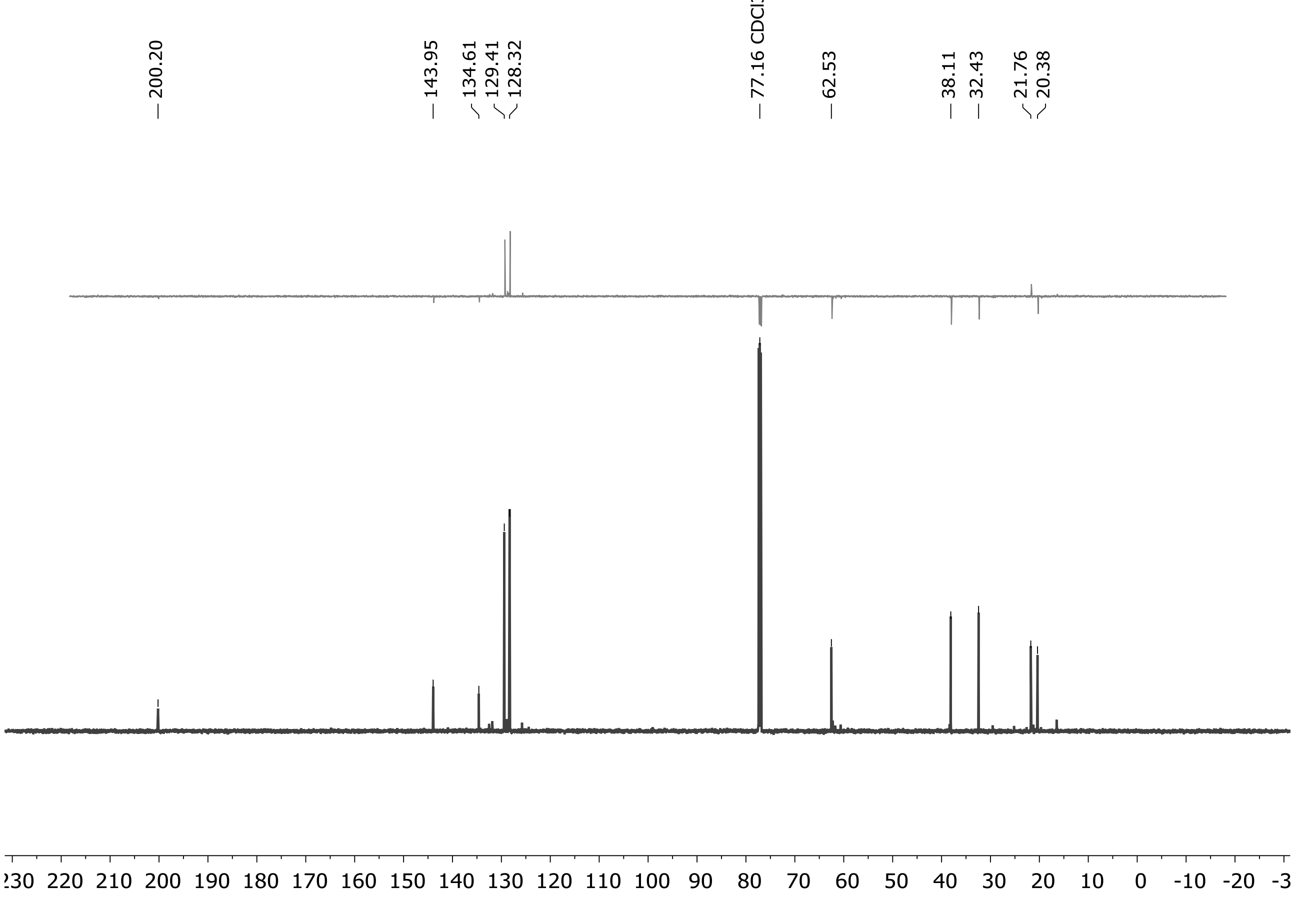

1-(4-Fluorophenyl)-5-hydroxypentan-1-one (2c)

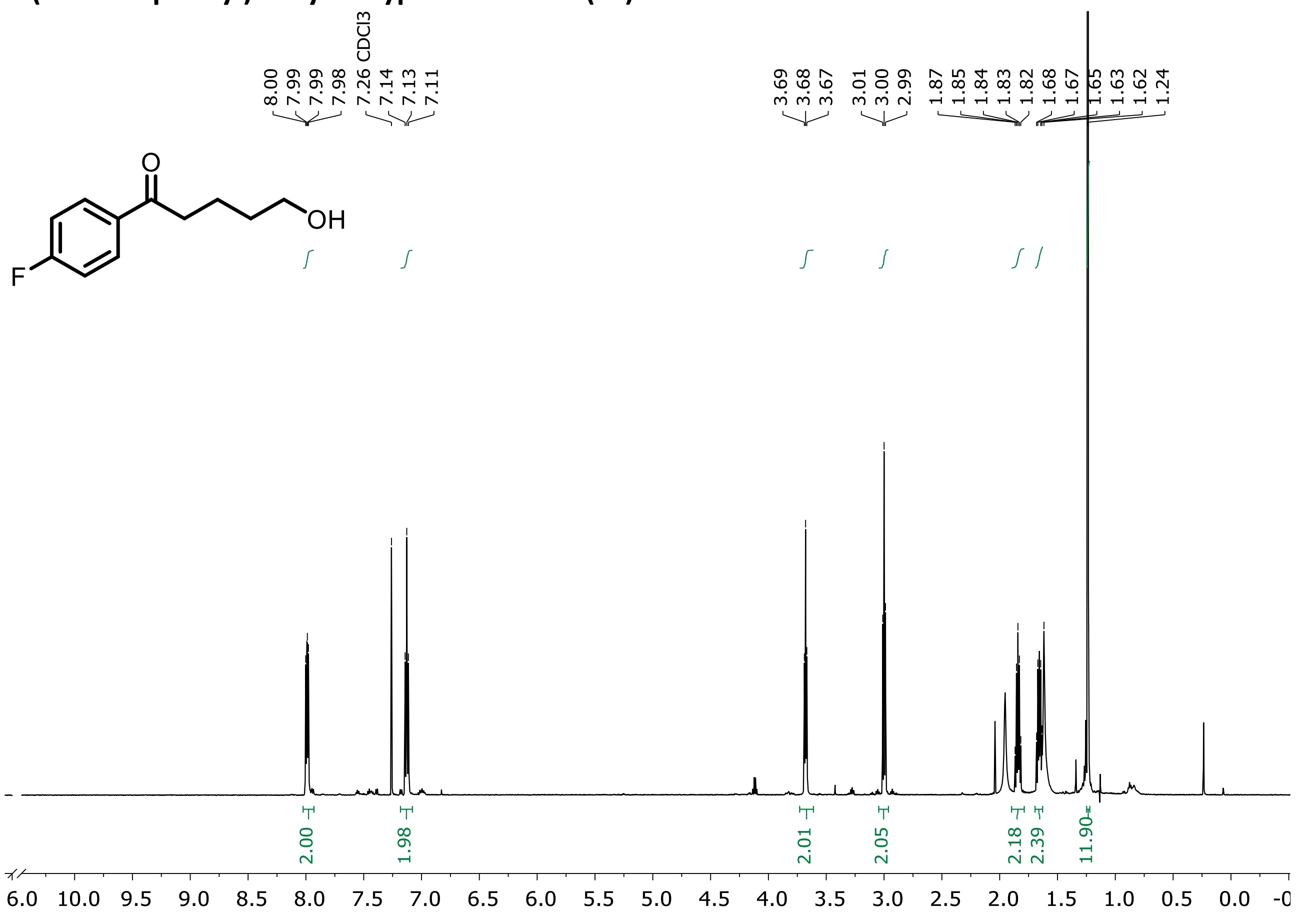

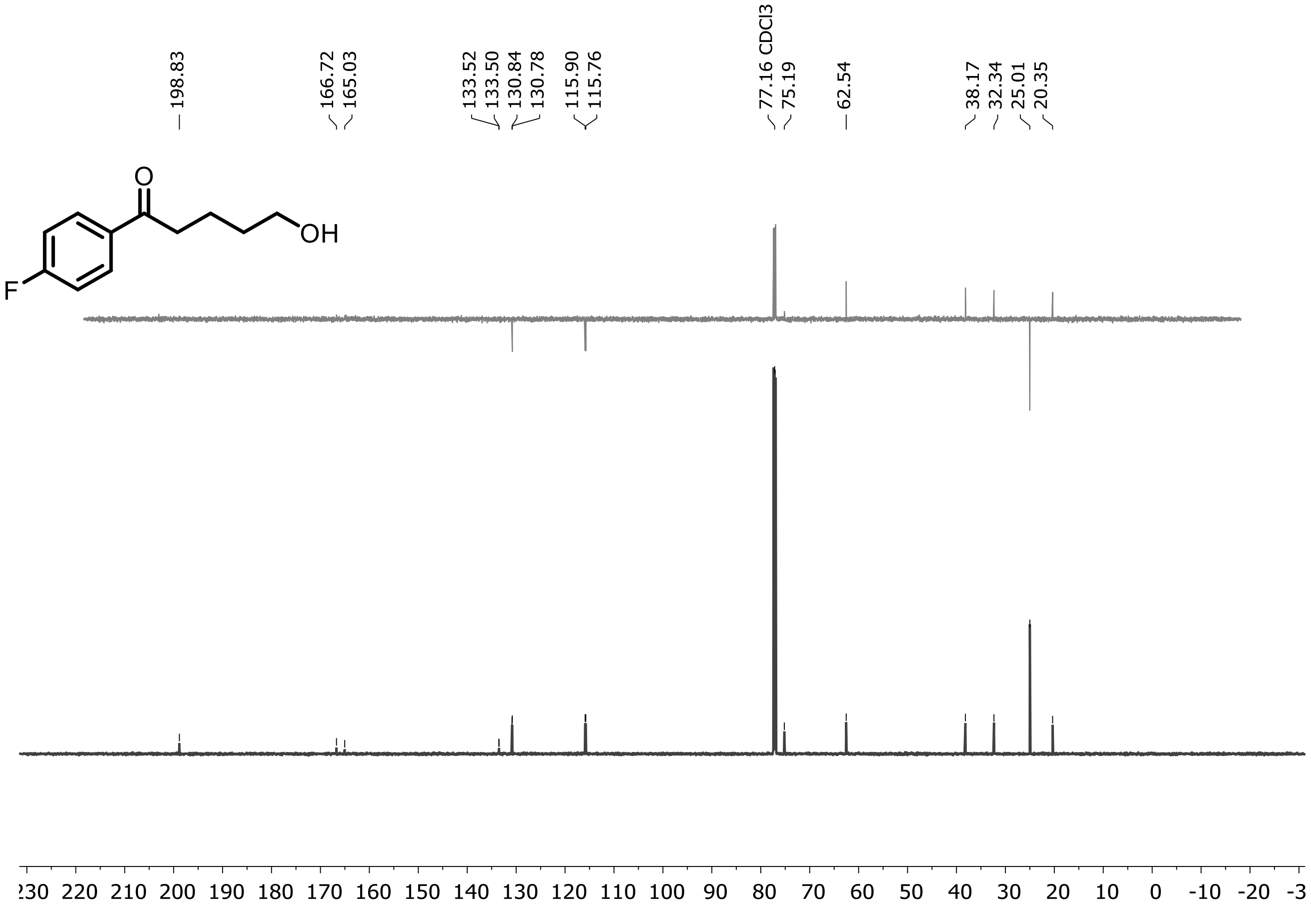

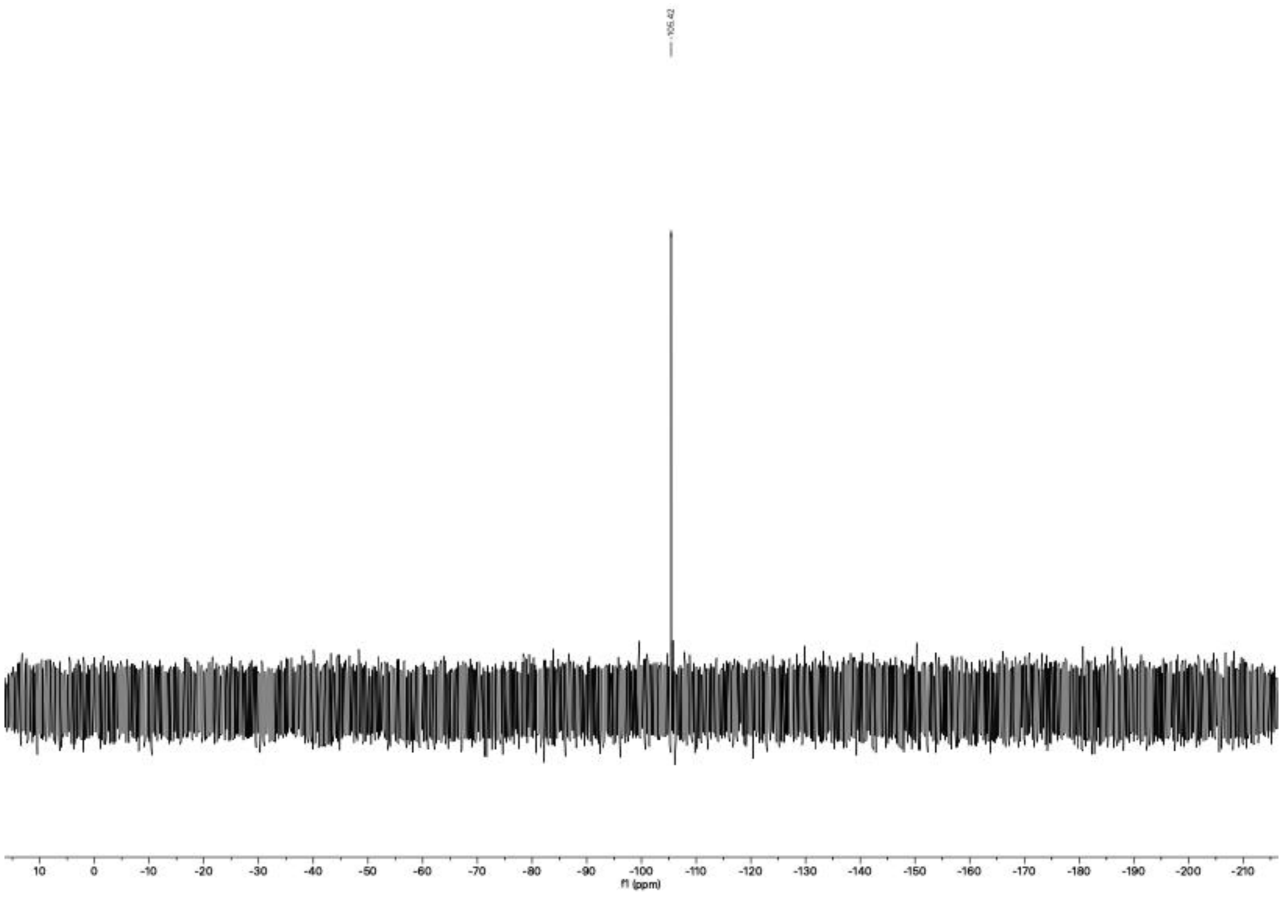
-105.42
10
0
-10
-20
-30
-40
-50
-60
-70
-80
-90
-100
-110
-120
-130
-140
-150
-160
-170
-180
-190
-200
-210
f1 (ppm)

1-(4-Chlorophenyl)-5-hydroxypentan-1-one (2d)

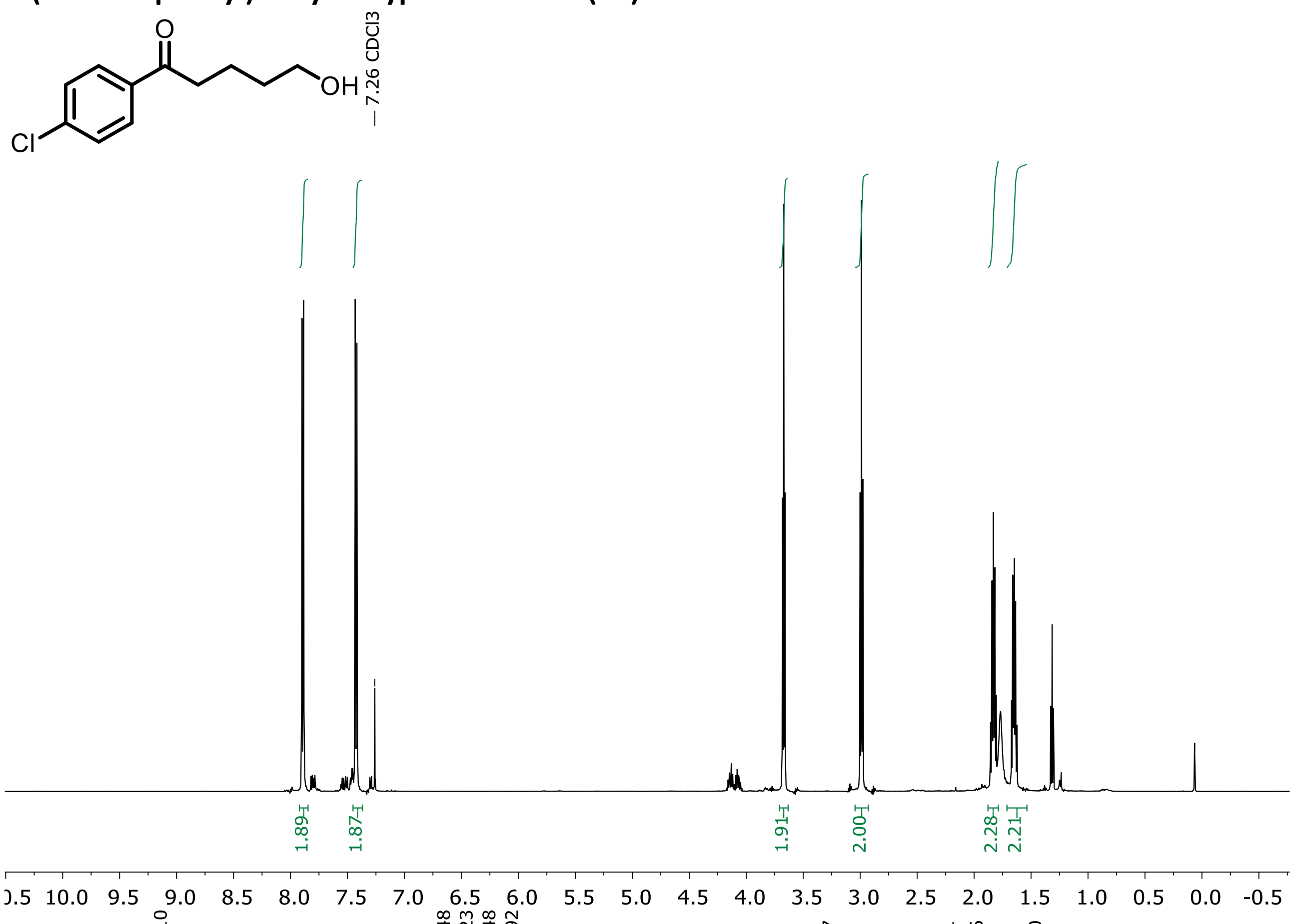

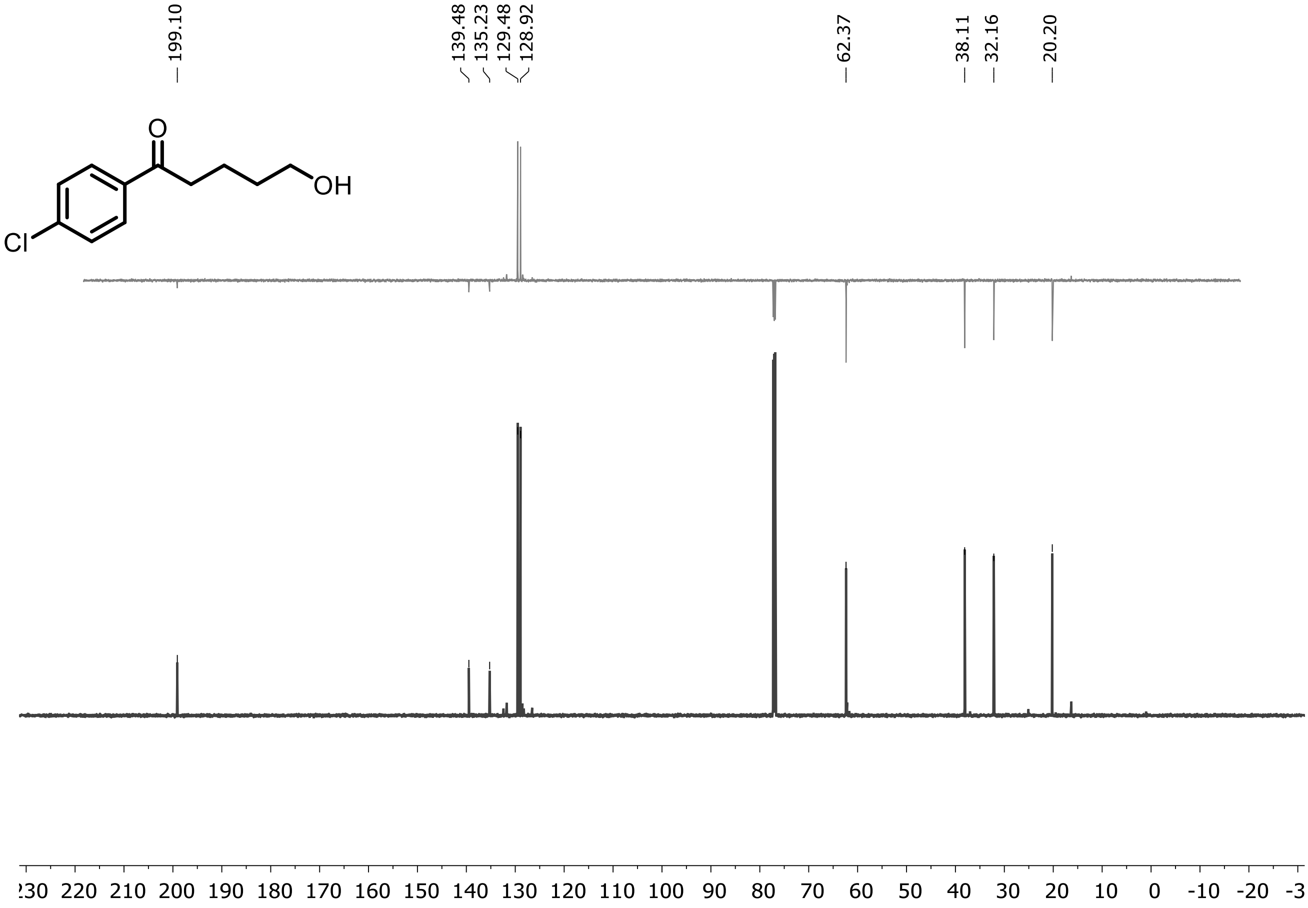

1-(4-Bromophenyl)-5-hydroxypentan-1-one (2e)

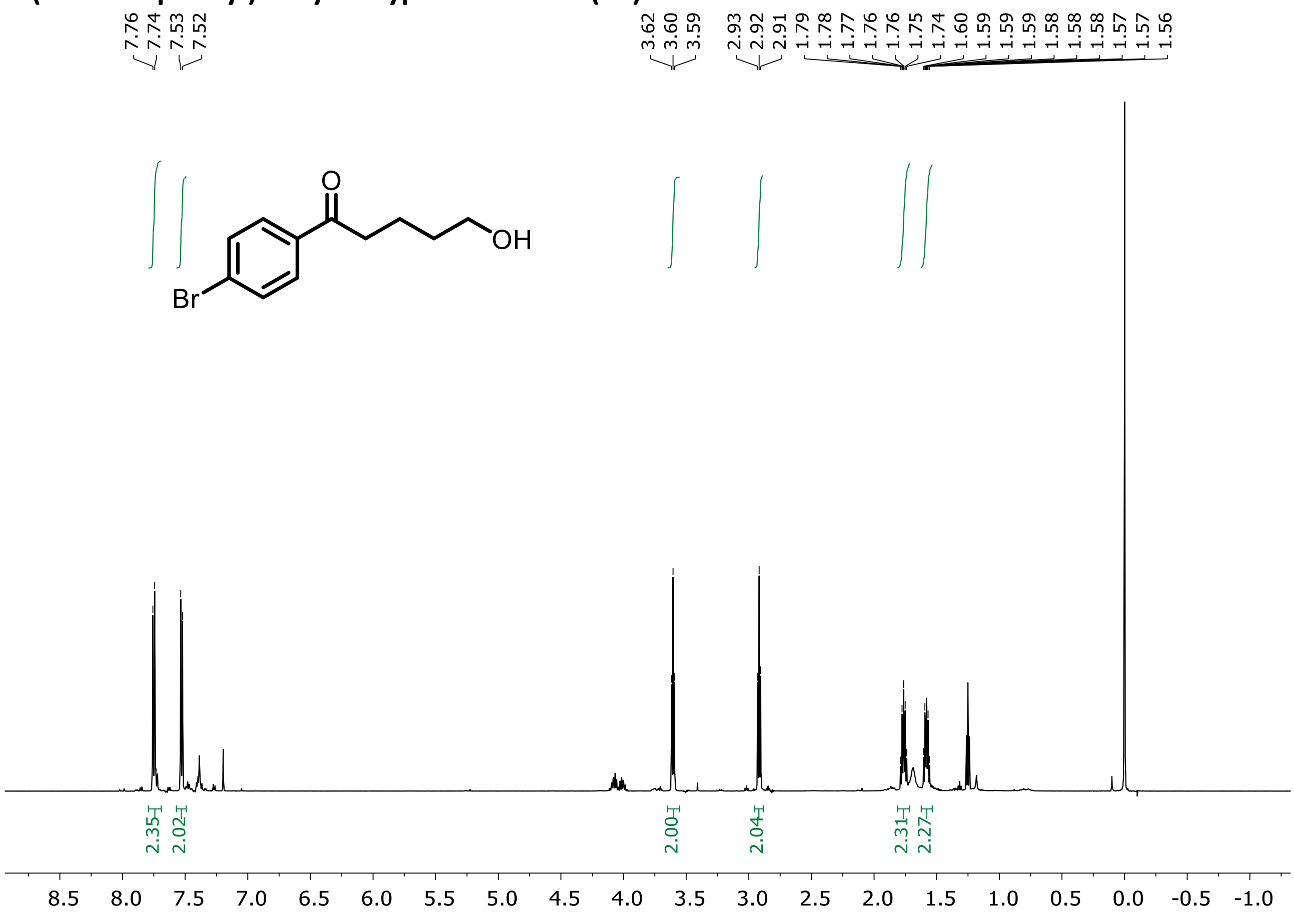

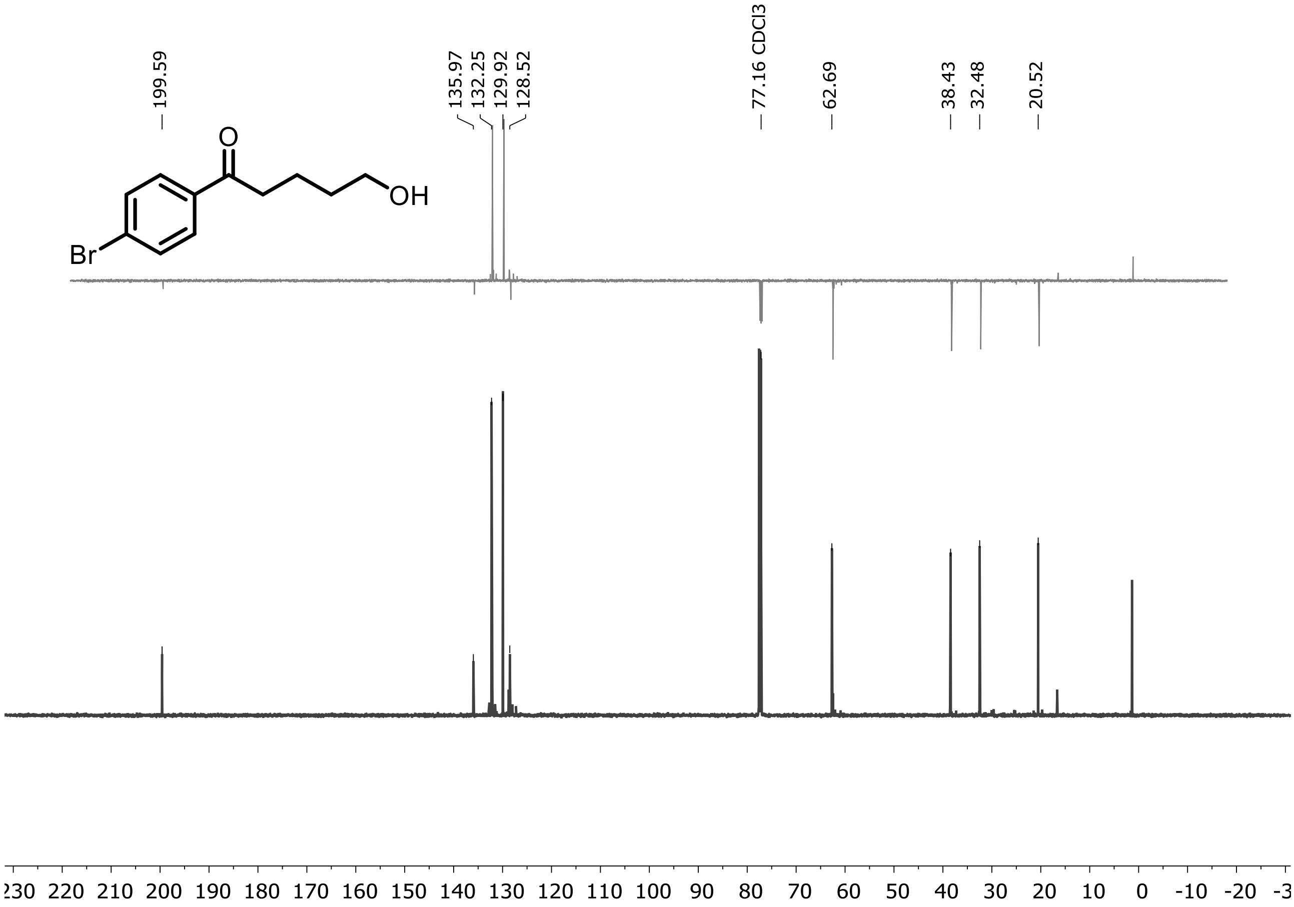

5-Hydroxy-1-(4-methoxyphenyl)-1-pentanone (2f)

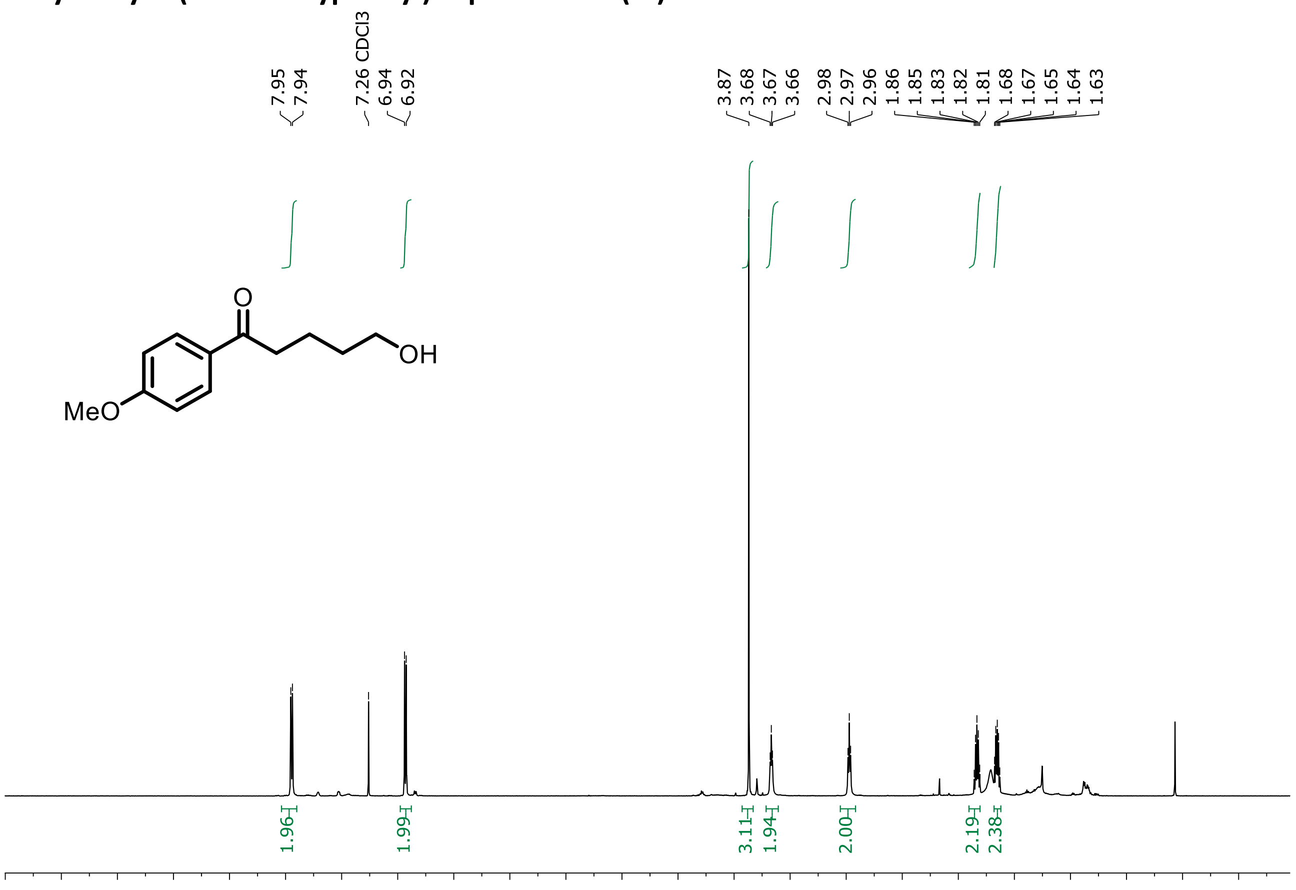

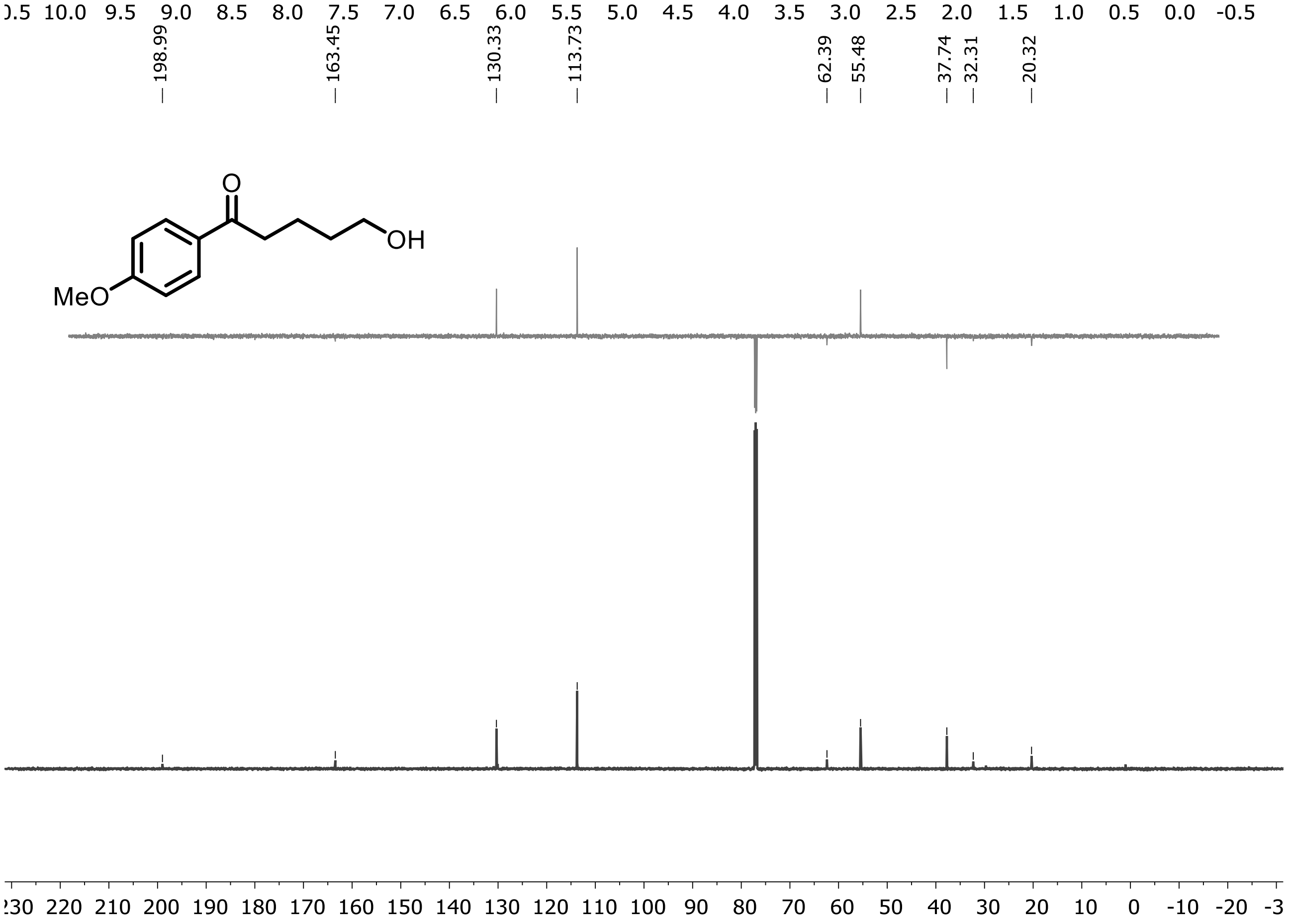

6-Hydroxy-1-phenylhexan-1-one (2g)

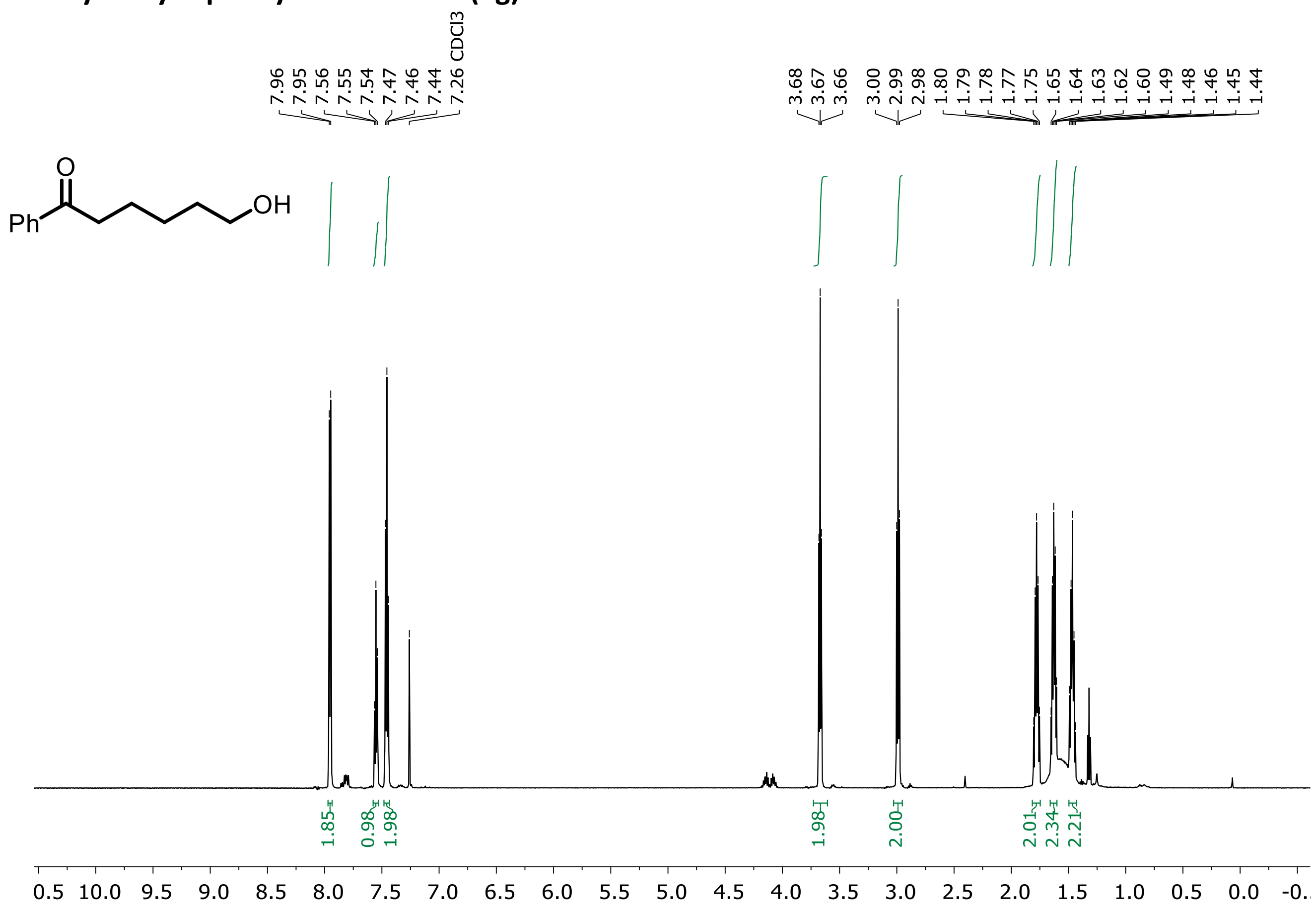

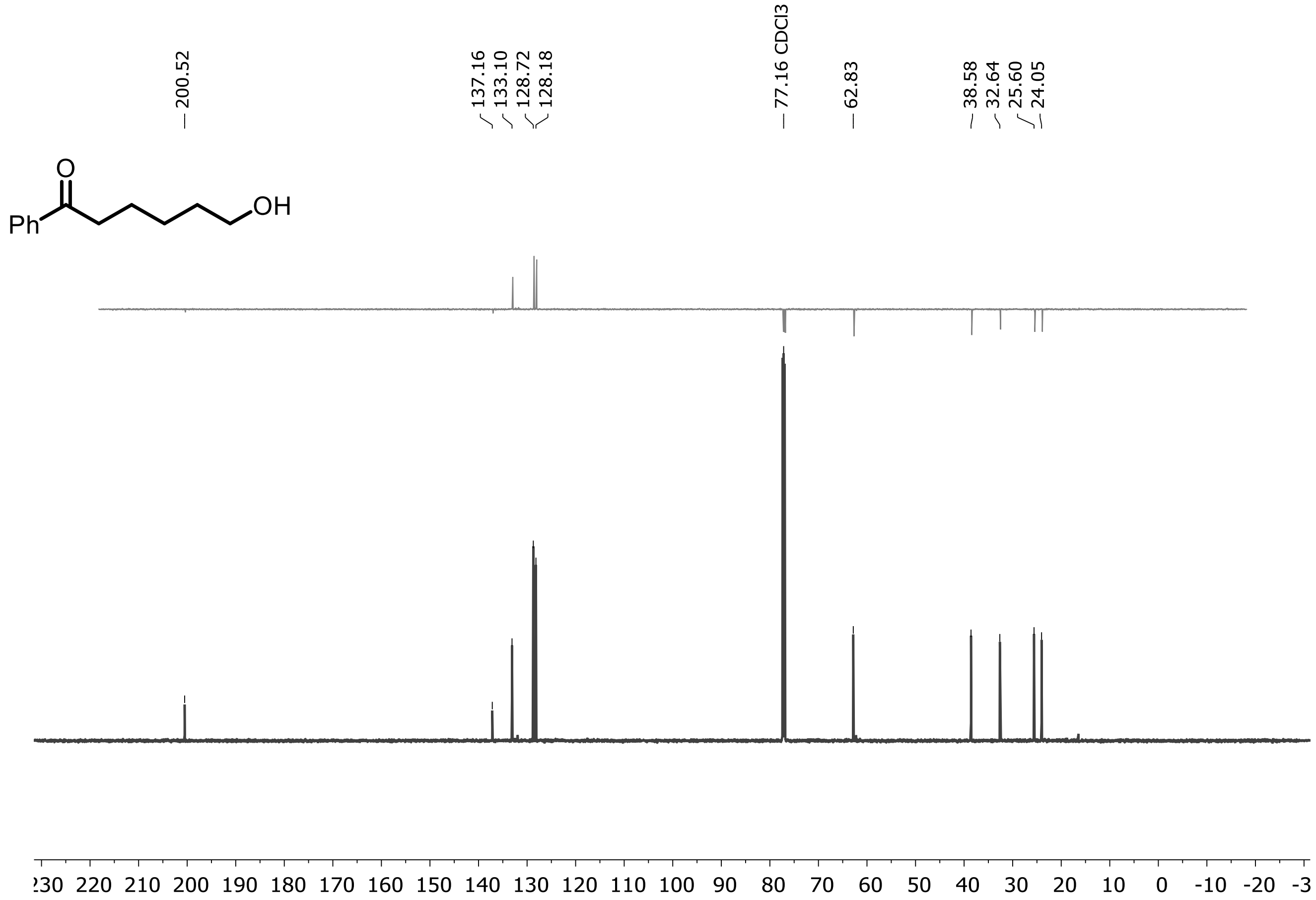

7-Hydroxy-1-phenylheptan-1-one (2h)

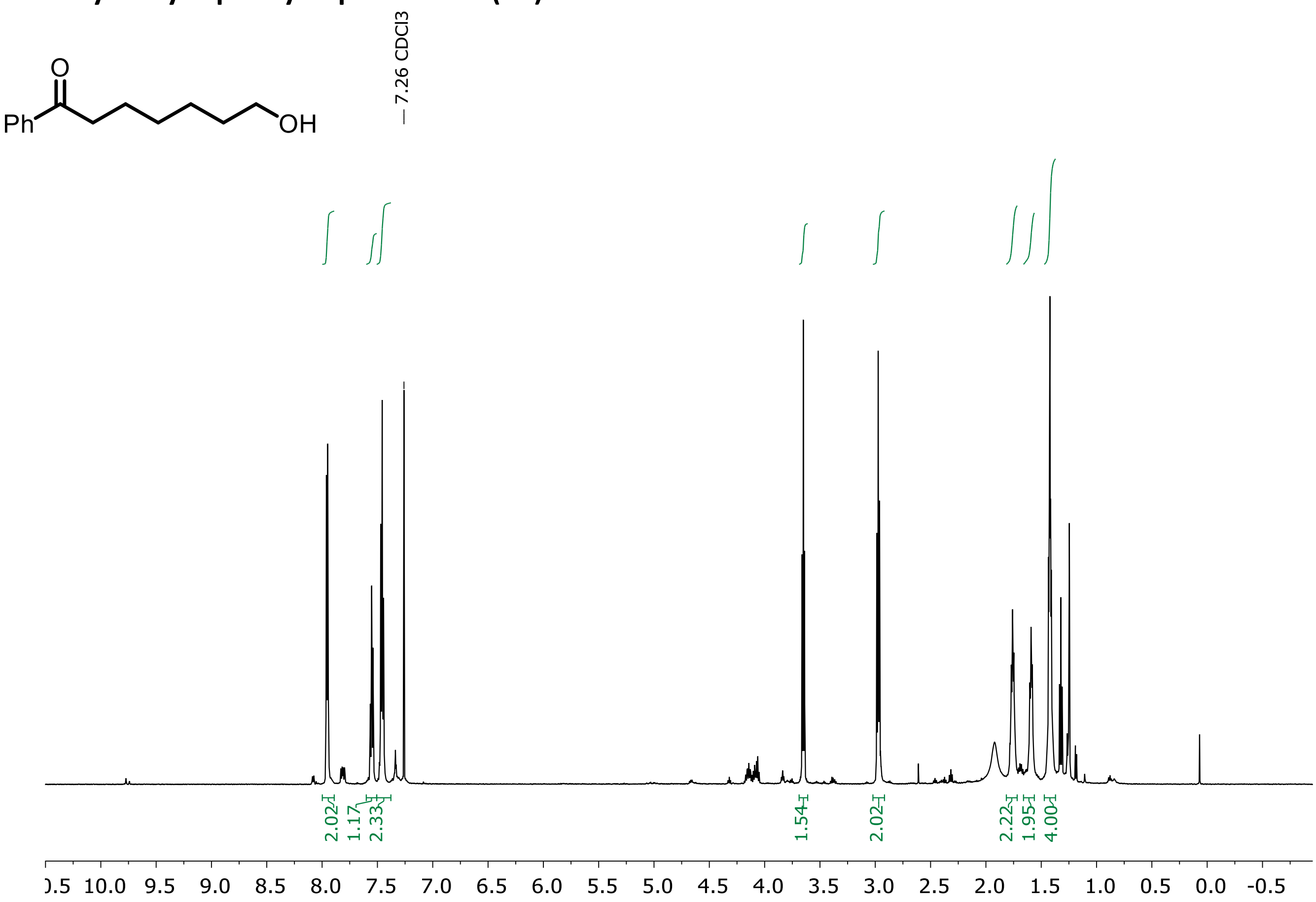

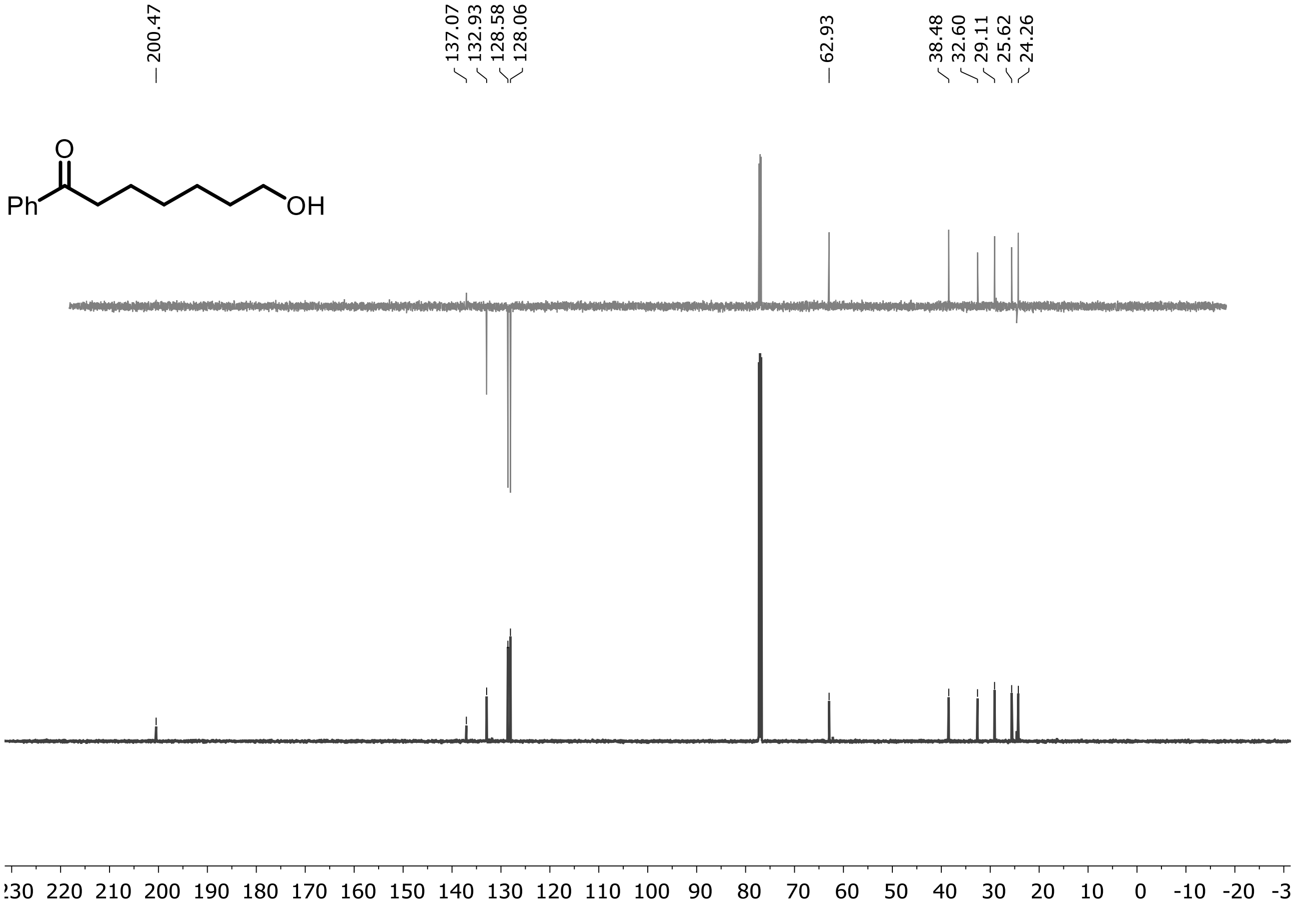

8-Hydroxy-1-phenyloctan-1-one (2i)

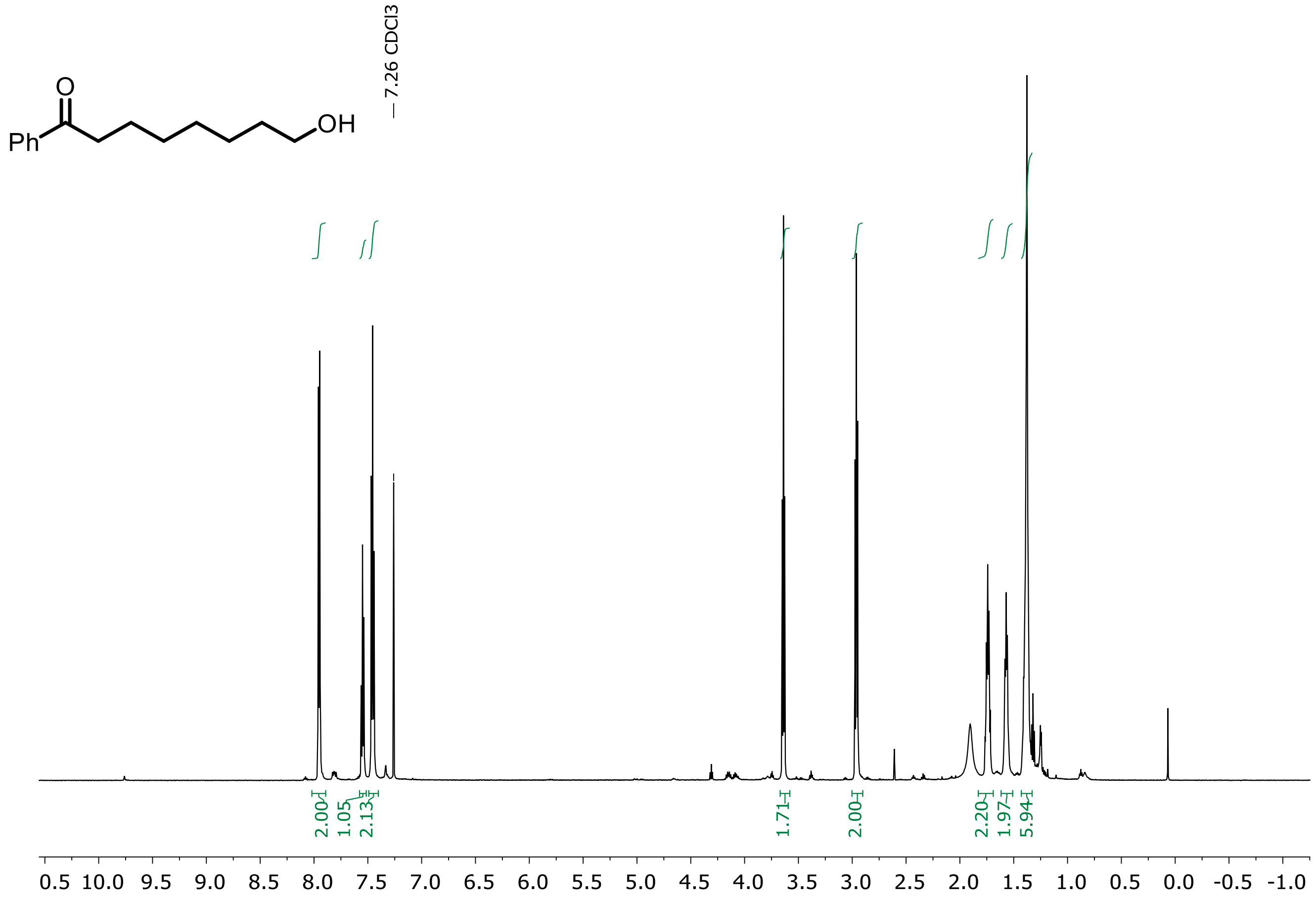

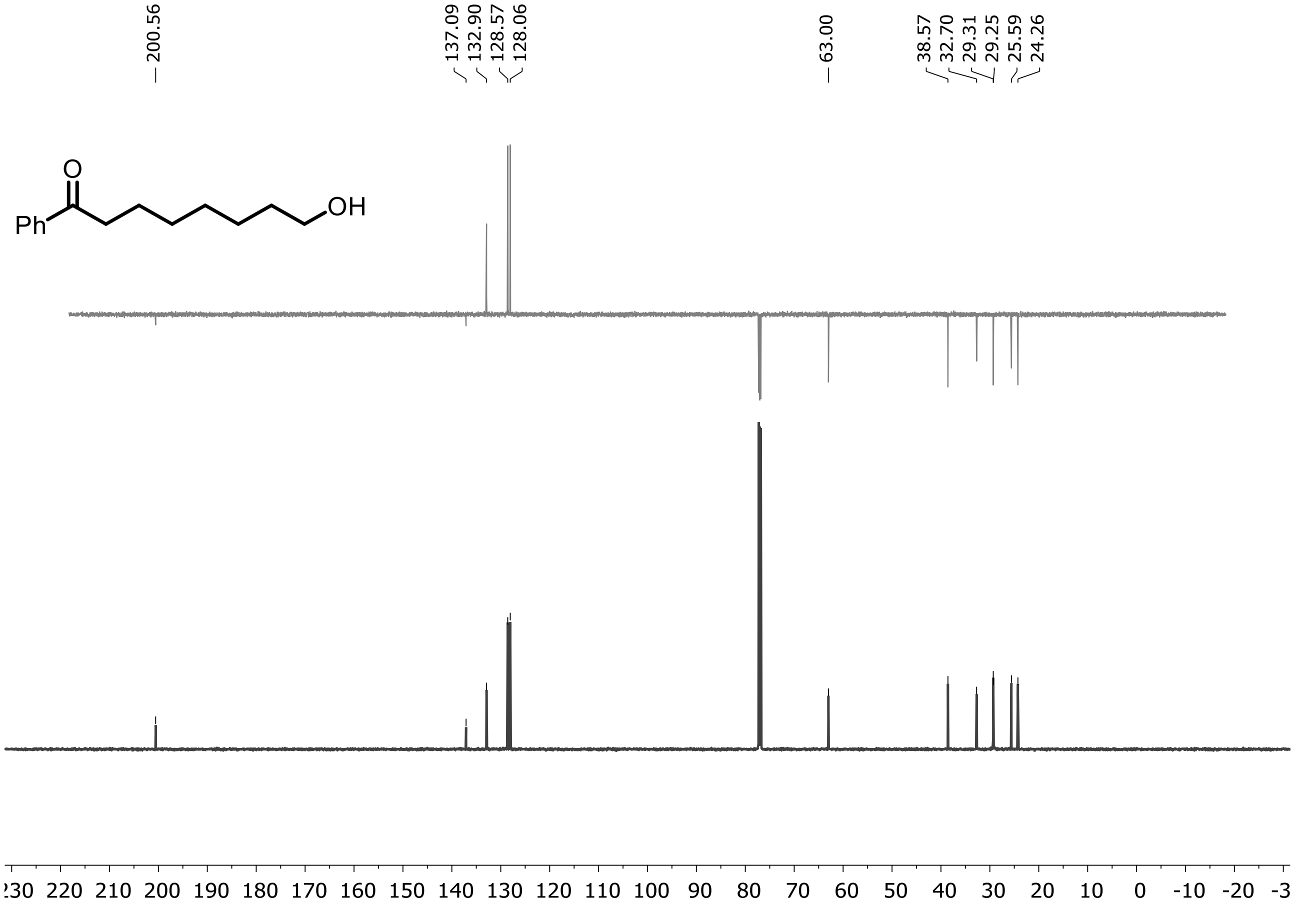

10-Hydroxytetradecanoic acid (2j)

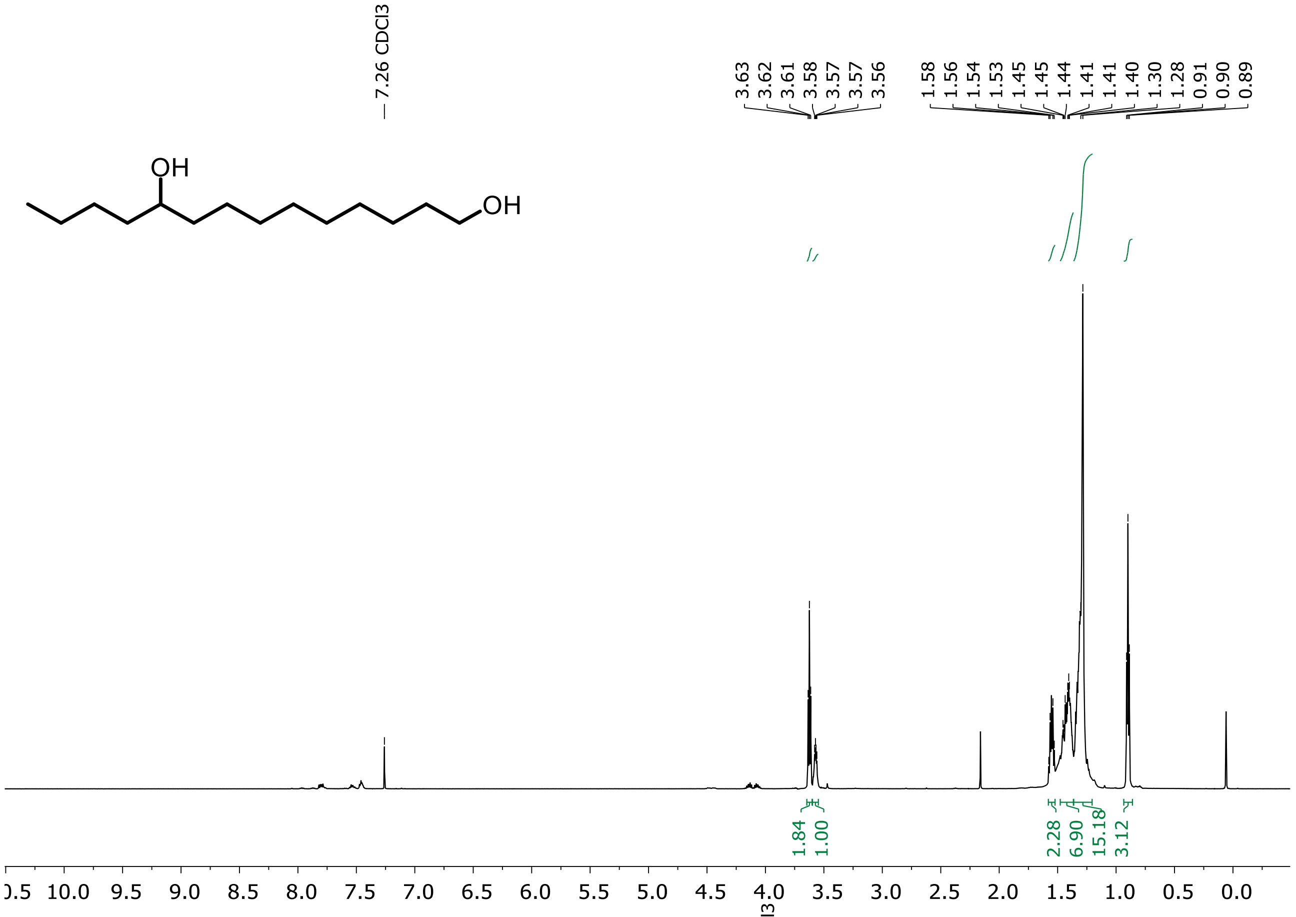

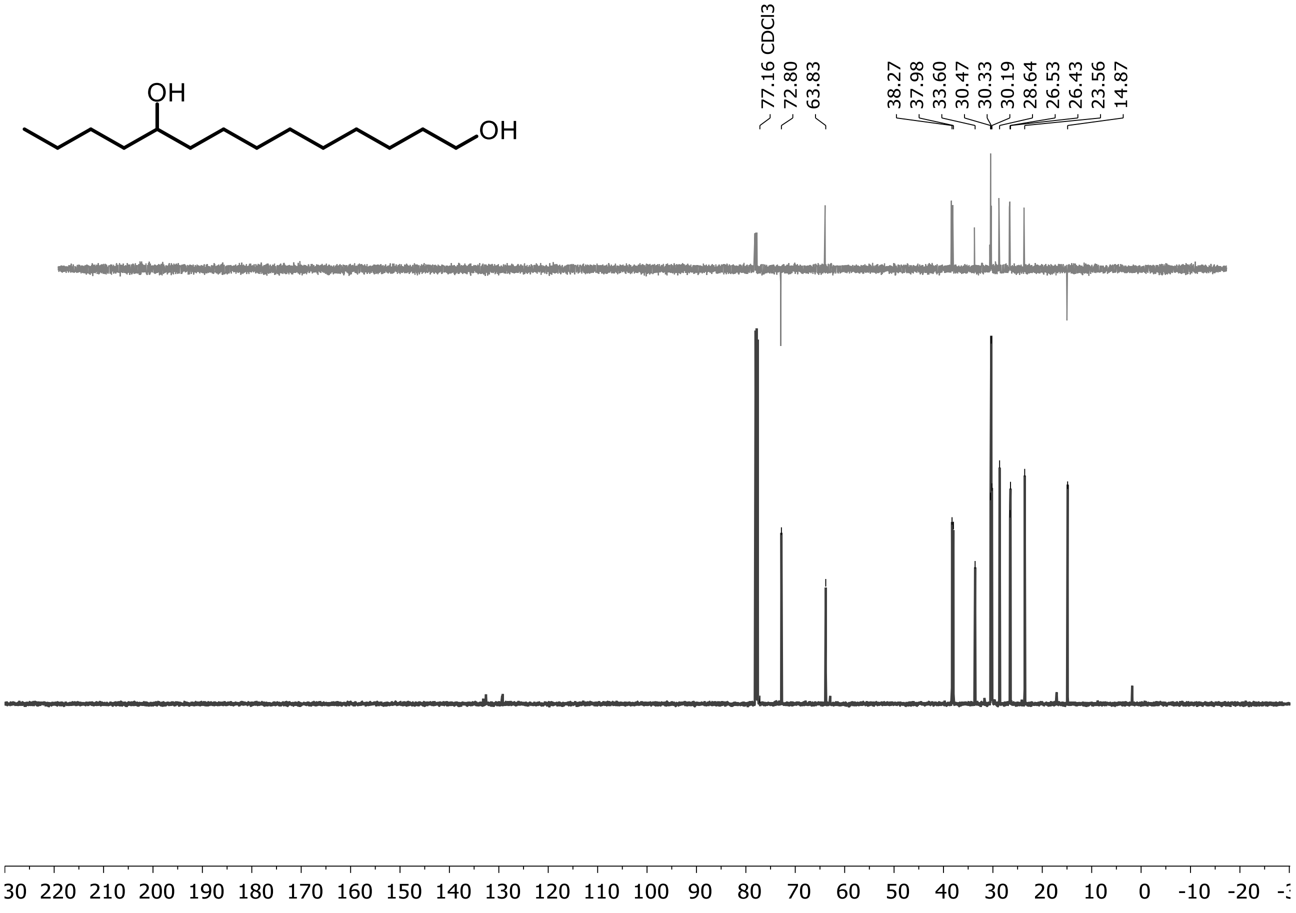

2-(2-Hydroxyethyl)dibenzo[b,e]oxepin-11(6*H*)-one (2k)

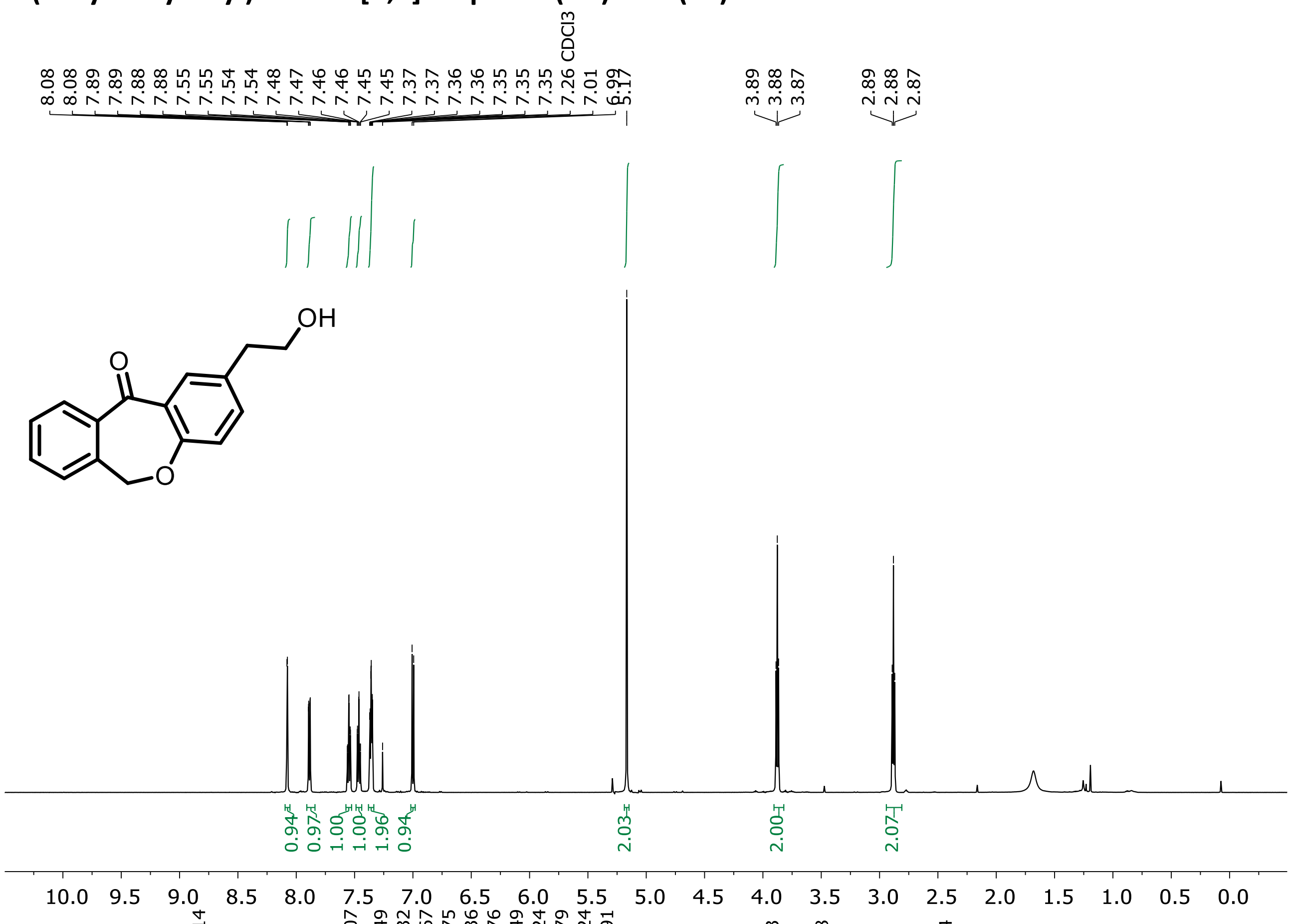

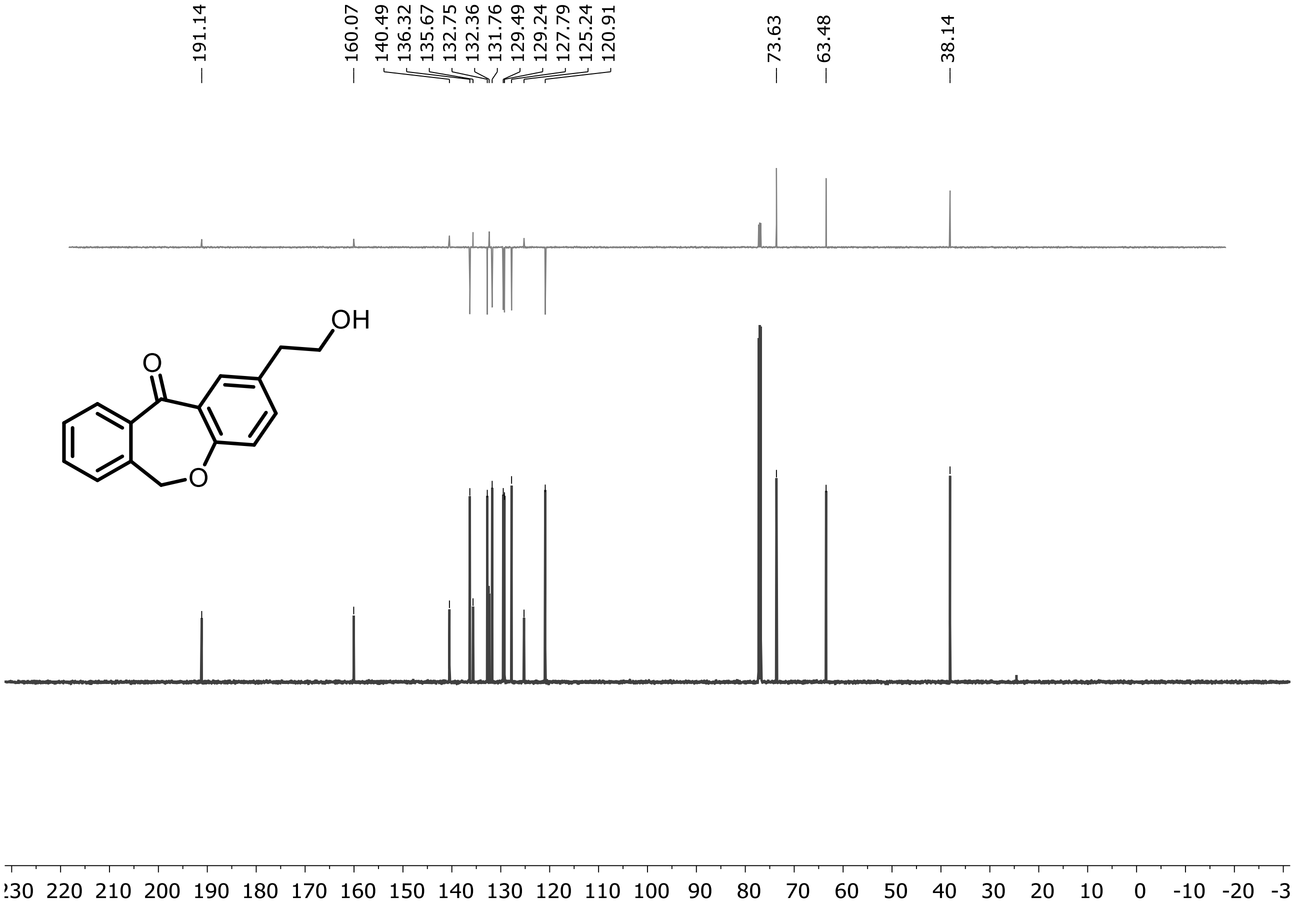

(3-(1-Hydroxypropan-2-yl)phenyl)(phenyl)methanone (2l)

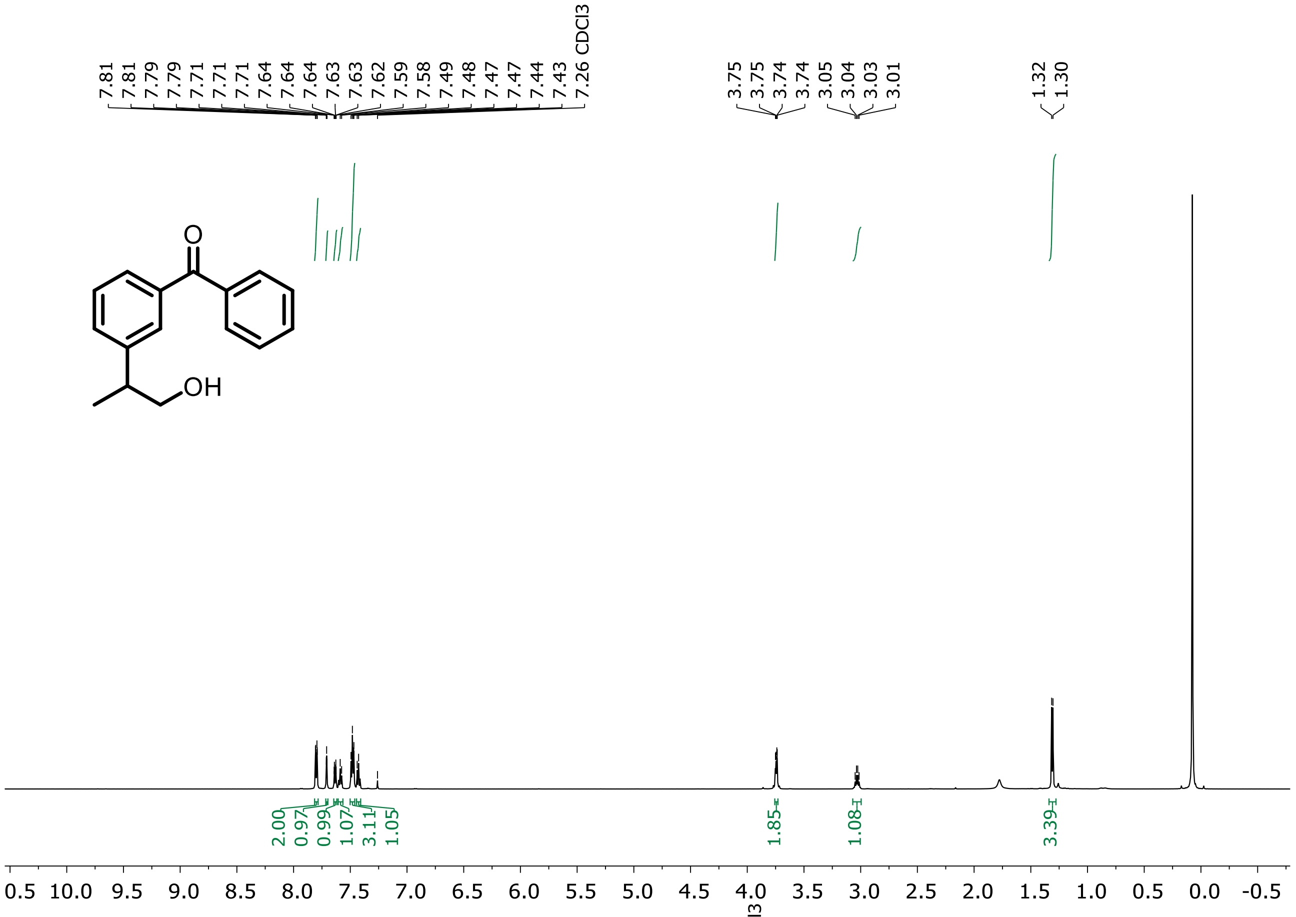

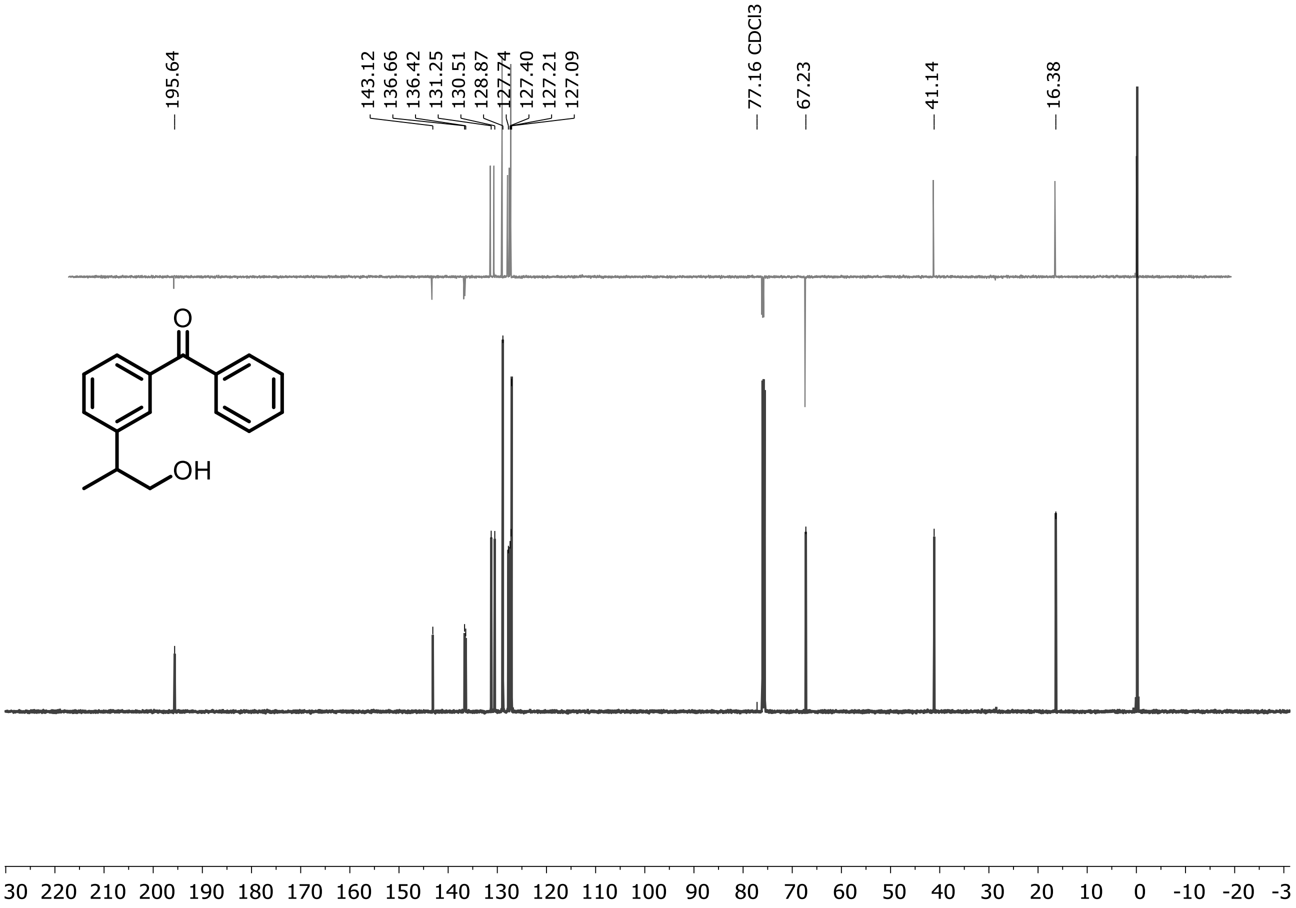

2-(1-Hydroxypropan-2-yl)dibenzo[b,f]thiepin-10(11*H*)-one (2m)

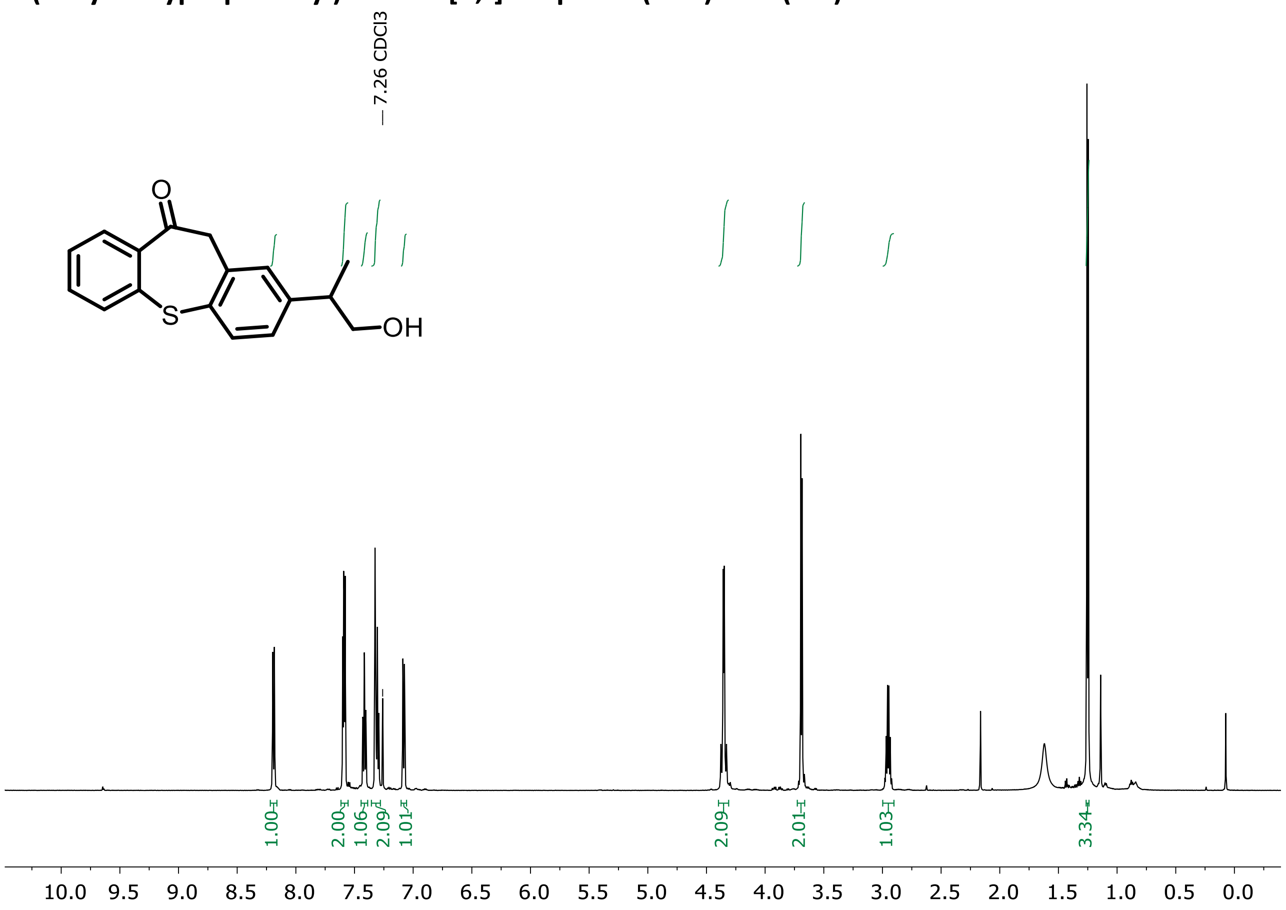

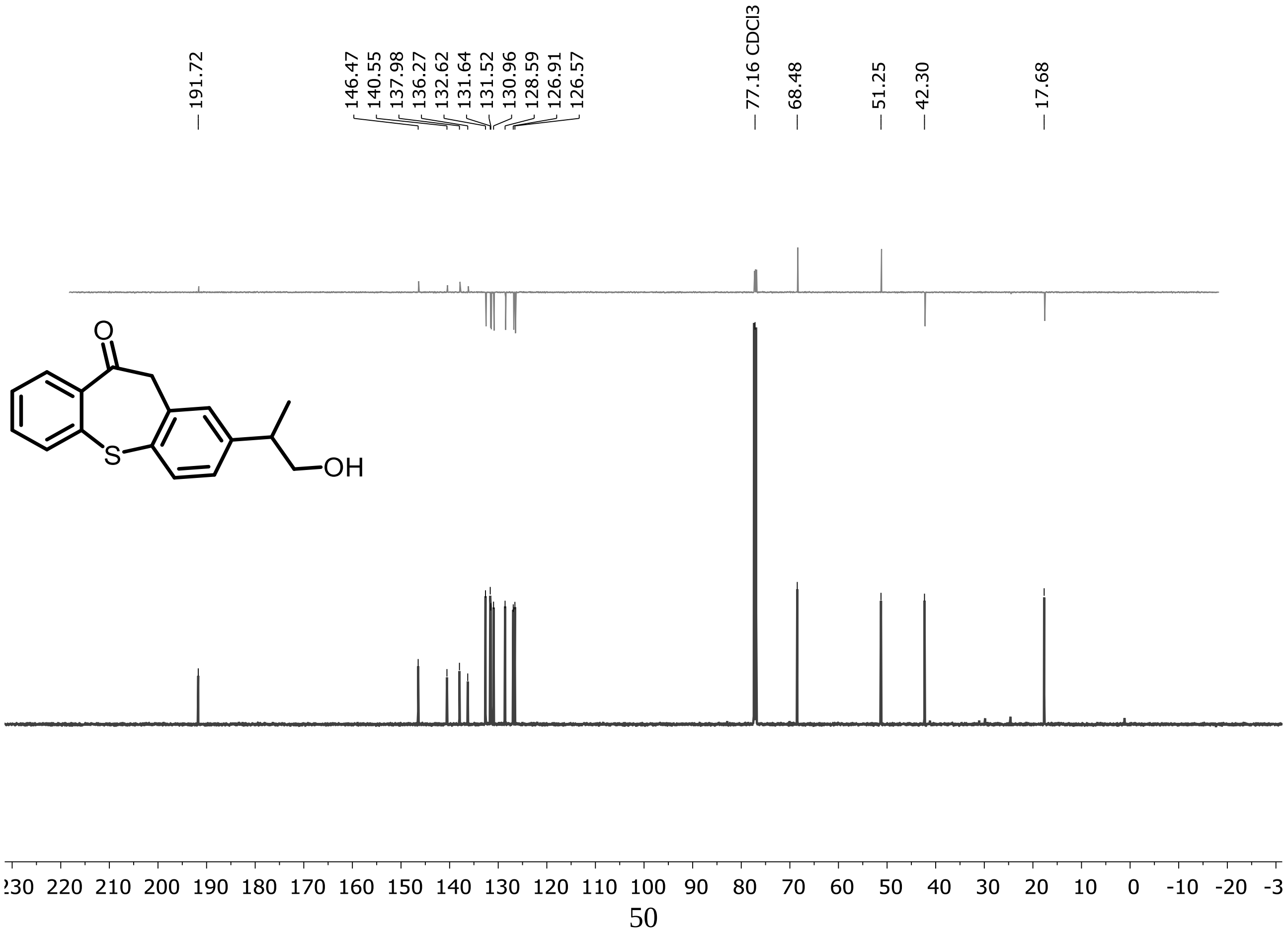

_n_-Pent-4-enyl alcohol (2n)

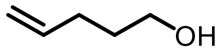

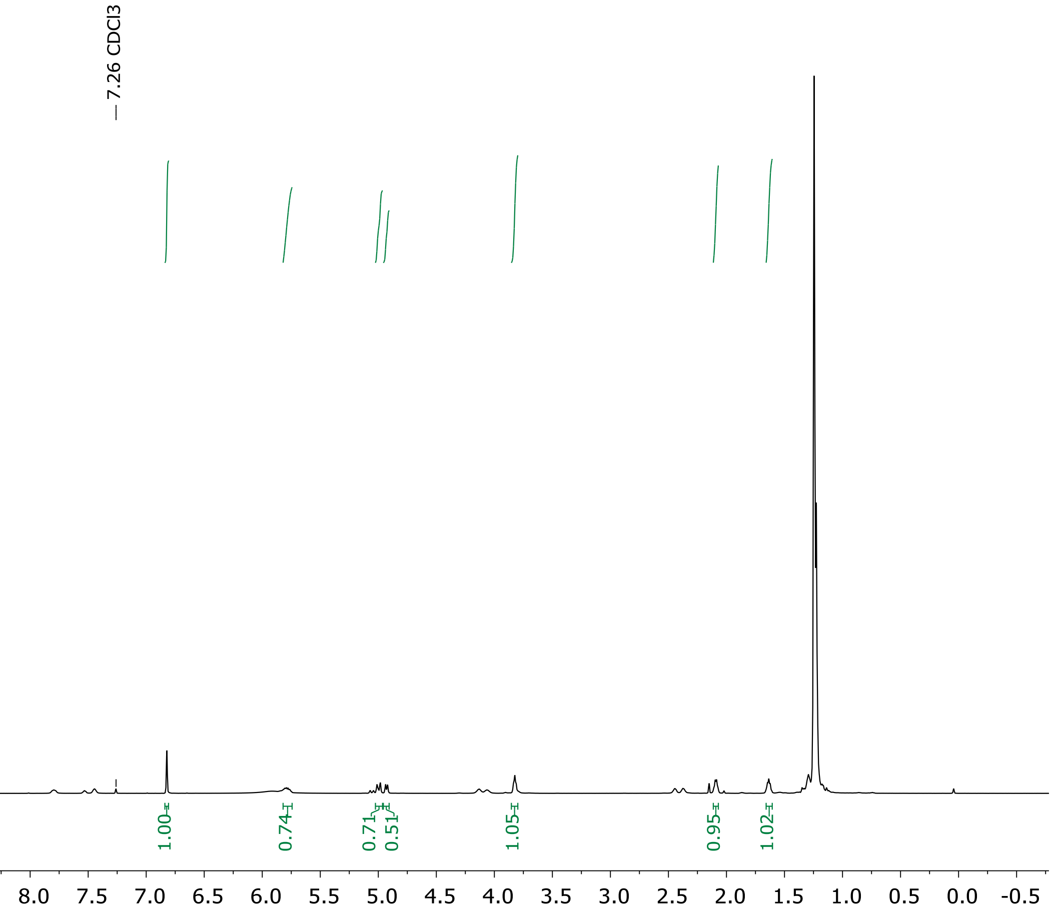

10-Undecen-1-ol (2o)

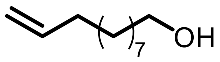

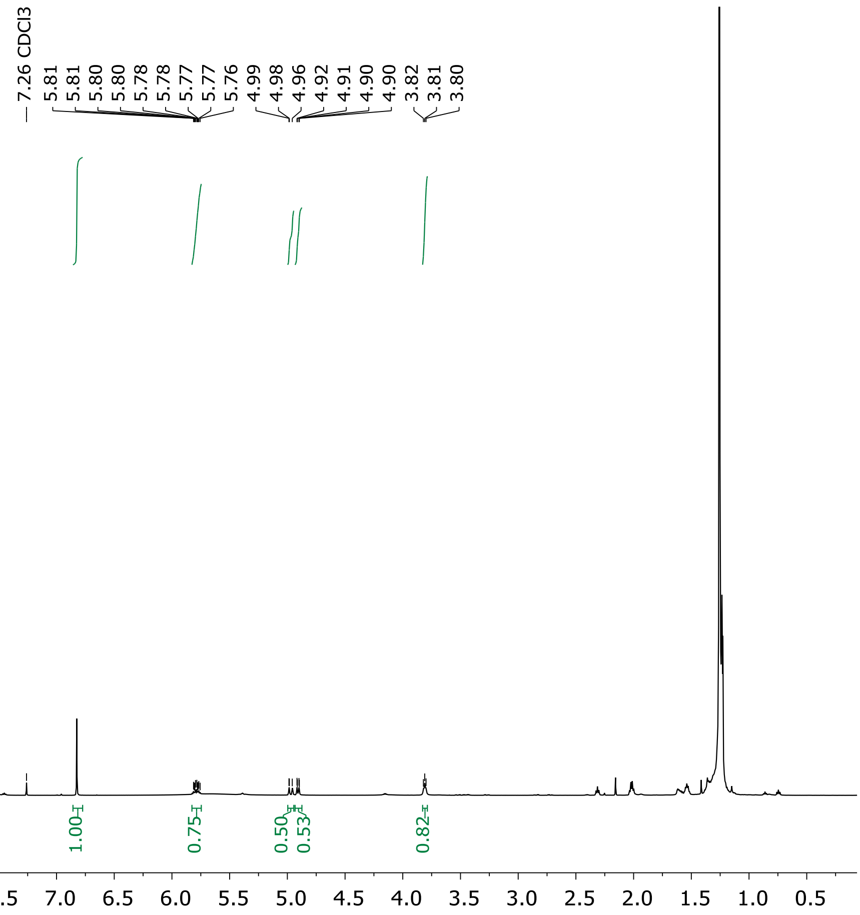

3-(4-Ethynylphenyl)-1-phenylpropan-1-ol (2p)

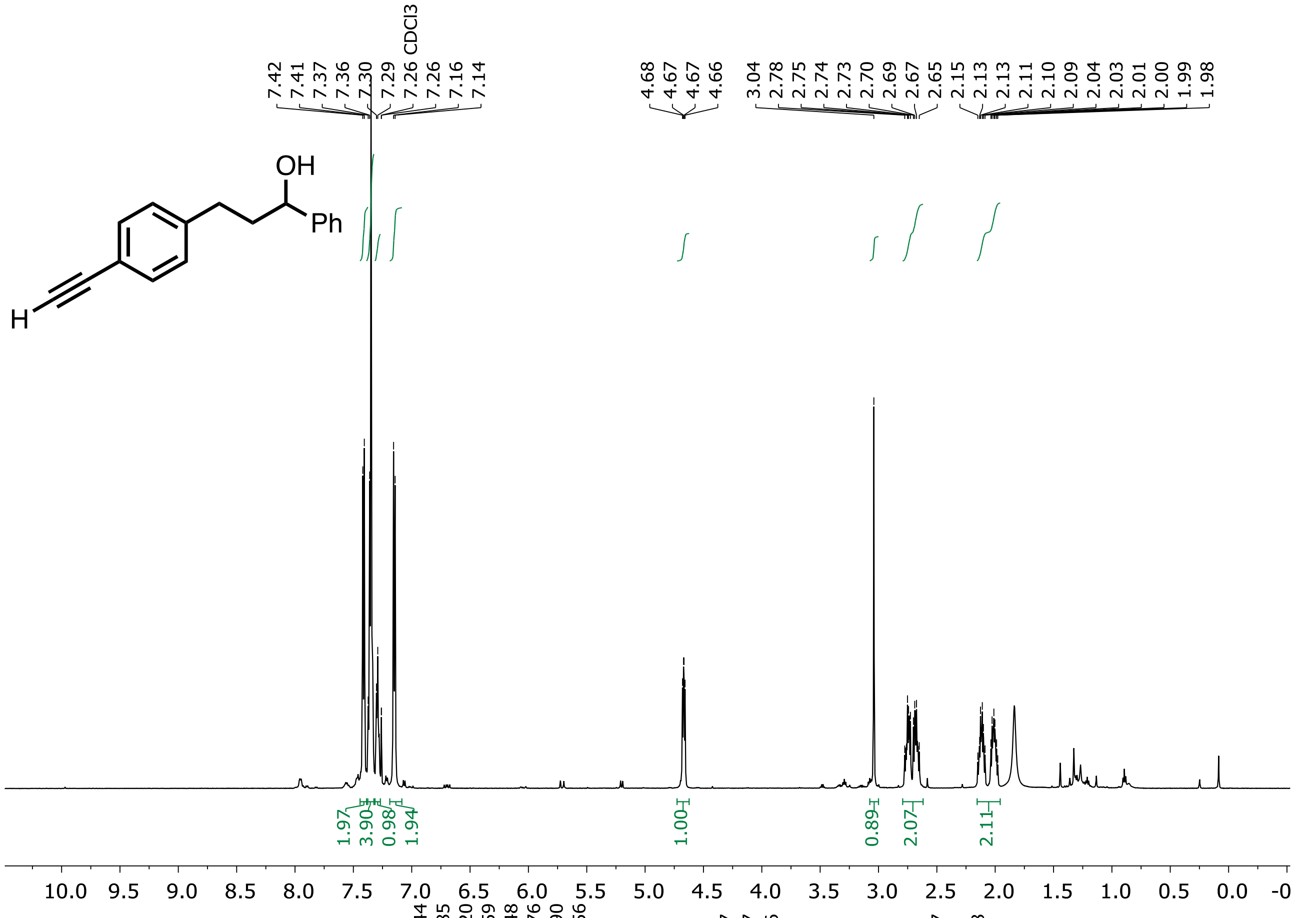

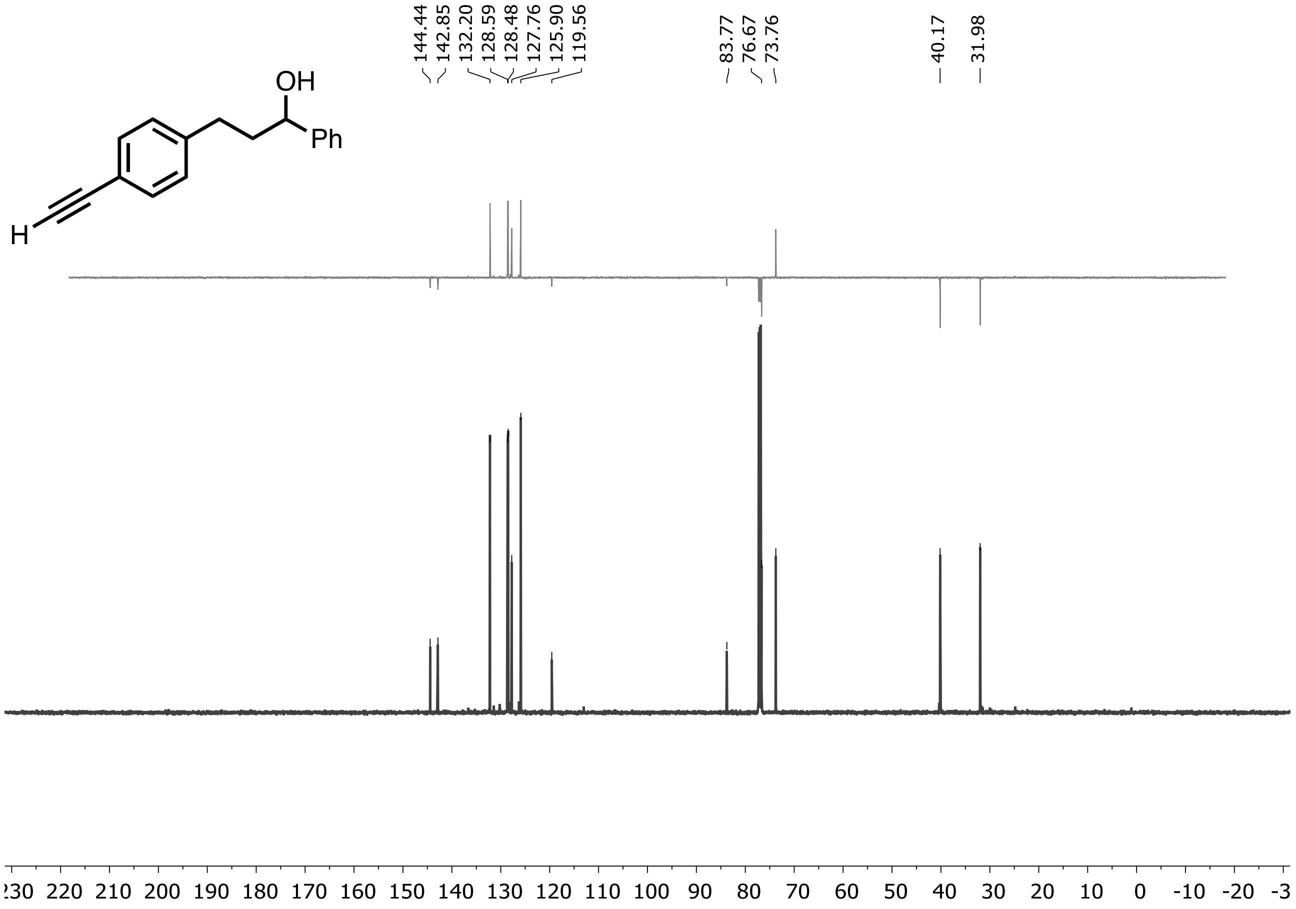

6-Phenyltetrahydro-2*H*-pyran-2-one (3a)

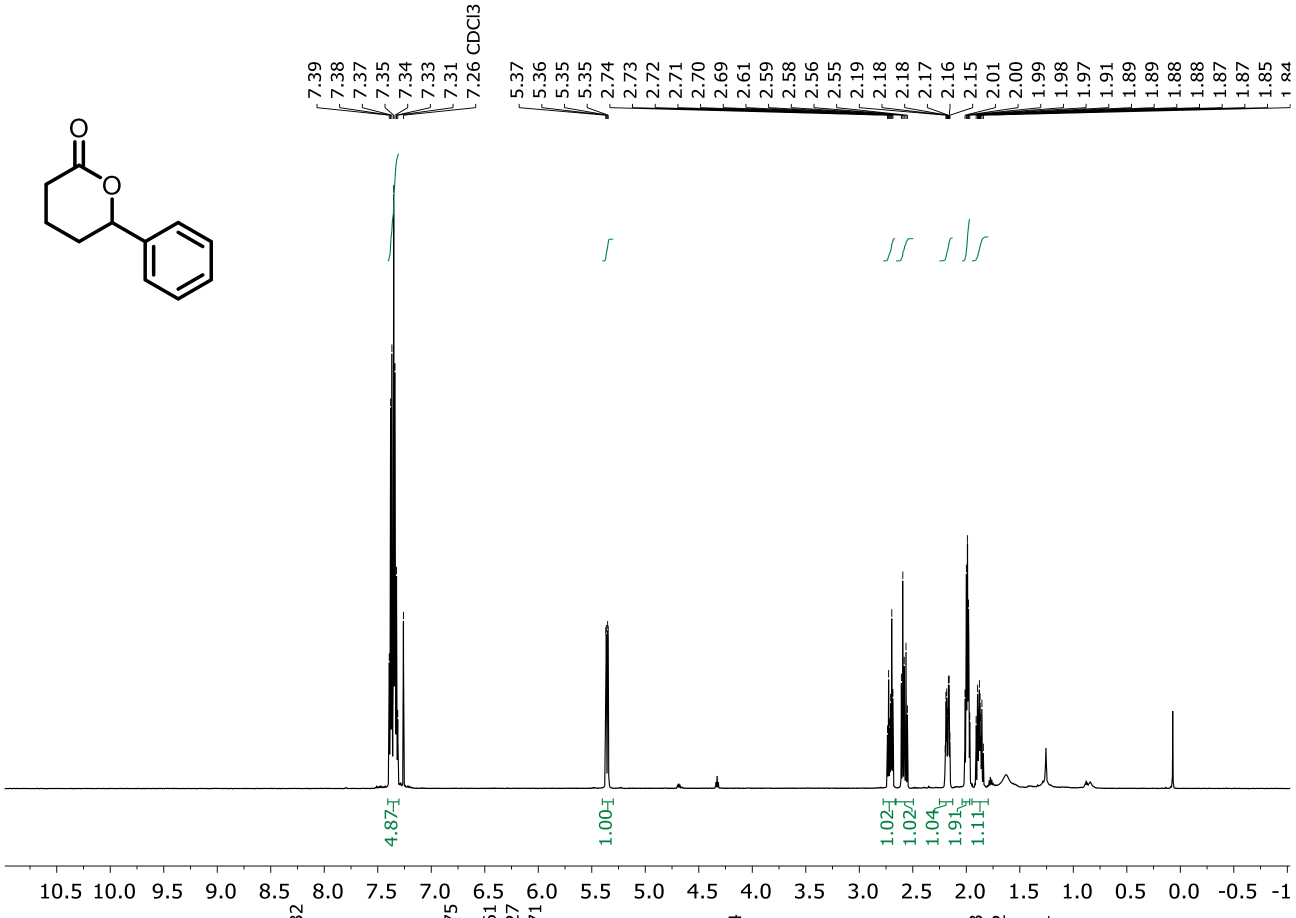

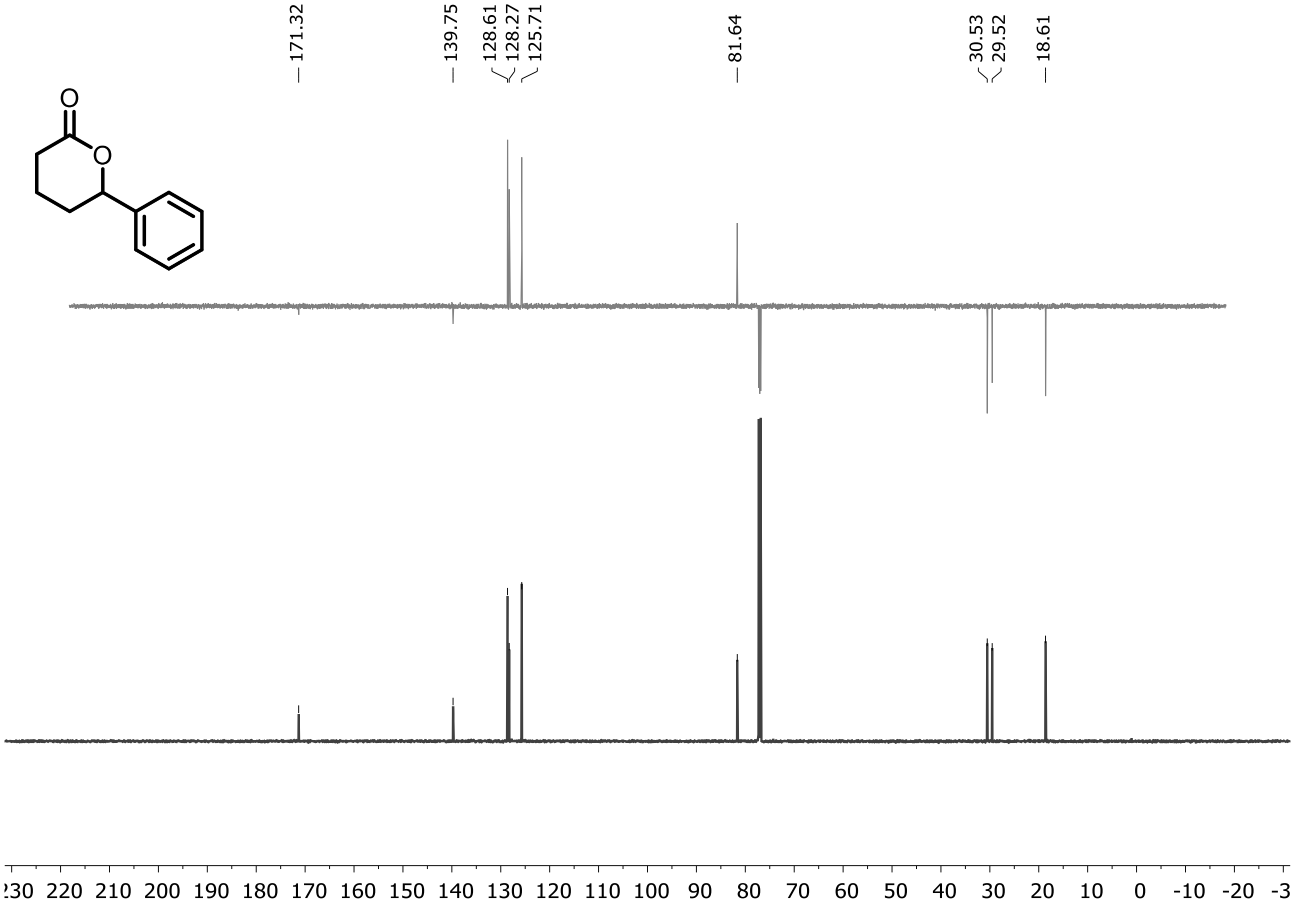

6-(*p*-Tolyl)tetrahydro-2*H*-pyran-2-one (3b)

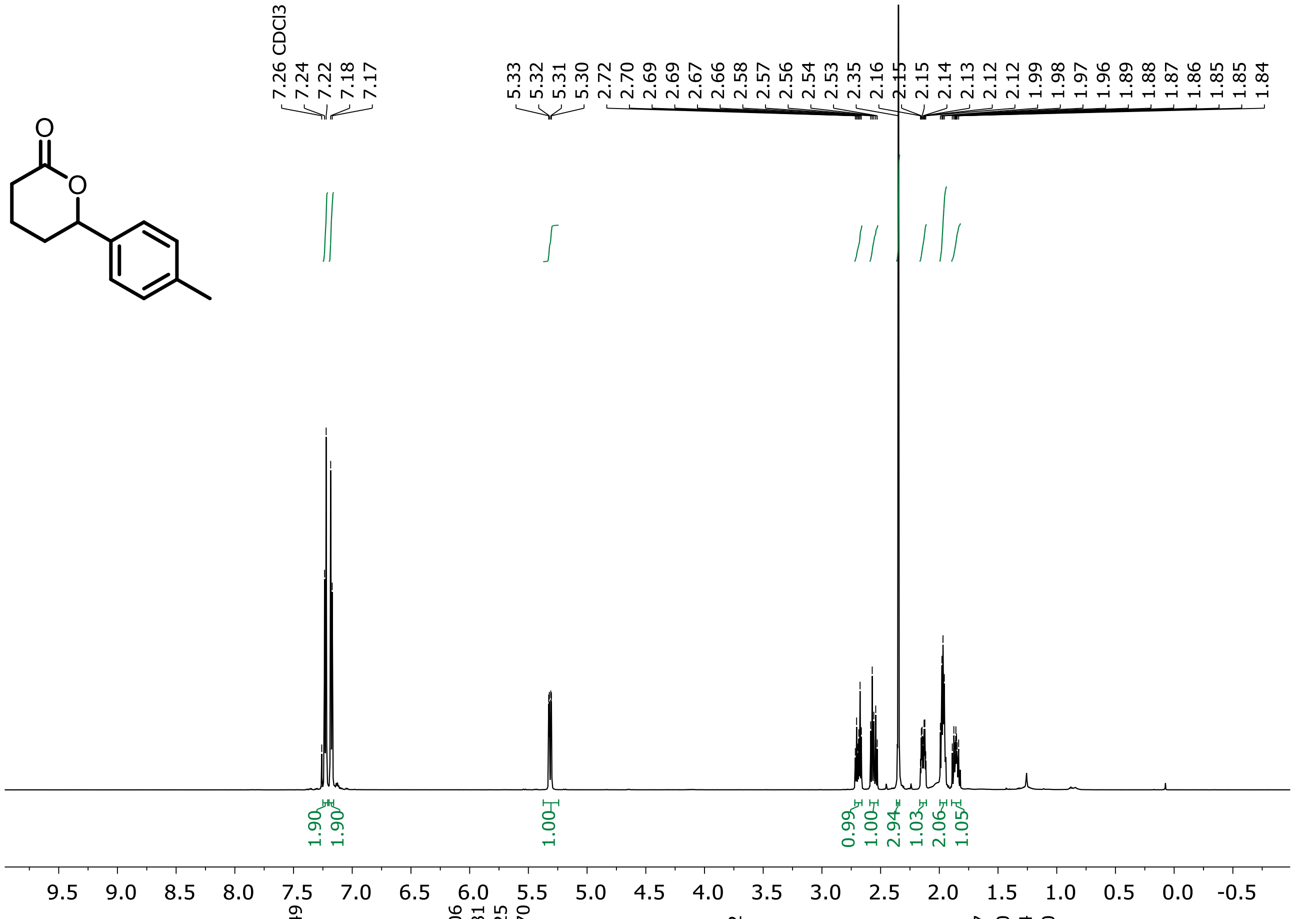

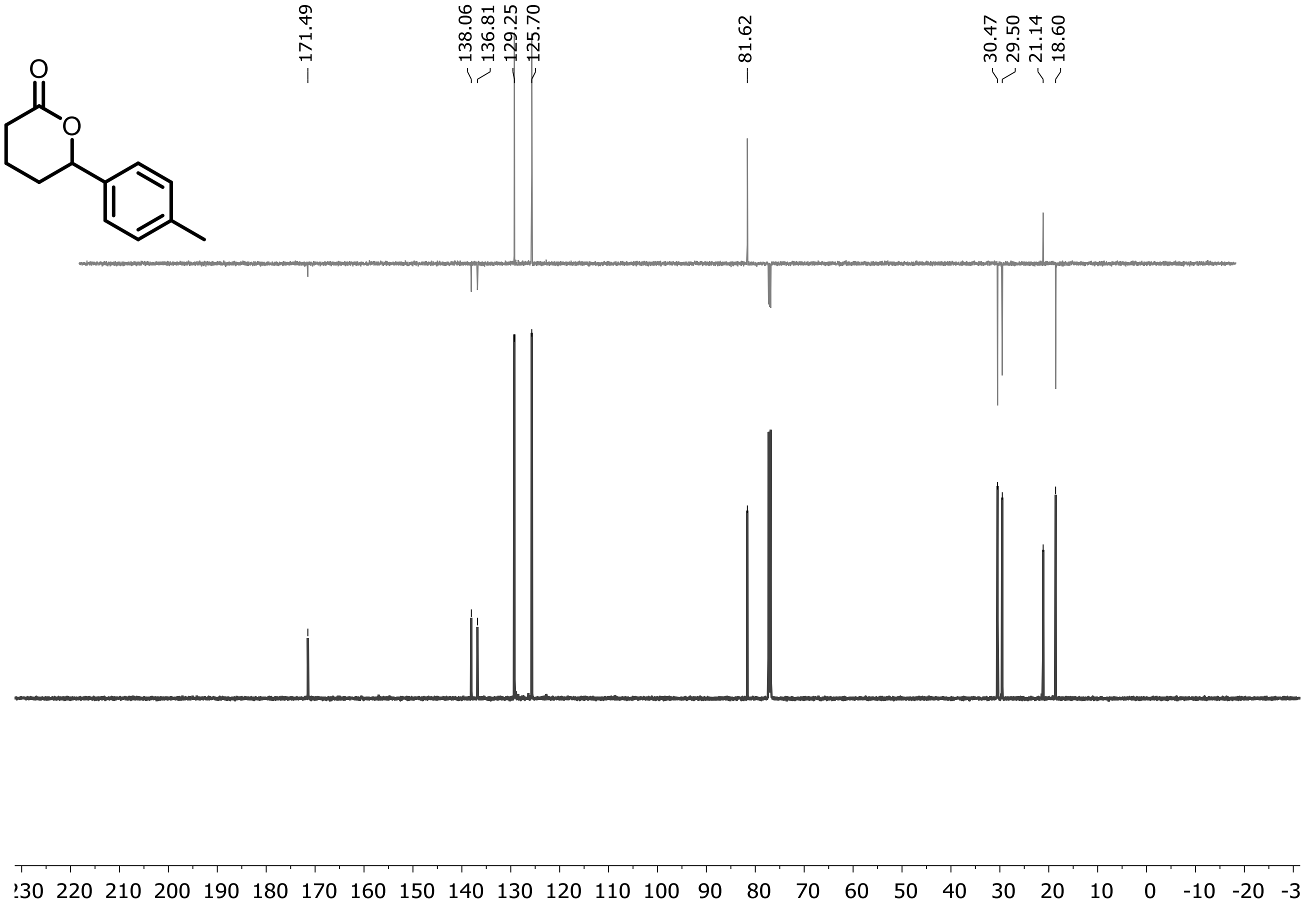

6-(4-Fluorophenyl)tetrahydro-2*H*-pyran-2-one (3c)

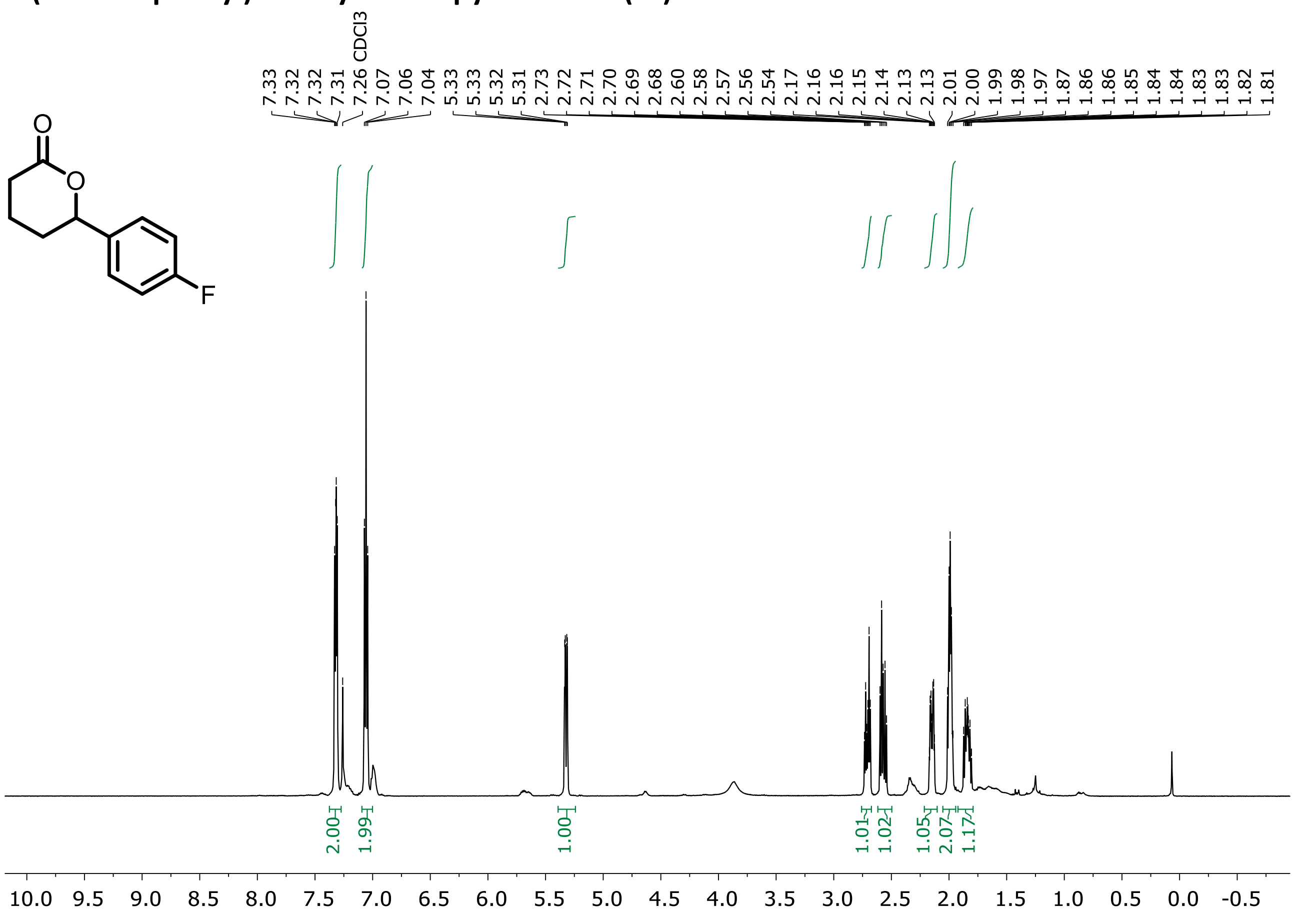

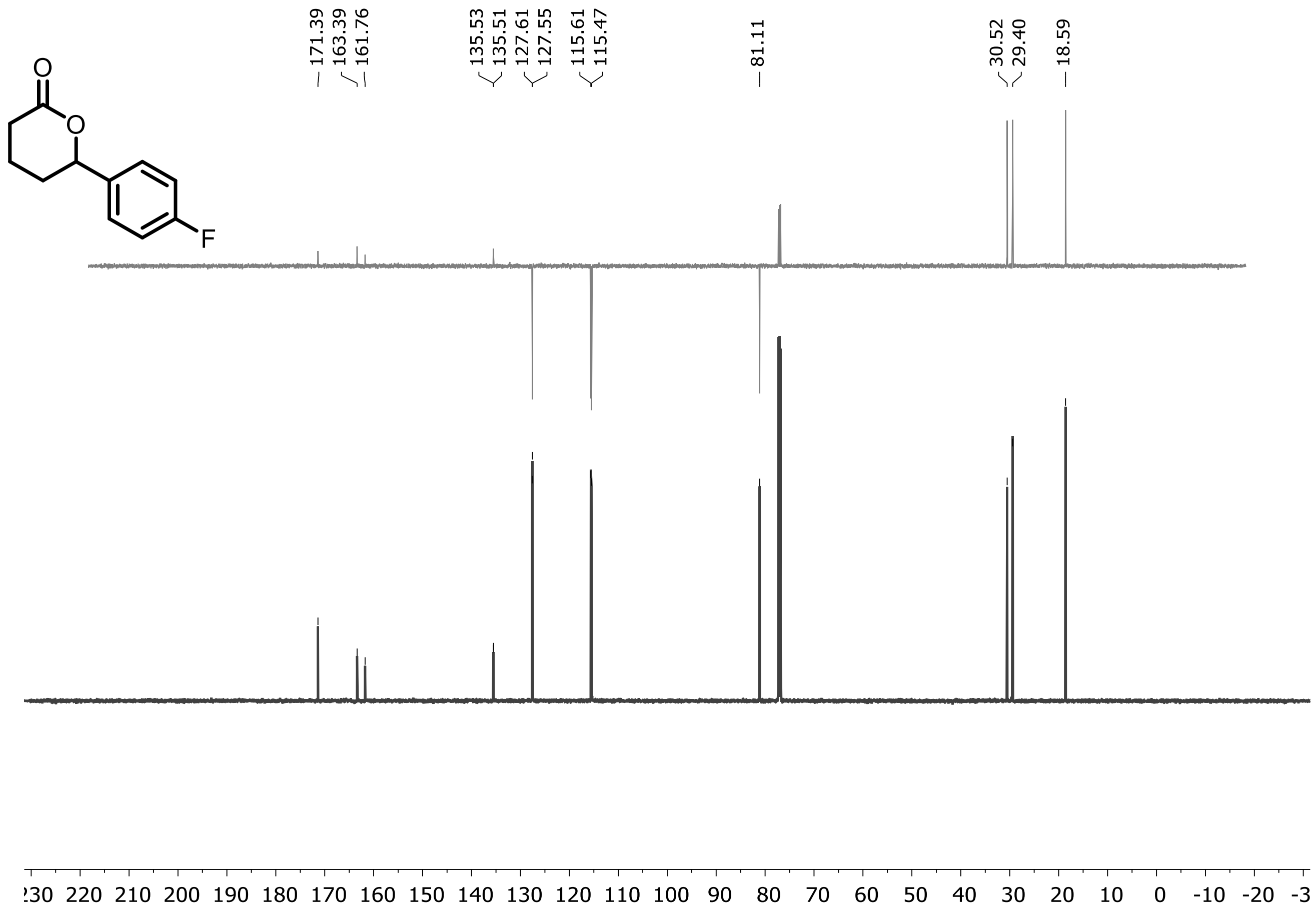

6-(4-Chlorophenyl)tetrahydro-2*H*-pyran-2-one (3d)

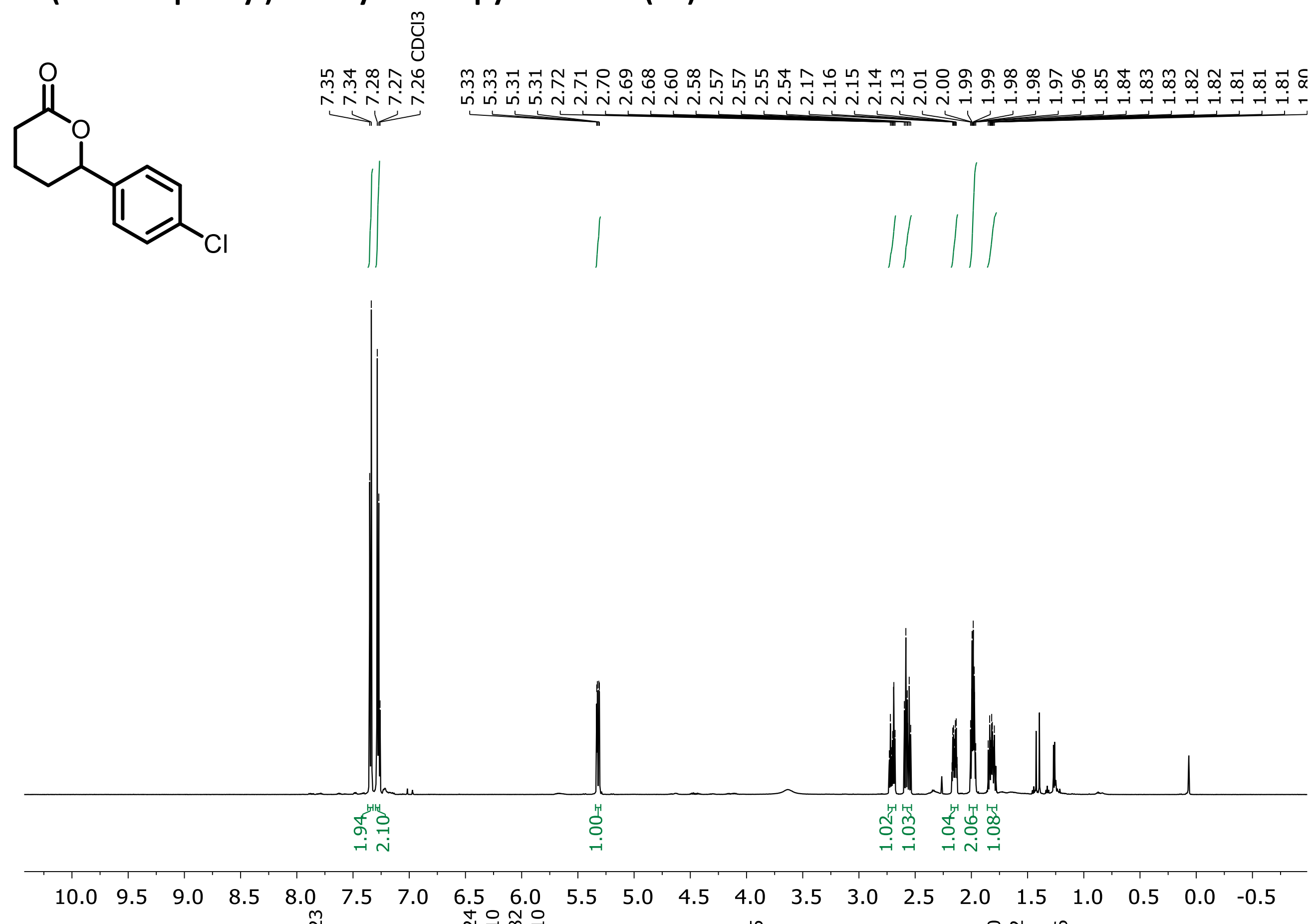

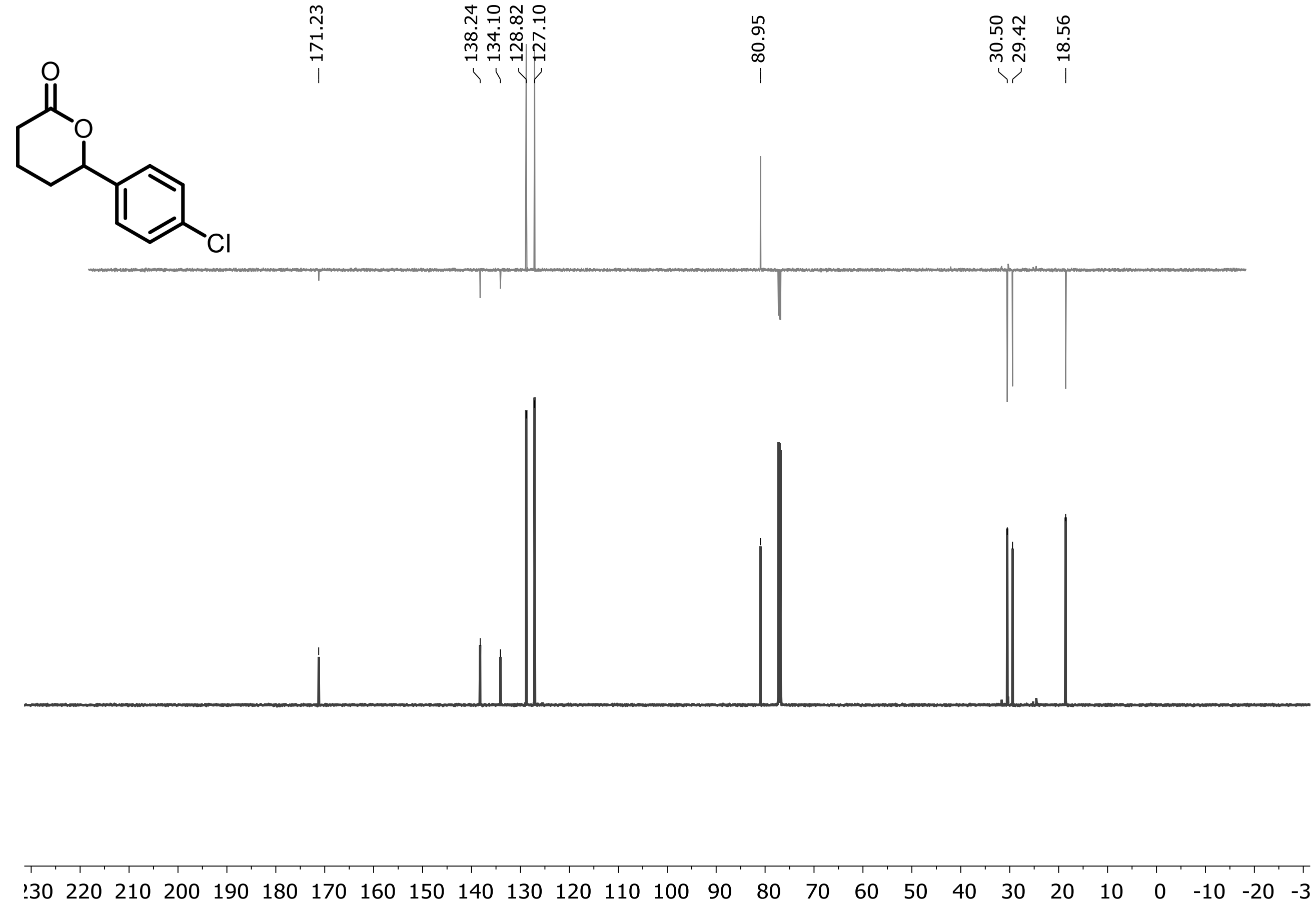

6-(4-Bromophenyl)tetrahydro-2*H*-pyran-2-one (3e)

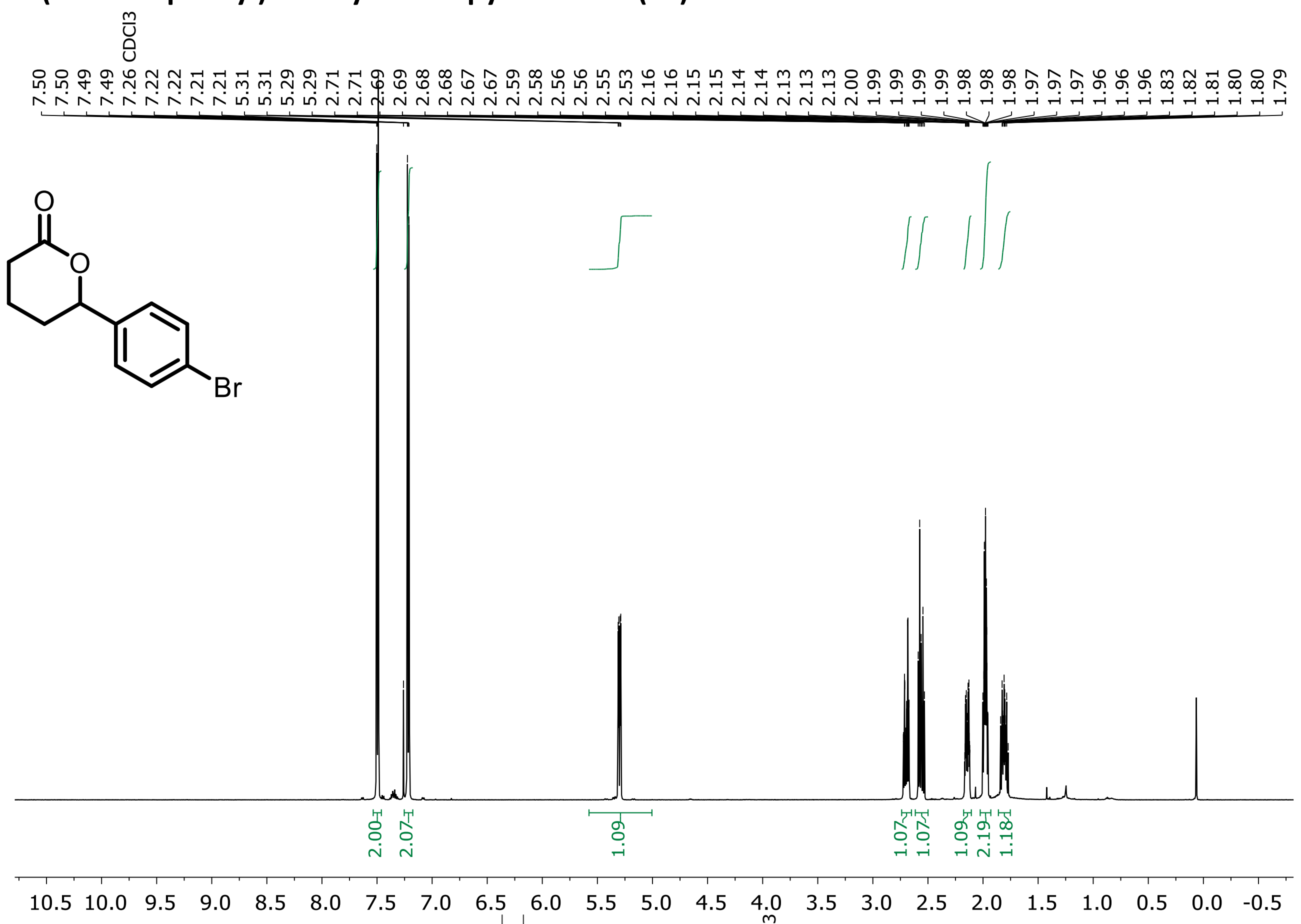

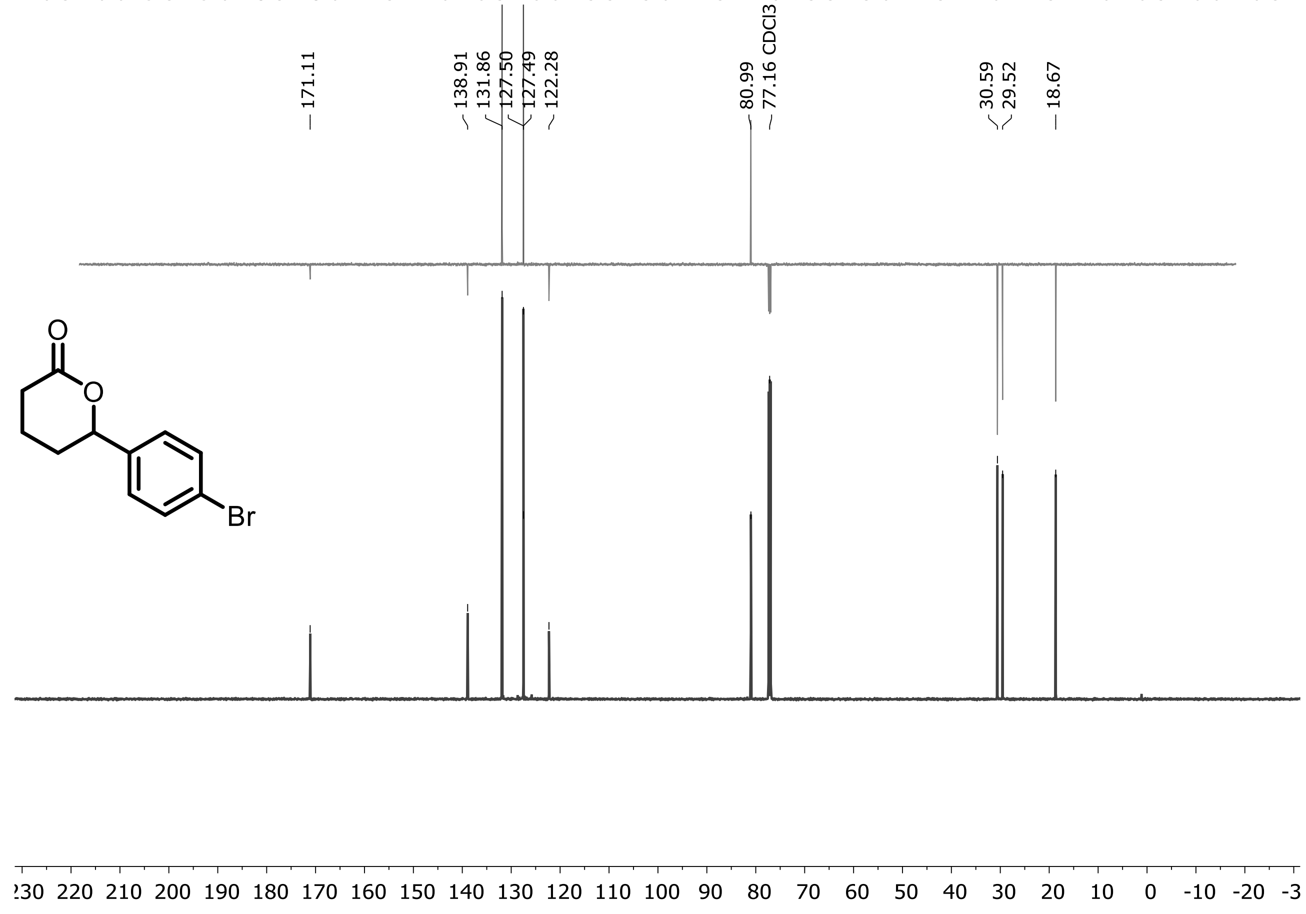

6-(4-Methoxyphenyl)tetrahydro-2*H*-pyran-2-one (3f)

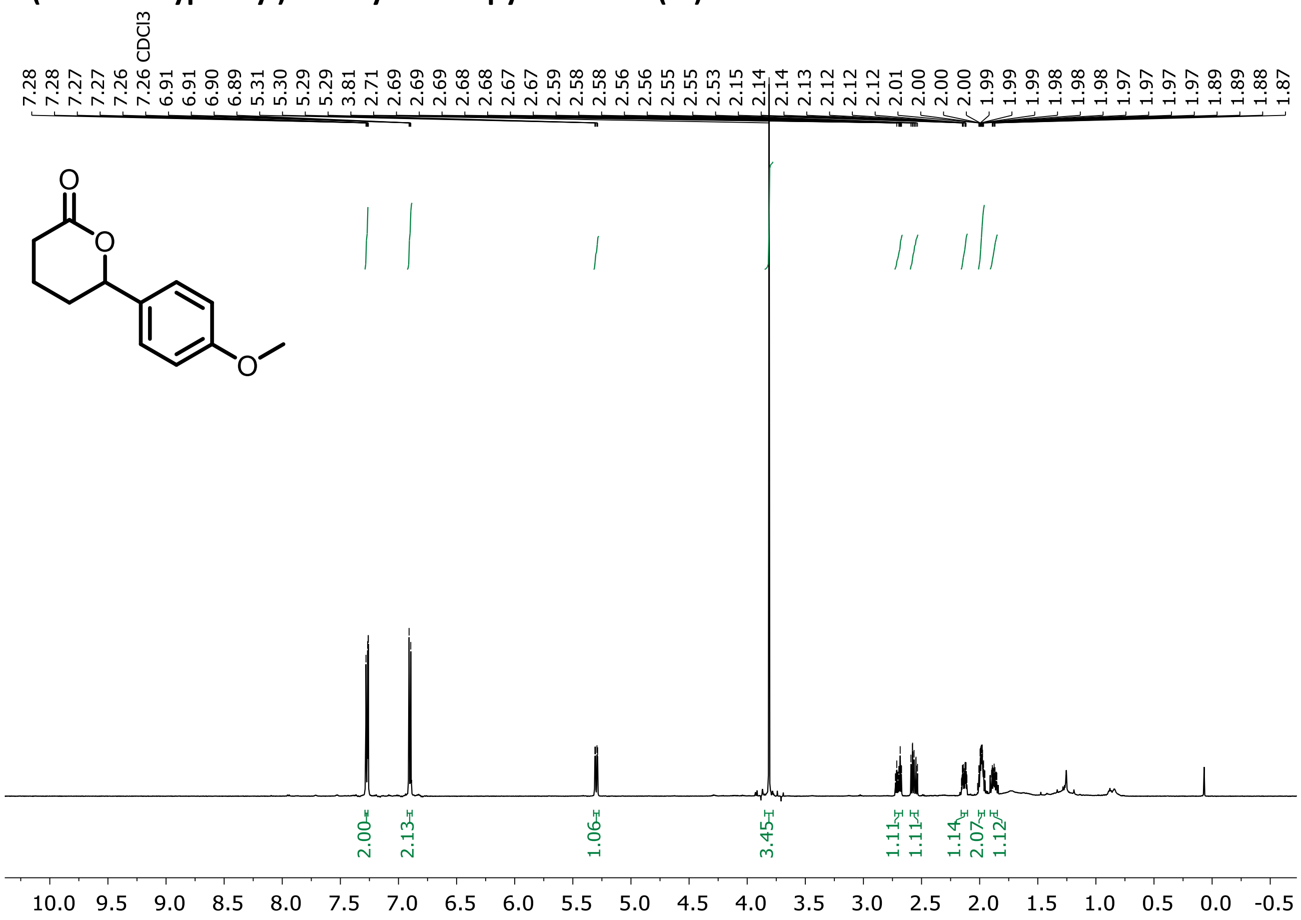

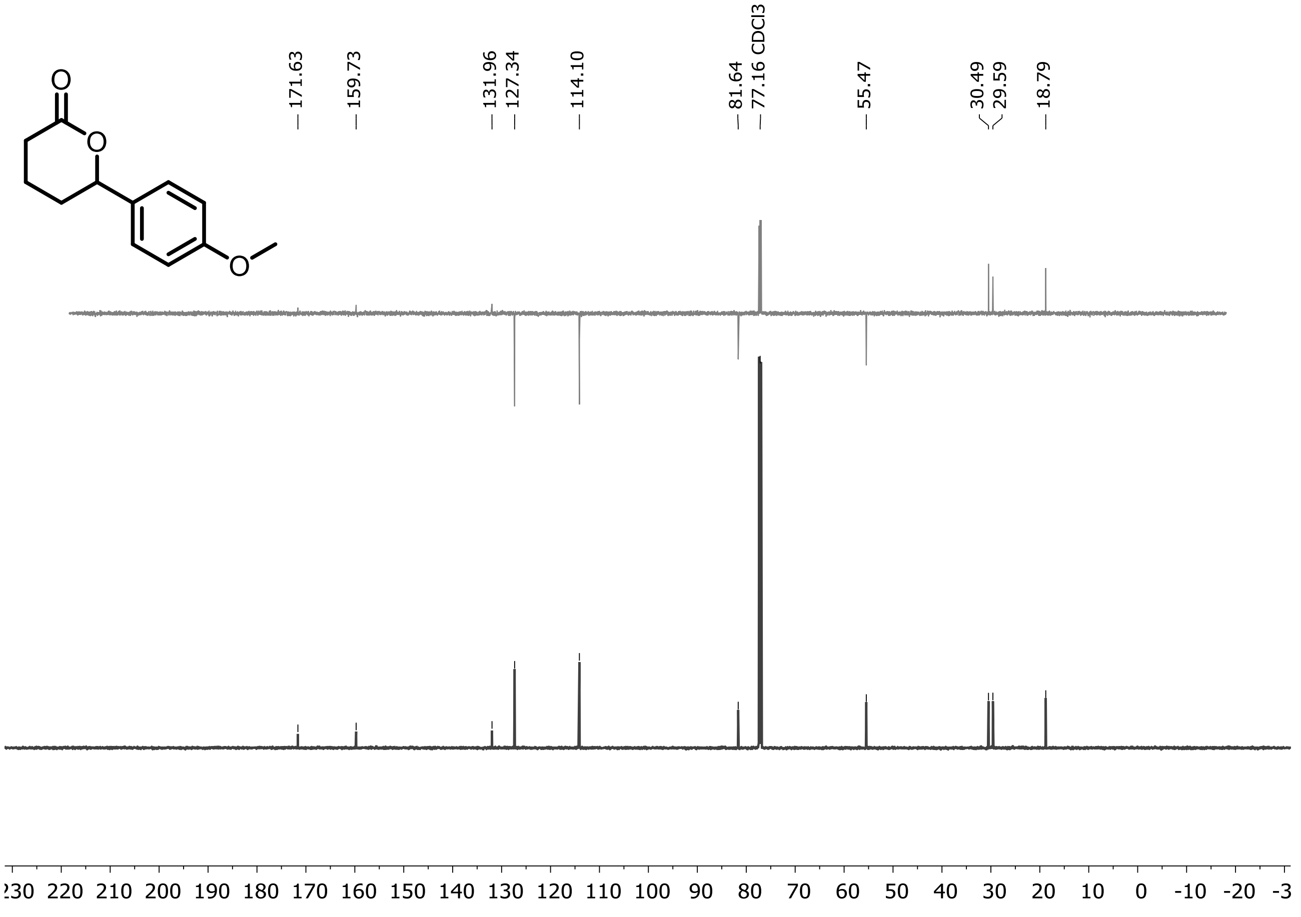

6-Hydroxy-6-phenylhexanoic acid (3g)

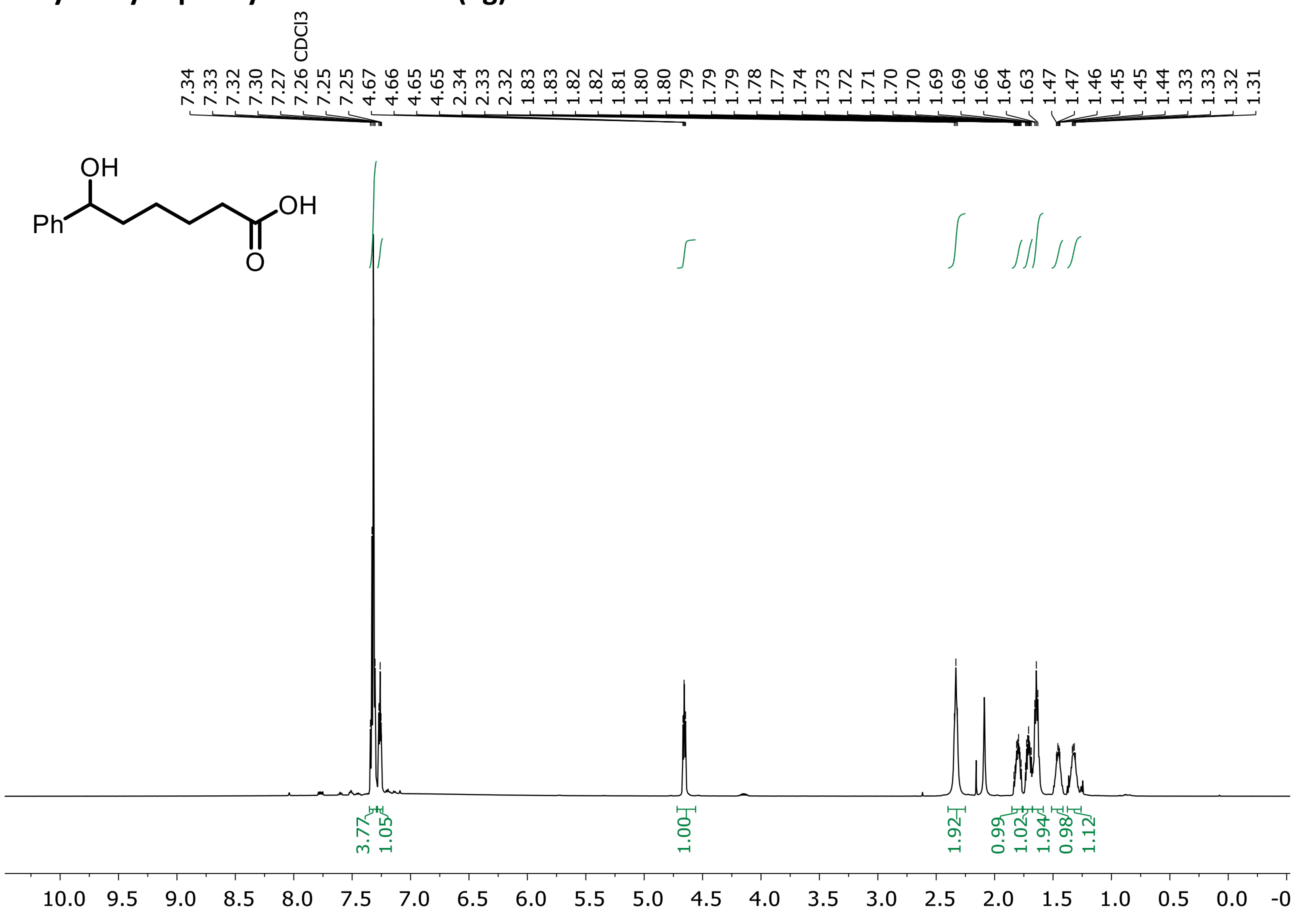

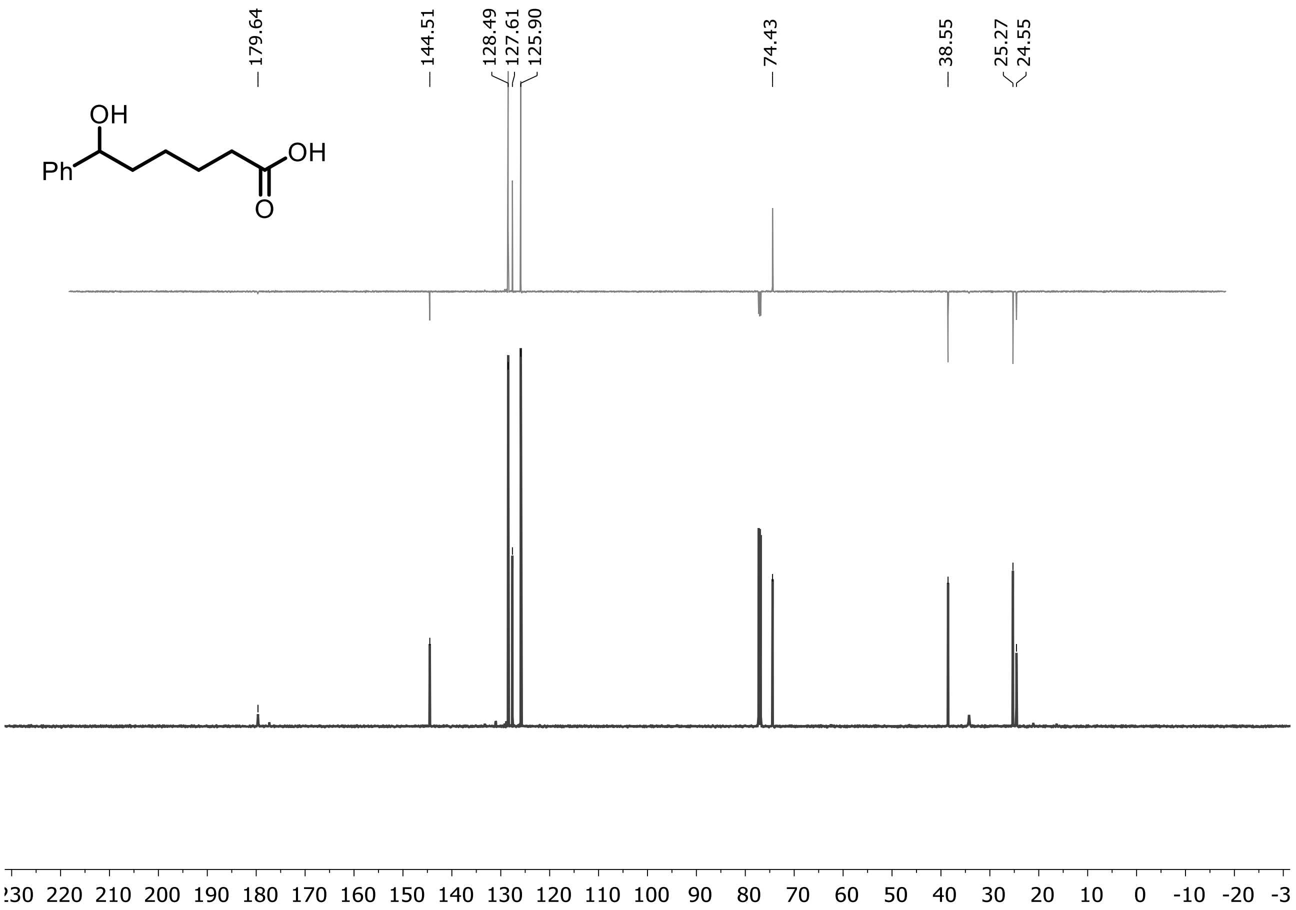

7-Hydroxy-7-phenylheptanoic acid (3h)

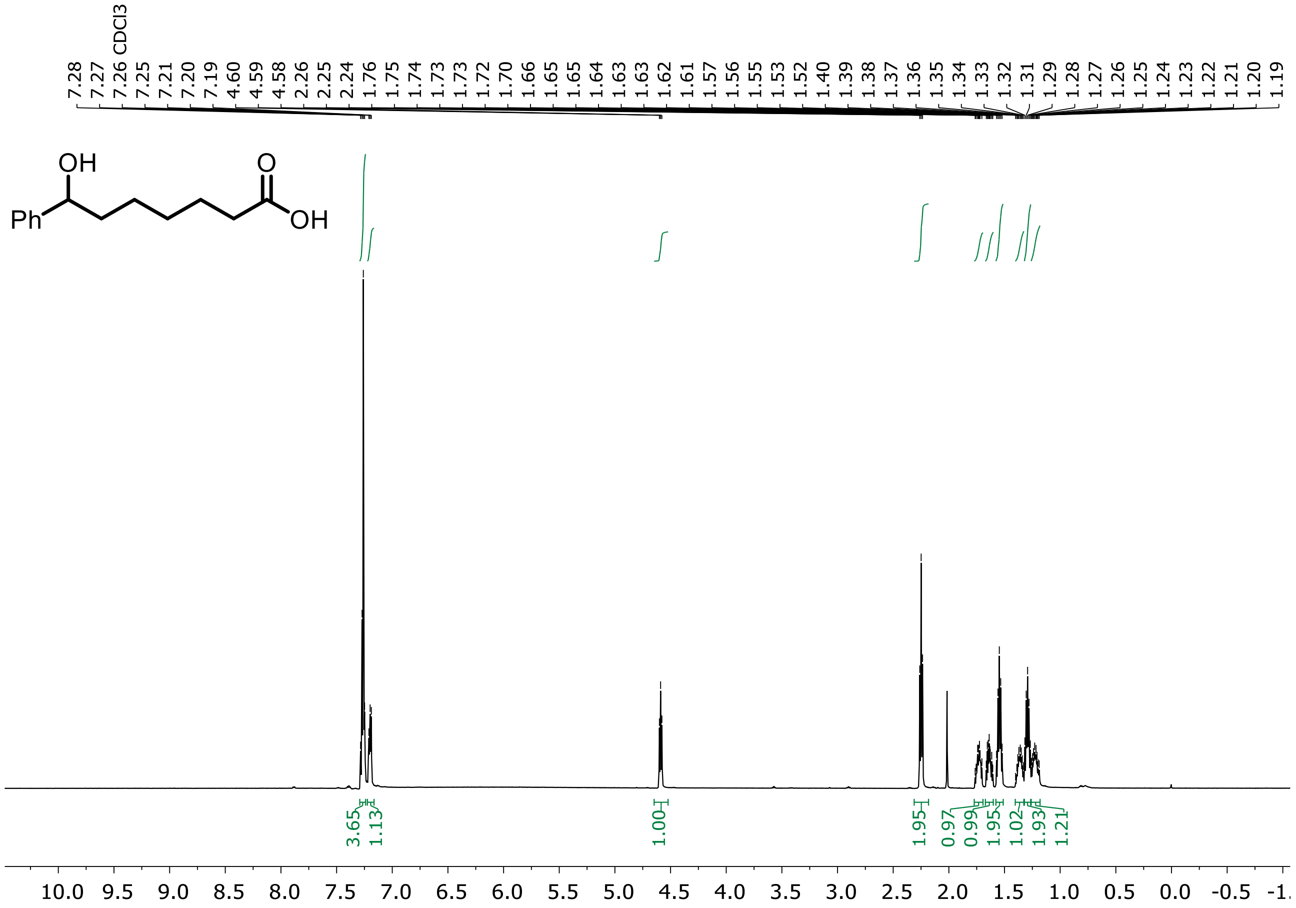

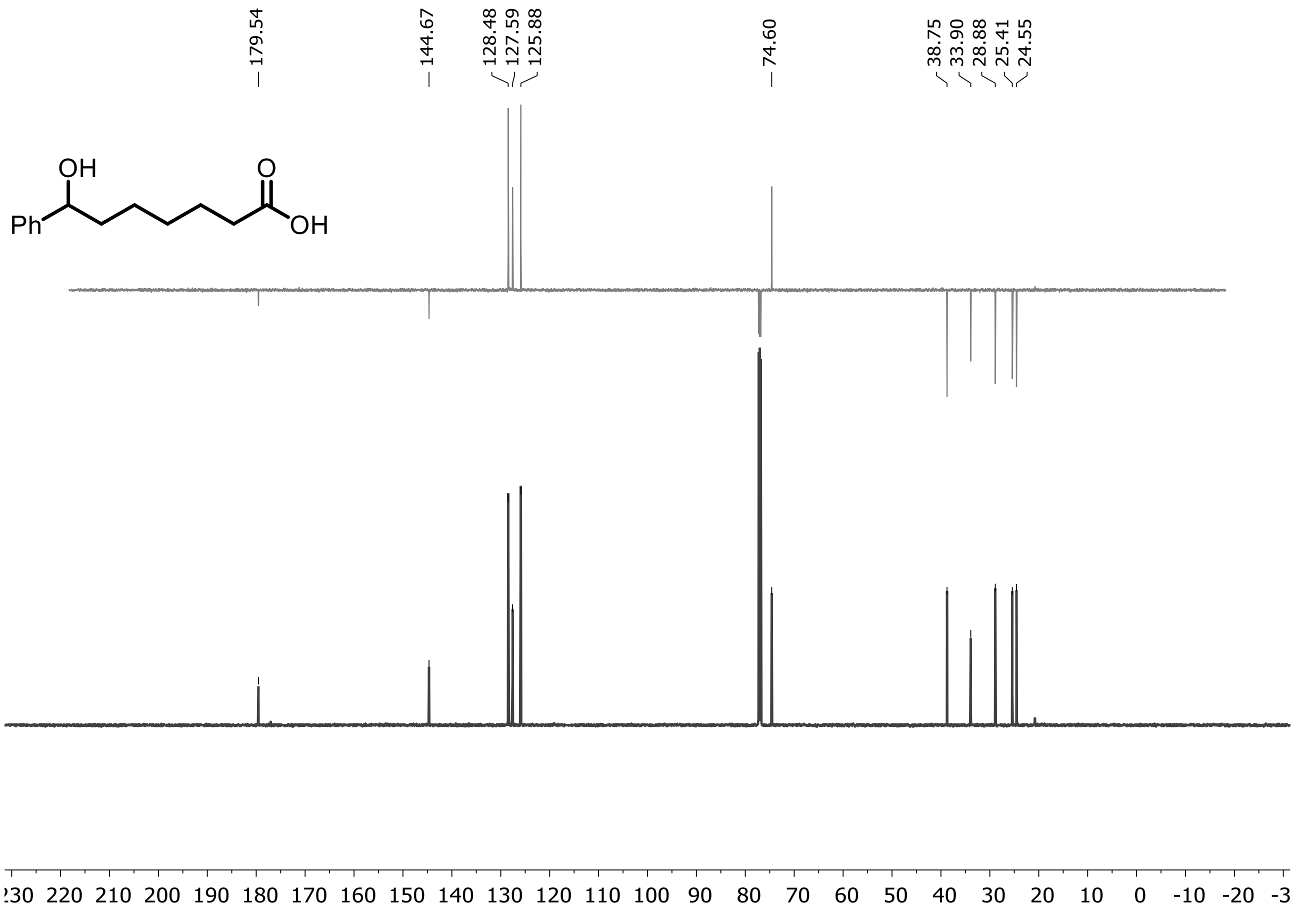

8-Hydroxy-8-phenyloctanoic acid (3i)

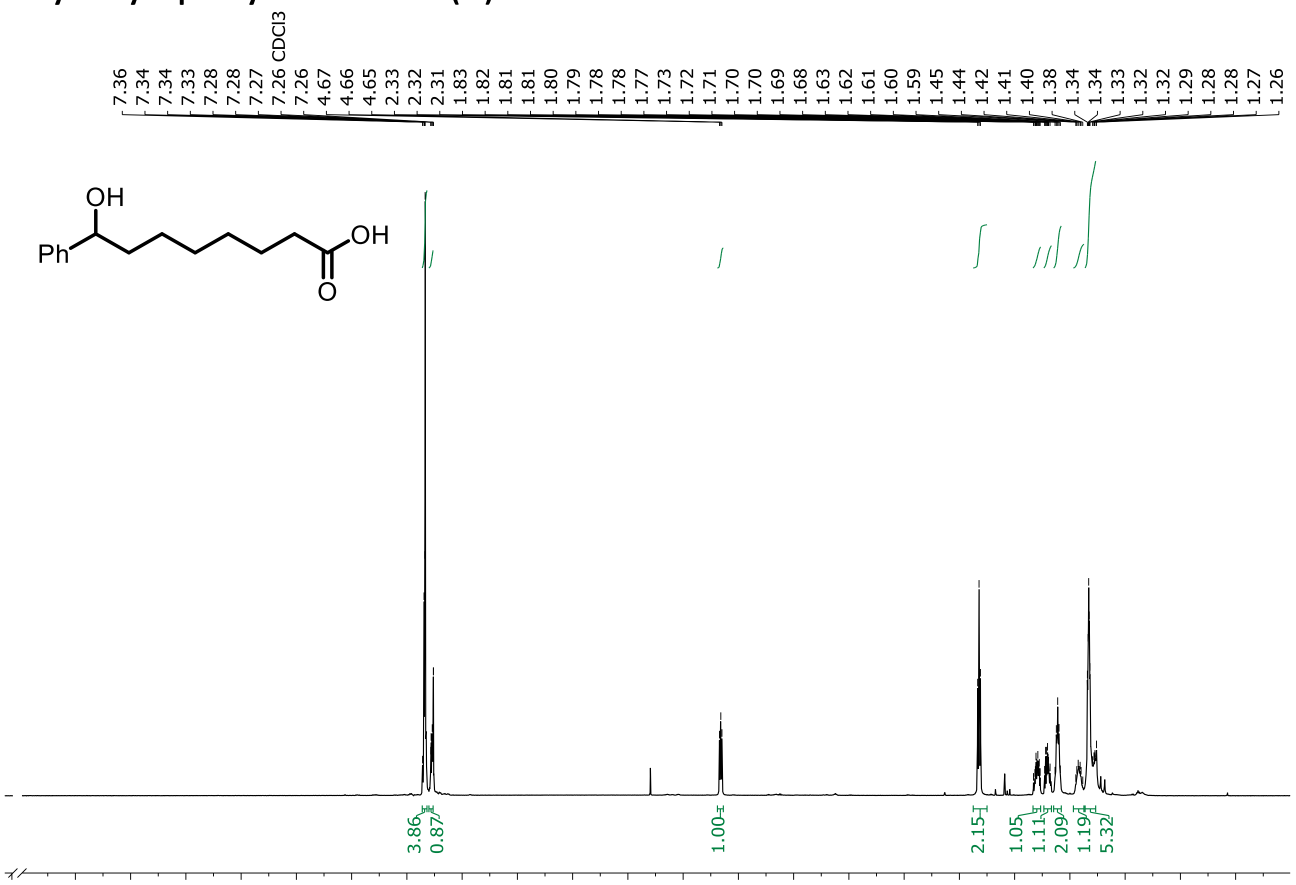

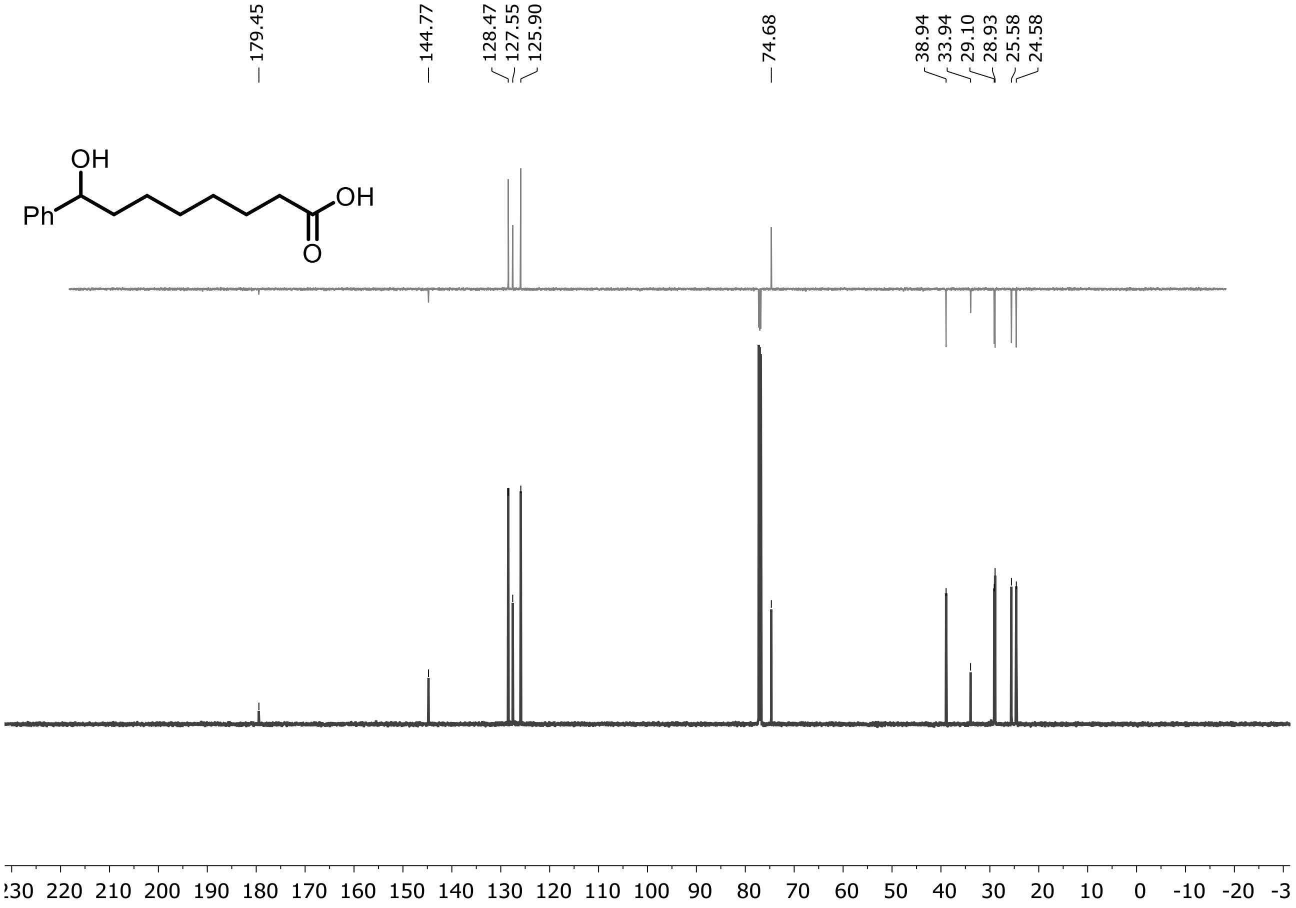

10-Hydroxytetradecanoic acid (3j)

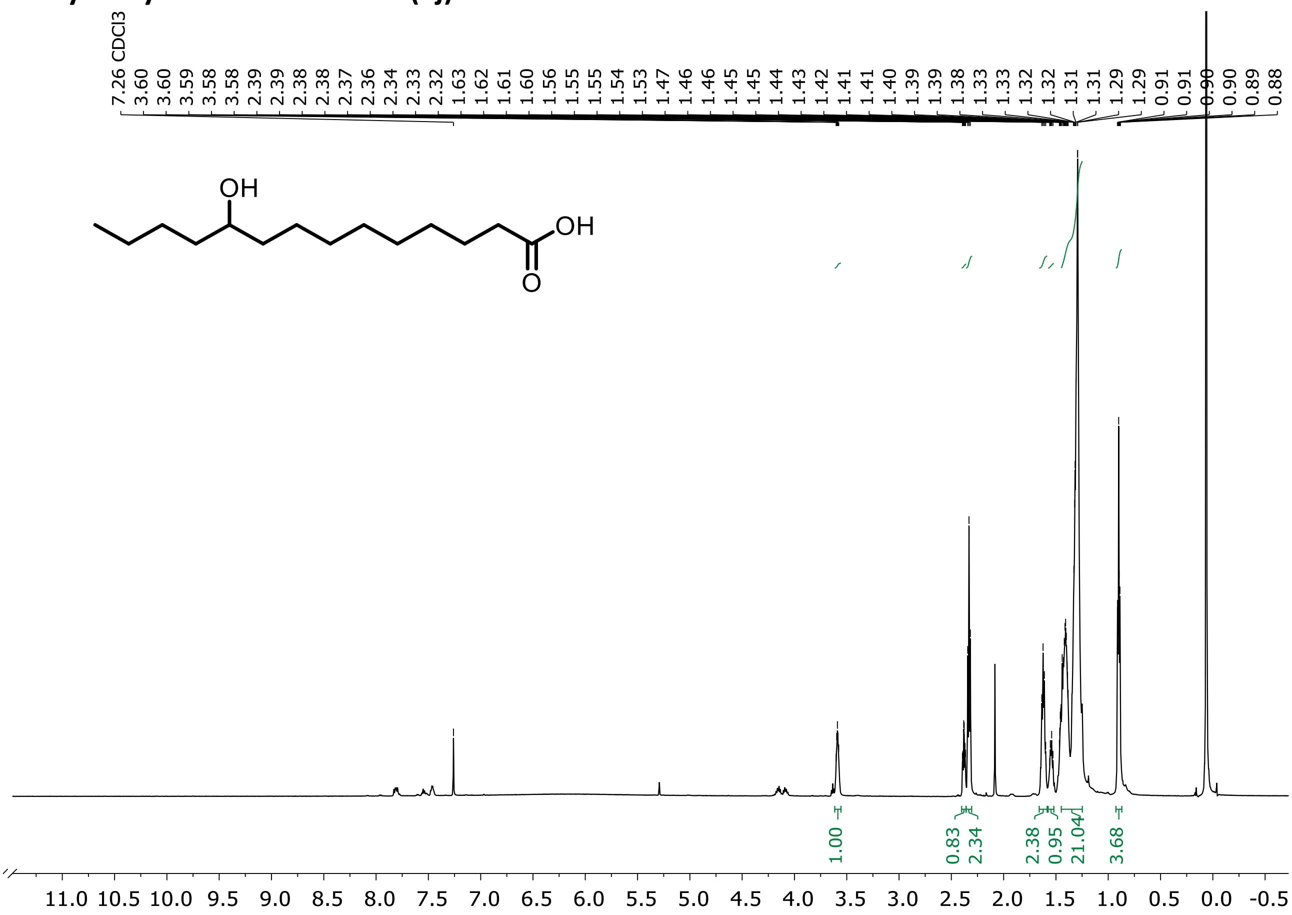

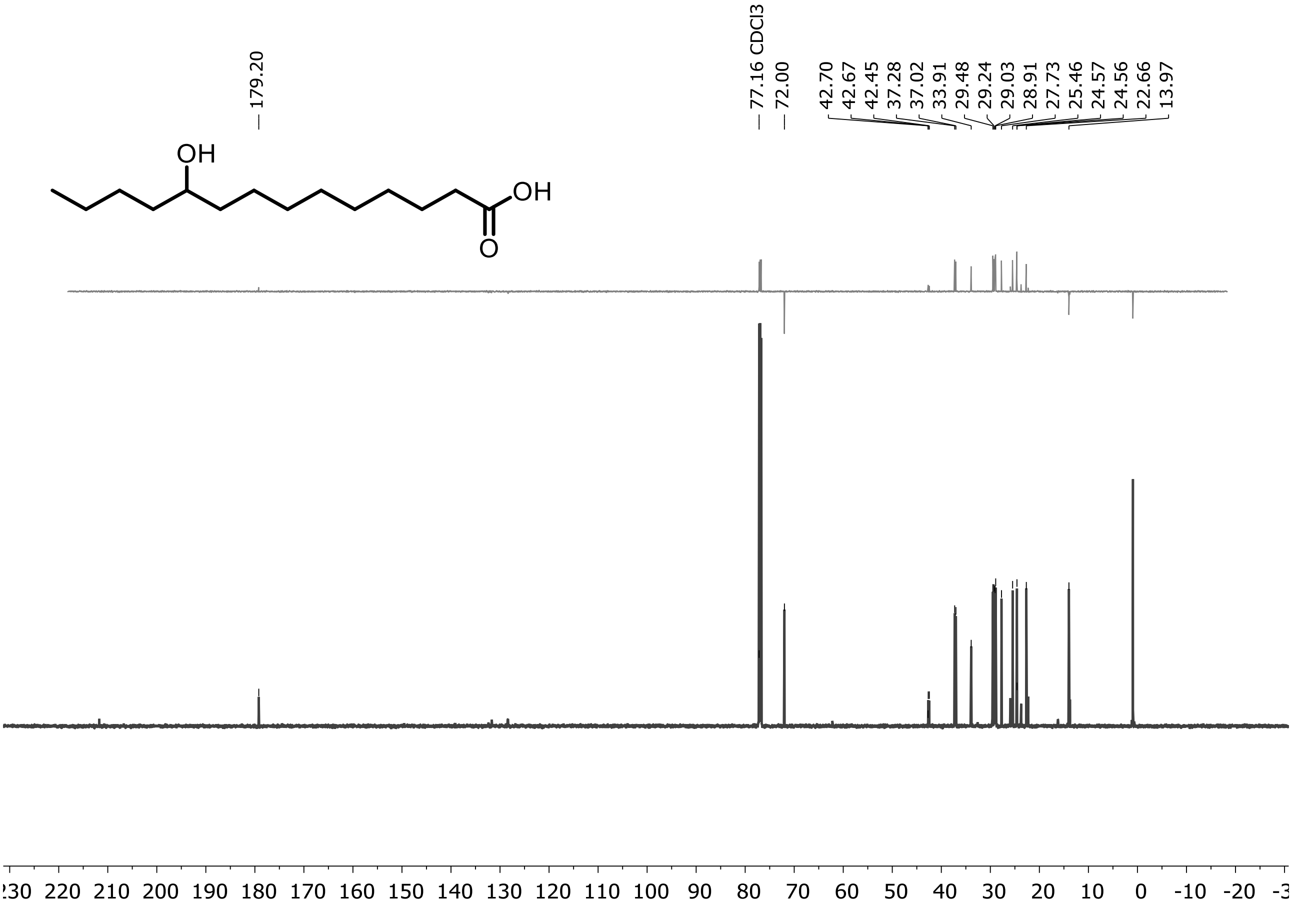

2-(11-Hydroxy-6,11-dihydrodibenzo[b,e]oxepin-2-yl)acetic acid (3k)

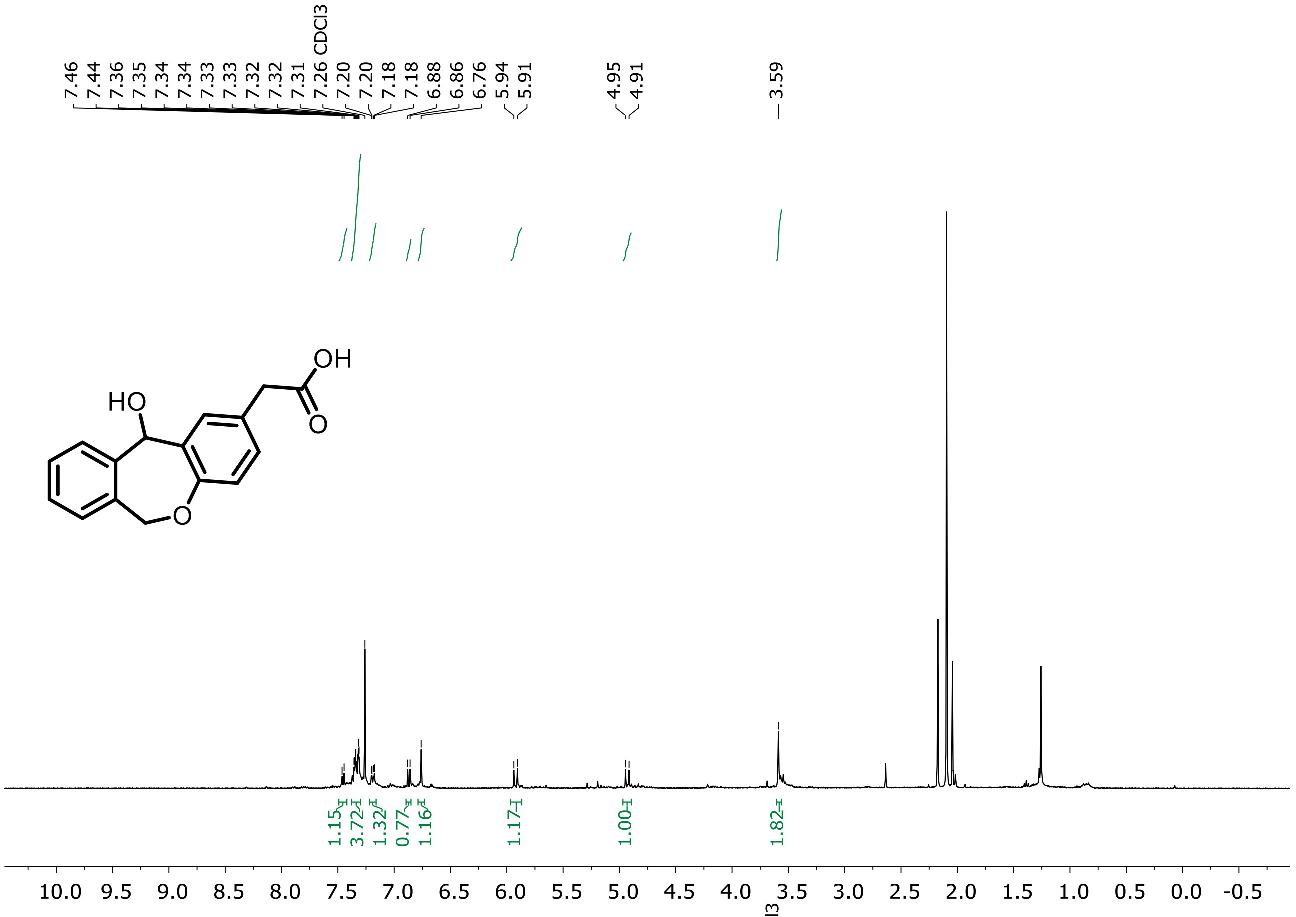

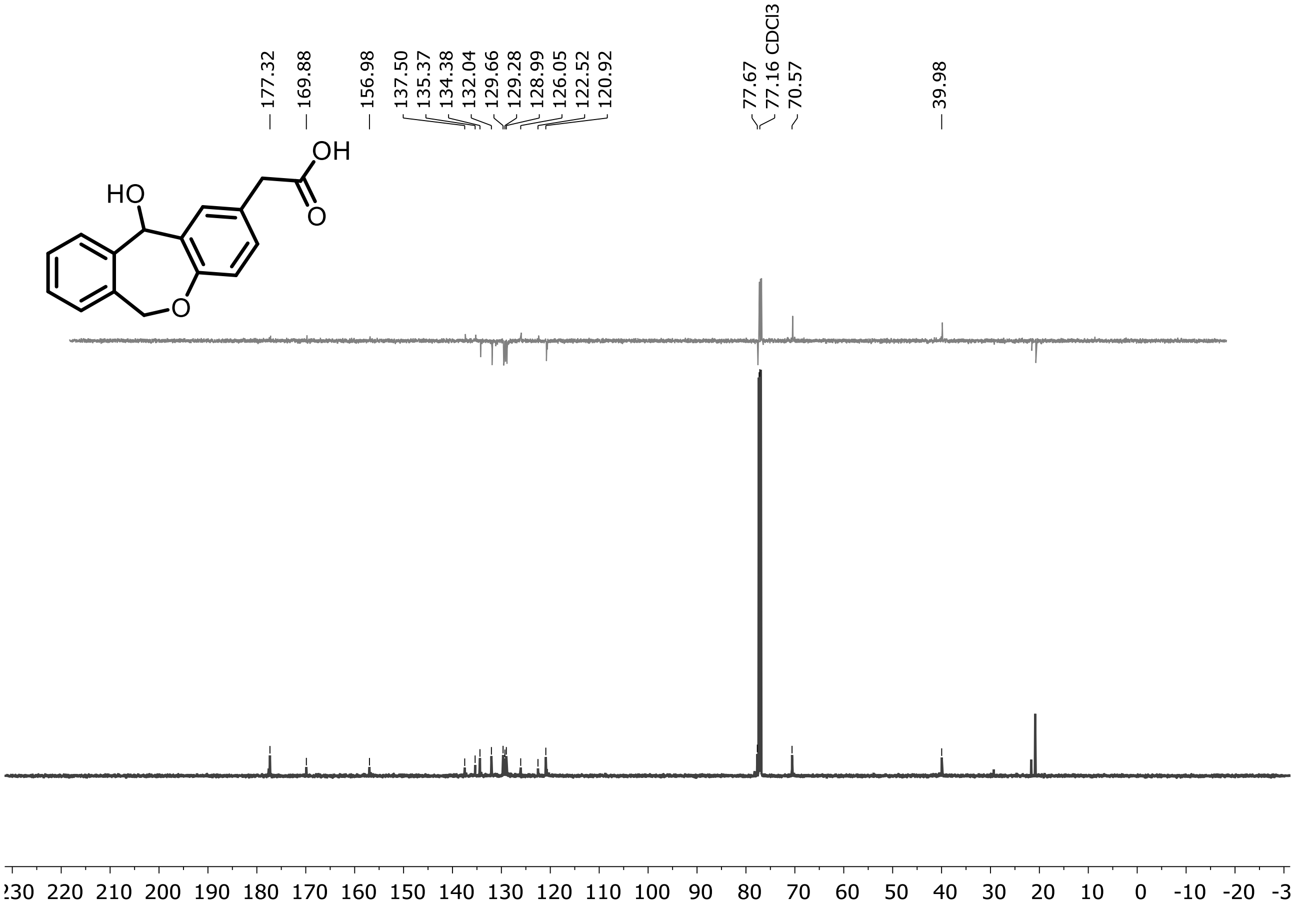

2-(3-(Hydroxy(phenyl)methyl)phenyl)propanoic acid (3l)

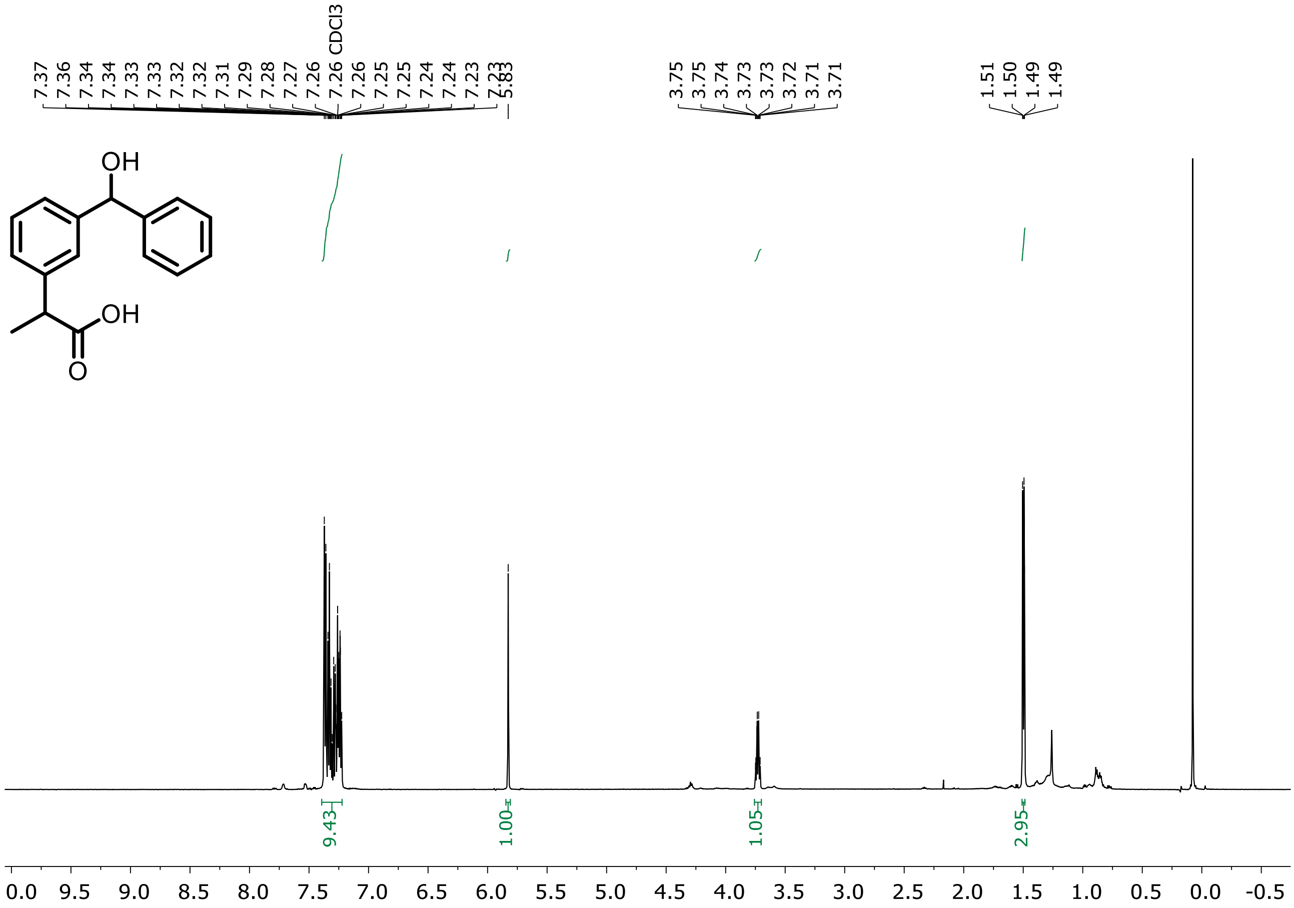

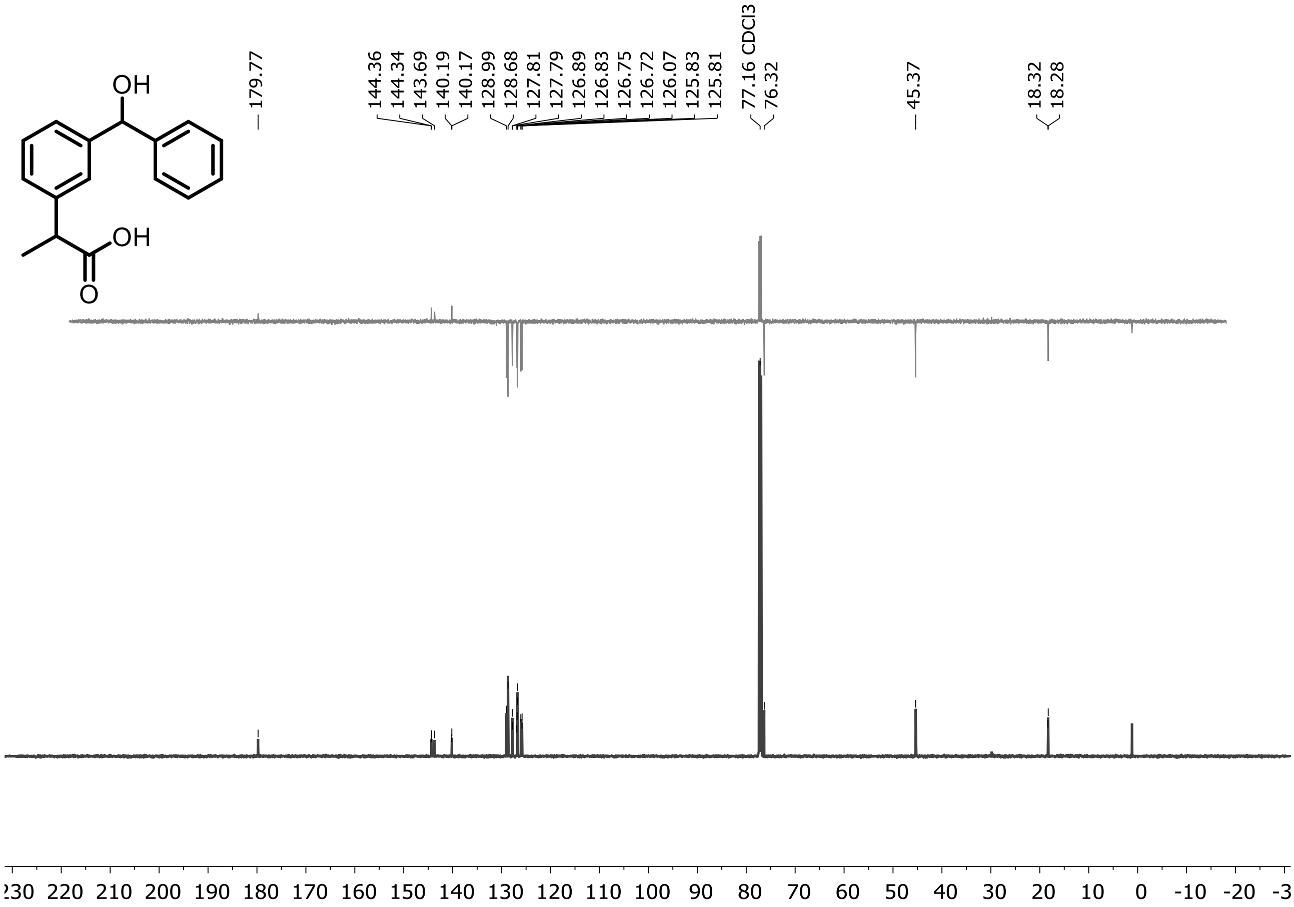

2-(10-Hydroxy-10,11-dihydrodibenzo[b,f]thiepin-2-yl)propanoic acid (3m)

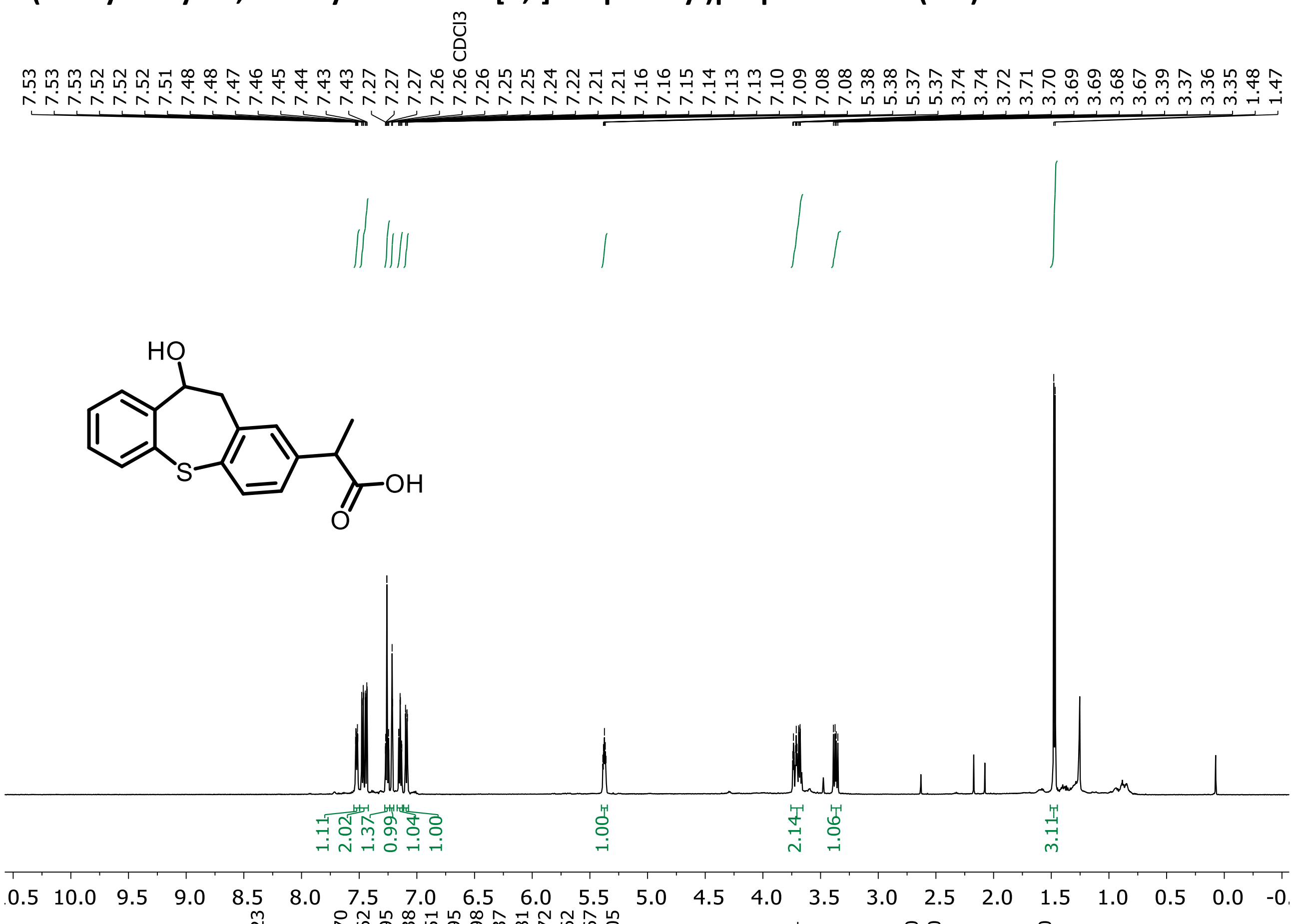

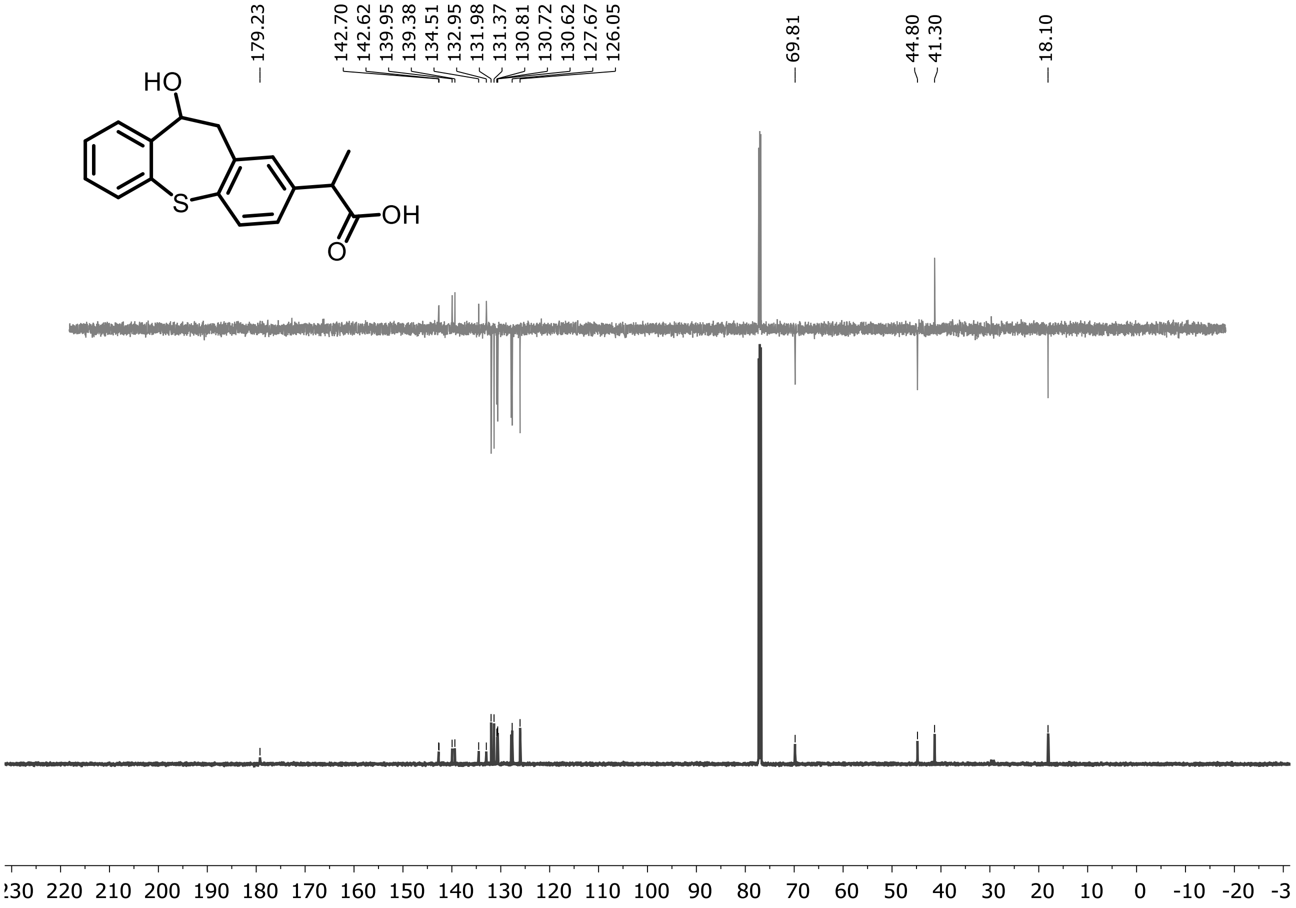

Valeric acid (3n) and 5-(4,4,5,5-tetramethyl-1,3,2-dioxaborolan-2-yl)pentanoic acid (3n')

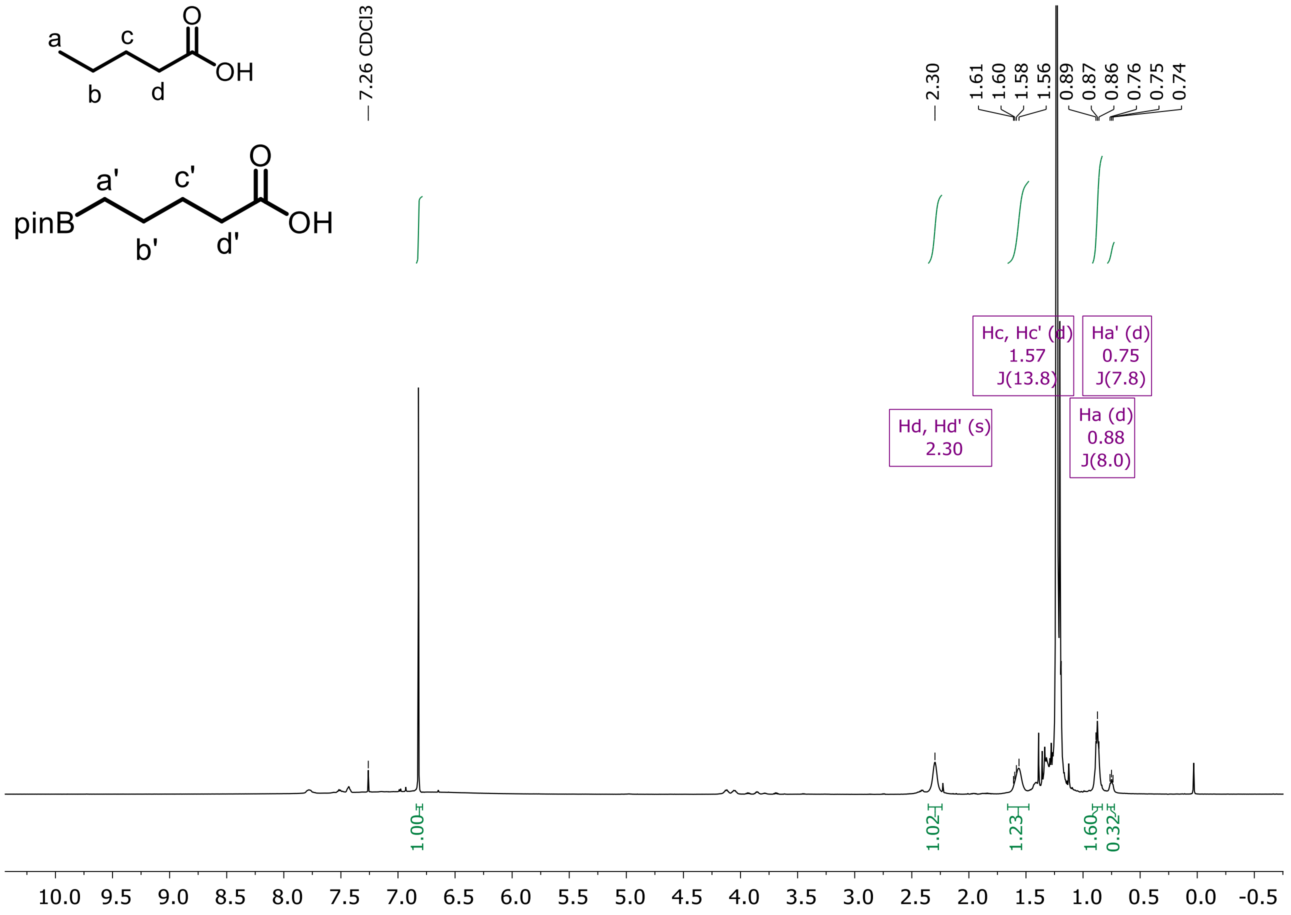

Undecanoic acid (3o) and 5-(4,4,5,5-tetramethyl-1,3,2-dioxaborolan-2-yl)undecanoic acid (3o')

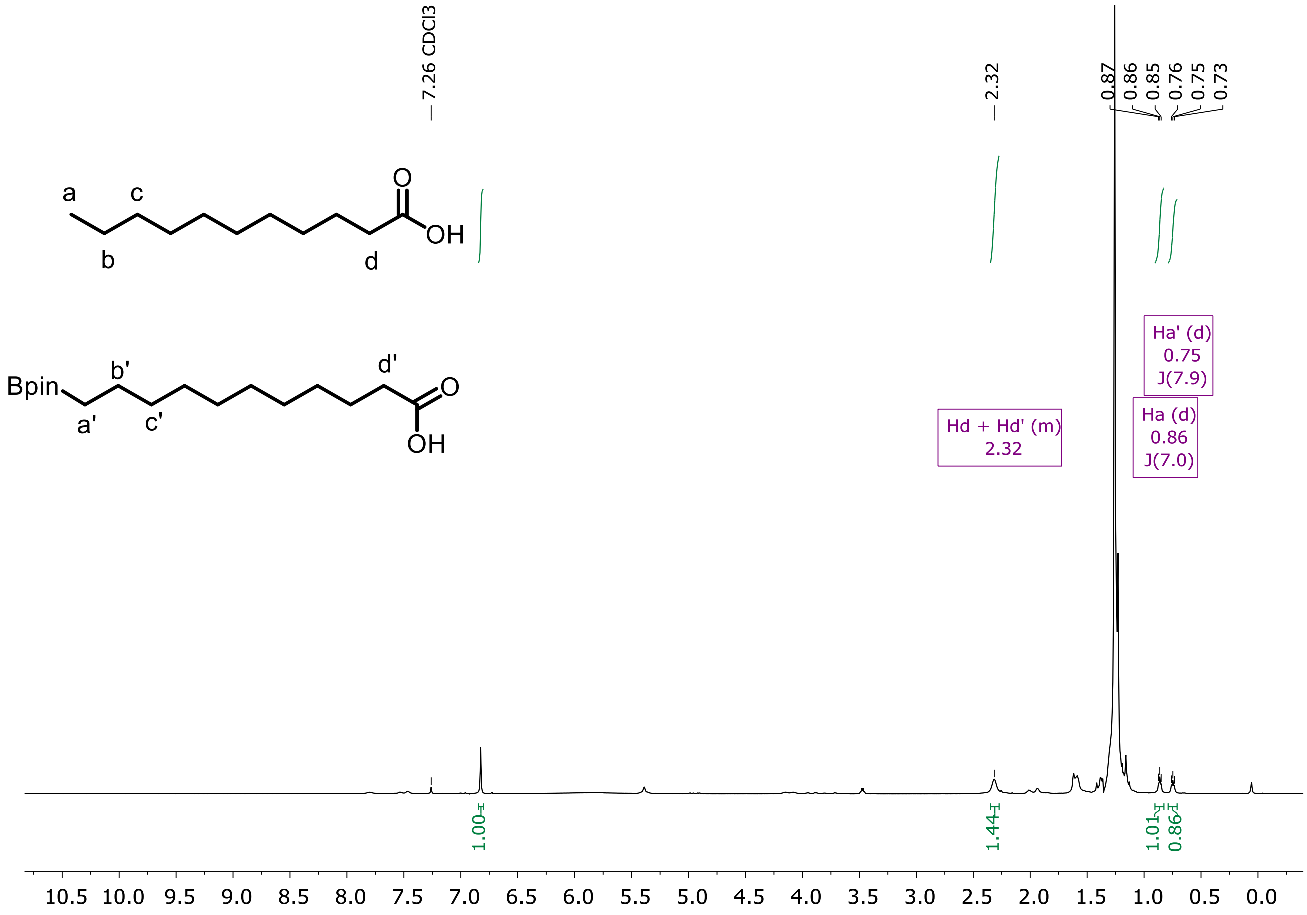

(*E*)-1-Phenyl-3-(4-(2-(4,4,5,5-tetramethyl-1,3,2-dioxaborolan-2-yl)vinyl)phenyl)propan-1-one (3p)

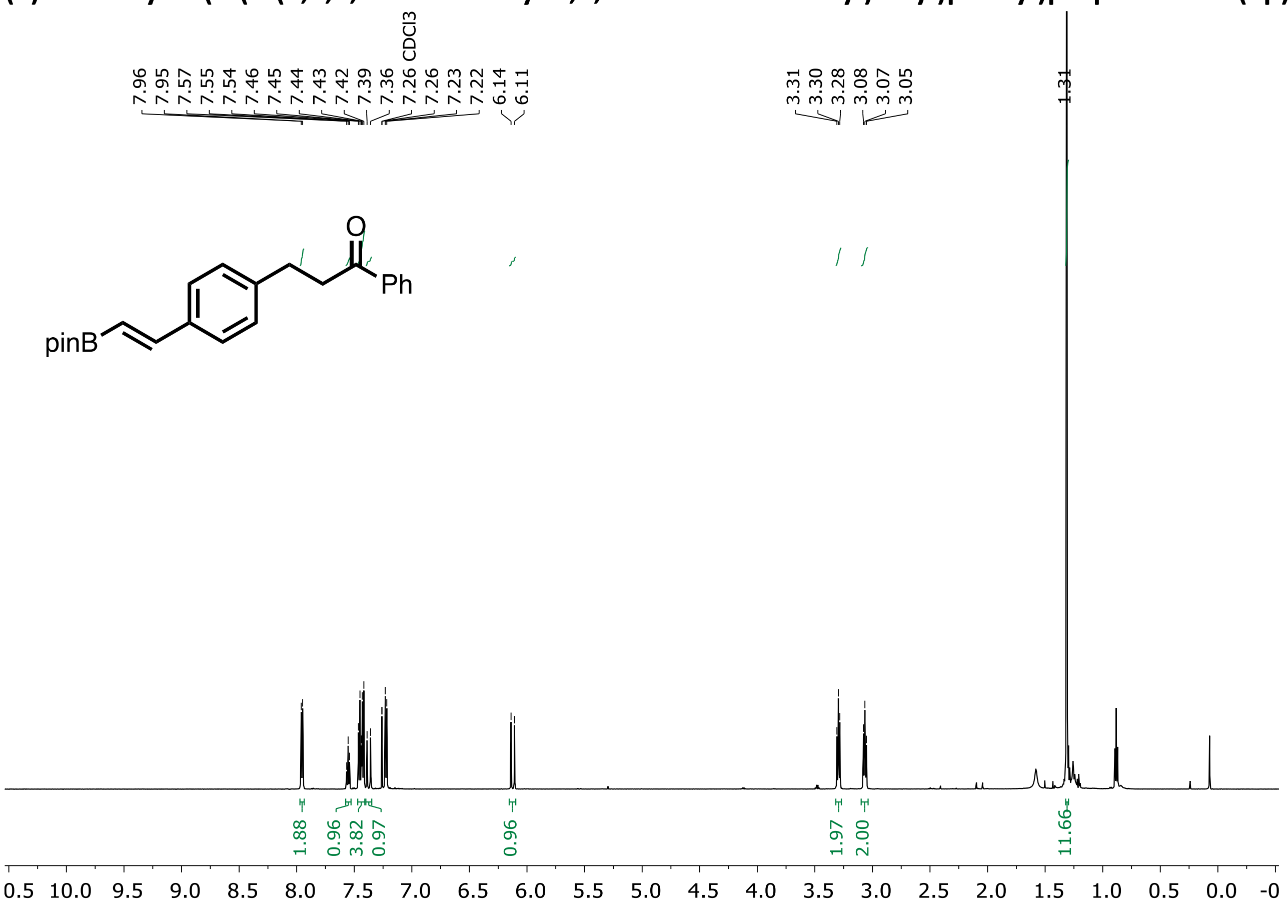

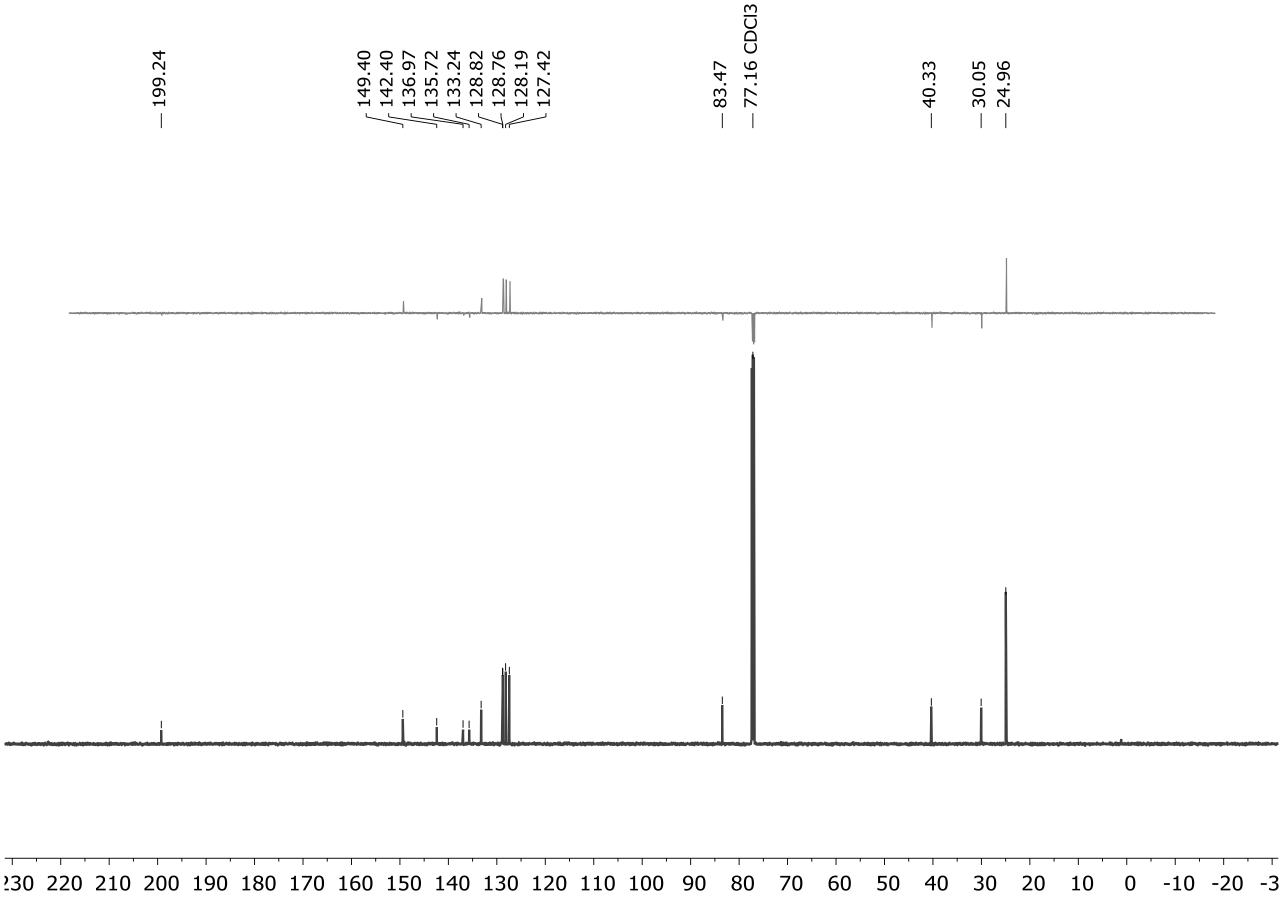

10. References

1) Y. Hua, H. Bin, Tao Wei, H. Cheng, Z. Lin, X. Fu, Y. Li, J. Xie, P. Yan, Q. Zhou, *Org. Lett.* **2020**, *22*, 818–822.

2) B. Lansbergen, C. S. Meister, M. C. McLeod, *Beilstein J. Org. Chem.* **2021**, *17*, 404–409.

3) T. Gieshoff, U. Chakraborty, M. Villa, A. J. von Wangelin, *Angew. Chem. Int. Ed.* **2017**, *56*, 3585–3589; *Angew. Chem*. **2017**, *129*, 3639-3643.

4) G. Meng, M. Szostak, *Angew. Chem. Int. Ed.* **2015**, *54*, 14518–14522; *Angew. Chem*. **2015**, *127*, 14726-14730.

5) N. R. Vautravers, D. D. Regent, B. Breit, *Chem. Commun.* **2011**, *47*, 6635–6637.

6) P. P. Thottumkara, T. K. Vinod, *Org. Lett.* **2010**, *12*, 5640–5643.

7) X. Chen, X. Ning, Y. Biao Kang, *Org. Lett.* **2016**, *18*, 20, 5368–5371.

8) B. Wozniak, Y. Li, S. Tin, S., J. G. de Vries, *Green Chem.* **2018**, *20*, 4433–4437.

9) J. H. Xie, L. C. Guo, X. H. Yang, L. X. Wang, Q. L. Zhou, *Org. Lett.* **2012**, *14*, 4758–4761.

10) K. Maeda, Y. Obora, S. Sakaguchi, Y. Ishii, *Bull. Chem. Soc. Jpn.,* **2008**, *81*, 689–696.

11) A. A. Singh, J. A. Rowley, B. D. Schwartz, W. Kitching, J. J. De Voss, *J. Org. Chem.* **2014**, *79*, 7799–7821.

12) M. Liu, W. Tang, M. Li, D.L. Phillips, *J. Org. Chem.* **2017**, 82, 3425–3431.

13) R. R. Behera, R. Ghosh, S. Panda, S. Khamari, B. Bagh, *Org. Lett.* **2020**, 22, 3642–3648.

14) X,. Xu, Z. Li *Organic Letters* **2019**, *21*, 5078-5081.

15) L. Zhou, X. Liu, J. Ji, Y. Zhang, W. Wu, Y. Liu, L. Lin, X. Feng,* *Org. Lett.* **2014**, *16*, 3938–3941.

16) C. Curti, N. Brindani, L. Battistini, A. Sartori, G. Pelosi, P. Mena, F. Brighenti, F. Zanardi, D. Del Rio, *Adv. Synth. Catal*. **2015**, *357*, 4082 –4092.

17) M. Allegretti, R. Bertini, M. C. Cesta, C. Bizzarri, R. Di Bitondo, V. Di Cioccio, E. Galliera, V. Berdini, A. Topai, G. Zampella, V. Russo, N. Di Bello, G. Nano, L. Nicolini, M. Locati, P. Fantucci, S. Florio, F. Colotta, *Med. Chem.* **2005**, *48*, 4312–4331.

18) H. Jung, S. Chang, S. Hong, *Org. Lett.* **2019**, *21*, 7099–7103.

19) . J. Wu, L., A. Noble, V. K. Aggarwal, *J. Am. Chem. Soc.* **2018**, *140*, 10700–10704.

20) R. Sang, P. Kucmierczyk, R. Du¨hren, R. Razzaq, K. Dong, J. Liu, R. Franke, R. Jackstell and M. Beller, *Angew. Chem., Int. Ed.*, **2019**, *58*, 14365–14373; *Angew. Chem*. **2019**, *131*, 14503-14511.

21) M. Onishi *J. Mol. Catal.* **1993**, *80*, 145–149.
